# Distribution of ancestral proto-Actinopterygian chromosome arms within the genomes of 4R-derivative salmonid fishes (Rainbow trout and Atlantic salmon)

**DOI:** 10.1186/1471-2164-9-557

**Published:** 2008-11-25

**Authors:** Roy G Danzmann, Evelyn A Davidson, Moira M Ferguson, Karim Gharbi, Ben F Koop, Bjorn Hoyheim, Sigbjorn Lien, Krzysztof P Lubieniecki, Hooman K Moghadam, Jay Park, Ruth B Phillips, William S Davidson

**Affiliations:** 1 Department of Integrative Biology, University of Guelph, Guelph, ON Canada, N1G 2W1; 2 Department of Molecular Biology and Biochemistry, Simon Fraser University, Burnaby BC, Canada, V5A 1S6; 3 Institute of Comparative Medicine, Faculty of Veterinary Medicine, University of Glasgow, Glasgow, Scotland, UK, G61 1QH; 4 Centre for Biomedical Research, Department of Biology, University of Victoria, Victoria BC, Canada,V8W 3N5; 5 Norwegian School of Veterinary Science, BasAM-Genetics, Oslo, Norway, NO-0033; 6 CIGENE – Centre of Integrative Genetics, Ås, Norway, NO-1432; 7 Institute of Animal and Aquacultural Sciences, Norwegian University of Life Science, Ås, Norway, NO-1432; 8 School of Biological Sciences, Washington State University, Vancouver, WA USA, 98686-9600

## Abstract

**Background:**

Comparative genomic studies suggest that the modern day assemblage of ray-finned fishes have descended from an ancestral grouping of fishes that possessed 12–13 linkage groups. All jawed vertebrates are postulated to have experienced two whole genome duplications (WGD) in their ancestry (2R duplication). Salmonids have experienced one additional WGD (4R duplication event) compared to most extant teleosts which underwent a further 3R WGD compared to other vertebrates. We describe the organization of the 4R chromosomal segments of the proto-ray-finned fish karyotype in Atlantic salmon and rainbow trout based upon their comparative syntenies with two model species of 3R ray-finned fishes.

**Results:**

Evidence is presented for the retention of large whole-arm affinities between the ancestral linkage groups of the ray-finned fishes, and the 50 homeologous chromosomal segments in Atlantic salmon and rainbow trout. In the comparisons between the two salmonid species, there is also evidence for the retention of large whole-arm homeologous affinities that are associated with the retention of duplicated markers. Five of the 7 pairs of chromosomal arm regions expressing the highest level of duplicate gene expression in rainbow trout share homologous synteny to the 5 pairs of homeologs with the greatest duplicate gene expression in Atlantic salmon. These regions are derived from proto-Actinopterygian linkage groups B, C, E, J and K.

**Conclusion:**

Two chromosome arms in *Danio rerio *and *Oryzias latipes *(descendants of the 3R duplication) can, in most instances be related to at least 4 whole or partial chromosomal arms in the salmonid species. Multiple arm assignments in the two salmonid species do not clearly support a 13 proto-linkage group model, and suggest that a 12 proto-linkage group arrangement (i.e., a separate single chromosome duplication and ancestral fusion/fissions/recombination within the putative G/H/I groupings) may have occurred in the more basal soft-rayed fishes. We also found evidence supporting the model that ancestral linkage group M underwent a single chromosome duplication following the 3R duplication. In the salmonids, the M ancestral linkage groups are localized to 5 whole arm, and 3 partial arm regions (i.e., 6 whole arm regions expected). Thus, 3 distinct ancestral linkage groups are postulated to have existed in the G/H and M lineage chromosomes in the ancestor of the salmonids.

## Introduction

Salmonid fishes are known to have descended from an autopolyploidization event that occurred within the ancestral grouping that gave rise to the Salmoniformes sometime within the early Tertiary or late Cretaceous periods [1]. Supporting evidence for this event is both karyotypic and genomic, in that salmonids still retain a large amount of duplicate gene expression within their genome. This is evidenced by the fact that essentially identical DNA sequence tracts are retained within their genomes which map to unlinked chromosomal arms (i.e., detection of several duplicated SSR and EST markers in their genome)[2-4]. Furthermore, quadrivalent meiotic configurations are often observed within male-specific meioses involving a portion of the available chromosome arms. These pairings always appear to involve metacentric chromosomes that may or may not include an acrocentric pair [5]. Segregation ratios consistent with tetrasomic inheritance or partial tetrasomic ratios are also observed following male meioses [6] indicating that the genomes of salmonids have not completely returned to disomy which would be expected following the complete divergence of duplicated chromosomal regions. Additionally, the modal range of chromosome arms in salmonids is between 96 – 104, whereas most teleost species, especially freshwater groups, have modal diploid chromosome numbers ranging from 48 – 52 [7,8]. This observation is strong supporting evidence that the salmonid genome underwent a whole genome duplication (WGD) from a freshwater ancestral lineage.

Actinopterygians are also now largely recognized as being descended from ancestral forms that underwent a WGD event. Two current models exist, which suggest that the ray-finned fishes arose from an ancestral form possessing either 12 [9] or 13 ancestral linkage groups [10]. Supporting evidence for the WGD event can be obtained from phylogenetic data on a large number of gene duplicates which estimate the divergence times for these genes somewhere between 275 – 350 MYA [9-13]. This 'burst' of gene duplication overlays data that demonstrates continuous levels of genomic duplication events throughout the evolutionary history of the group. Additional support comes from examining the number and distribution of functional gene copies that are retained in the genome of modern teleosts within highly conserved multi-gene families. Hox genes are a classic example of the postulated WGD event that occurred in the ray-finned fish lineage. All the teleost species that have been extensively studied at the DNA sequence level, retain 7 ancestrally derived Hox clusters (8 are expected) at different chromosomal locations, each with up to 13 genes, whereas invertebrate species typically contain only a single Hox cluster or separate genes interspersed throughout their genome [14,15]. The organization of the central 13 Hox paralogs into multiple syntenic clusters is strong evidence that Actinopterygians have experienced 3 WGD events in their evolutionary history. In the Sarcopterygian lineage that led to the tetrapods, only 4 extant Hox clusters are evident, suggesting that two rounds of WGD may have led to the formation of the lobe-finned clade (i.e., 2R duplication model). This 2R duplication is postulated to have occurred prior to the origin of the gnathostomes [11-13,15]. Actinopterygians, then are seen as 3R WGD descendants (i.e., having experienced 3 rounds of WGD in their evolutionary past). Support for this model comes from the fact that a vast majority of the 3R gene duplicates fall within large syntenic blocks that can be assigned to a pair of linkage groups or haploid chromosome sets within the genomes of extant teleost species. Within two salmonid species, rainbow trout (*Oncorhynchus mykiss*), and Atlantic salmon (*Salmo salar*) up to 14 Hox paralogons or chromosomal locations (up to 16 are expected) have been identified supporting the model that salmonids are 4R derivative lineages [16,17]. Thus, investigations within this more recent teleost polyploid lineage may provide important insights into the organization and distribution of ancient synteny blocks that gave rise to the modern day vertebrate lineages.

The most recent model on vertebrate evolution postulates that 10 different linkage group blocks existed in a proto-chordate ancestral form [10], that may be designated A' – J'. Following two rounds of WGD, involving multiple single chromosome duplications within the A', B', and F' lineages, (that gave rise to 17 chromosomes), and multiple chromosome loss or fusion events in the G', H', I', and J' lineages (that resulted in 11 chromosomes), a 40 chromosome ancestral form arose at the base of the gnathostome lineage. Extensive chromosomal fusions, and in some instances fissions within the ancestral gnathostome segments, were postulated to have led to a 13 chromosome proto-Actinopterygian karyotype composed of 52 A'-J' segments as follows: A' = 8 segments; B' = 10 segments; C' = 6 segments; D' = 5 segments; E' = 5 segments; F' = 6 segments; G' = 4 segments; H' = 3 segments; I' = 3 segments; and J' = 2 segments [10]. In most instances these blocks became mosaically arrayed within the 13 chromosome proto-Actinopterygian karyotype (designated A – M), with the exception of the 3 group I' blocks that appear to have been arrayed tandemly towards one end of the E chromosome in the proto-Actinopterygian karyotype, and the K chromosome which was composed entirely from a single block within the D' lineage. These arrangements are of interest due to the fact that herein we report that synteny blocks derived from the E and K ancestral chromosomes along with three other ancestral linkage groups appear to retain a high degree of duplicate sequence expression within the genomes of both rainbow trout and Atlantic salmon. The current dataset also suggests that large tracts of extensive synteny appear to exist between the salmonids and model teleost test species such as the zebrafish (*Danio rerio*) and medaka (*Oryzias latipes*), and thus, comparative synteny analyses may be extremely valuable in identifying genes of importance regulating physiological, developmental, and behavioural modalities in salmonid fishes.

## Results

### Genetic maps

Currently there are 1855 markers located onto the L25 and L44 rainbow trout mapping panels. These include: 727 AFLP markers; 102 Type I genes; 337 EST markers; 665 SSR markers; 21 BAC-end sequence markers; and the phenotypic marker SEX. There are presently 1671 markers localized to the Br5 and Br6 Atlantic salmon mapping panels. These markers are distributed as: 275 AFLP markers; 43 Type I genes; 174 Type I-SNP markers; 187 EST markers; 410 SSR markers; 582 BAC-end sequence markers; and the phenotypic marker SEX. Information on the distribution of variation in these markers within the four mapping parents used for each species is given in Additional File 1 and Additional File 2. The genetic maps for the four rainbow trout mapping parents are provided in Additional Files 3, 4, 5, 6, while the respective maps for the four Atlantic salmon parents are provided in Additional Files 7, 8, 9, 10. Additional File 11 depicts the combined rainbow trout female map aligned with the homologous Atlantic salmon linkage groups, while Additional File 12 depicts the reciprocal associations. In both species maps, linkage group 1 represents the sex-linkage group. Given the large recombination differences between the sexes in salmonid species [2,18,19], female maps were deemed the most representative of true gene marker orders within each species, and merged female maps were constructed for all comparative analyses.

### 4R versus 3R homologies

**Figure 1:**
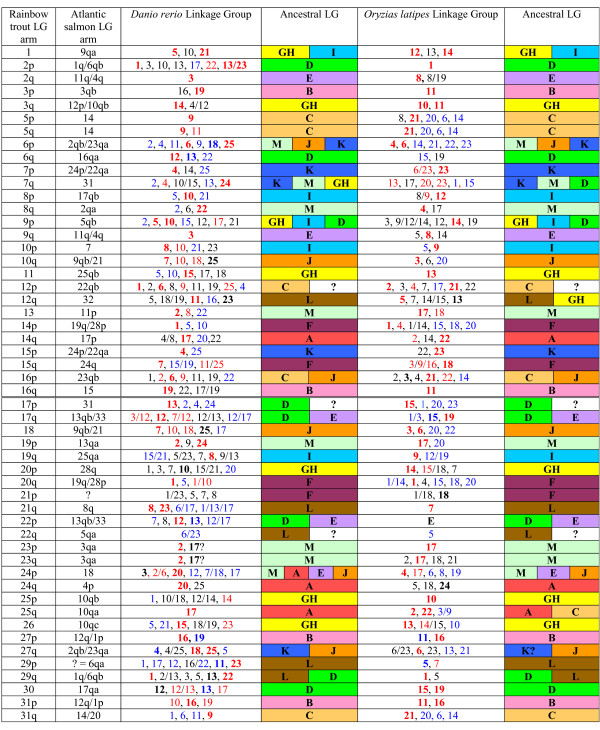
**Distribution of 3R teleost (*Danio rerio *and *Oryzias latipes*) homologies within rainbow trout and Atlantic salmon linkage groups.** The 13 ancestral linkage groups are designated according to Nakatani et al. 2007 [10], with two linkage groups (GH) examined as a single contributing region, assuming a 12 linkage group ancestral model [9]. Homologies detected solely in rainbow trout are depicted in black font. Homologies solely detected in Atlantic salmon are shown in blue font, while, shared homologies within both salmonid species are indicated in red font. Synteny blocks detected within salmonid linkage groups are shown in **bold **font, according to the color designations indicated.

**Figure 2:**
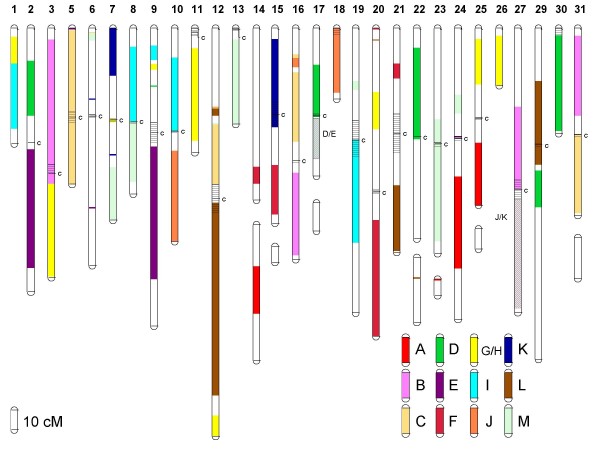
**Affinities of the 12/13 ancestral proto-Actinopterygian linkage groups [A – M] based upon the Kasahara et al.** model [20] within the rainbow trout genome. Composite female map lengths are given in centiMorgans (cM) according to the scale shown in the bottom left-hand corner of the figure. In some instances female linkage groups are depicted as multiple separate LOD = 4.0 clusters. Centromeric locations within each linkage group [38] are shown as horizontal line segments and are indicated with a 'c' to the right of the linkage group. For certain linkage group arms, it is currently not possible to distinguish D and/or E ancestral affinities, or J and/or K ancestral affinities. These arm segments are indicated as D/E, and J/K regions on the figure and are indicated via diagonal cross-hatching. Short *p *arms are oriented towards the top of the figure according to [22].

**Figure 3:**
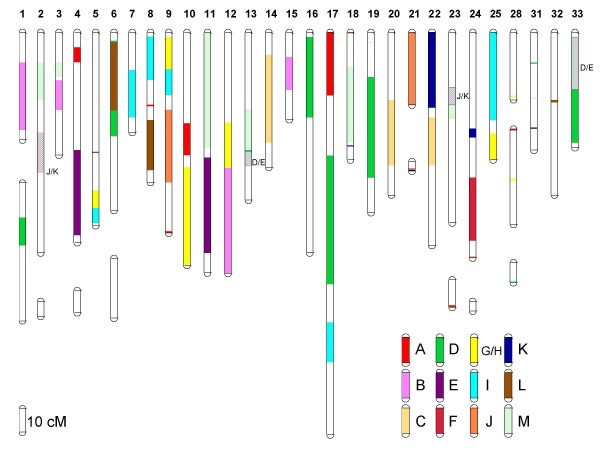
**Affinities of the 12/13 ancestral proto-Actinopterygian linkage groups [A – M] based upon the Kasahara et al.** model [20] within the Atlantic salmon genome. Composite female map lengths are given in centiMorgans (cM) according to the scale shown in the bottom left-hand corner of the figure. In some instances female linkage groups are depicted as multiple separate LOD = 4.0 clusters. For certain linkage group arms, it is currently not possible to distinguish D and/or E ancestral affinities, or J and/or K ancestral affinities. These arm segments are indicated as D/E, and J/K regions on the figure and are indicated via diagonal cross-hatching. Short *p *arms are oriented towards the top of the figure according to [21].

As expected, the assignment of linkage groups within the two salmonid species to the ancestral ray-finned fish karyotype indicates that most ancestrally derived 3R linkage groups are represented by approximately 4 whole or partial arm affinities in the salmonids (Table 1 and Figures 1, 2 and 3). Both the G/H and M ancestral linkage groups have the highest number of detected salmonid arm homologies across all linkage groups, with 5 whole arm, and 3–4 partial arm affinities in both sets. Linkage group M is postulated to have undergone a single chromosome duplication event that gave rise to 3 linkage groups shortly after the origin of the ray-finned fish lineage [10,20]. Following a WGD six linkage group affinities would be expected in a 4R lineage which is consistent with the empirical data within archetype group M. For linkage group G/H, the current data is compatible with an interpretation that this assemblage could have arisen from two ancestral linkage groups. According to the Kasahara *et al*. model [20], linkage group G primarily composes Dr14 and Olat10/14, with minor contributions to Dr15, and linkage group H contributes primarily to Dr10/15/21 and Olat13. This would then be compatible with the interpretation that all of, or portions of linkage group arms RT1/AS9qa, RT7q/AS31, RT9p/AS5qb, RT11/AS25qb, RT12q/AS32, and RT26/AS10qc descended from basal group H, while RT1/AS9qa, RT9p/AS5qb, RT3q/AS10qb, RT3q/AS12p, RT20p/AS28q, and RT25p/AS10qb are most likely to have descended from group G. Because of the shared affinities among these three putative ancestral linkage groups (i.e., G, H, I), it is currently unclear in the salmonids whether these groupings arose from WGD events in three separate proto-Actinopterygian linkage groups, or whether one or more of the single linkage groups within this set underwent a single chromosome doubling followed by genomic re-arrangements within this set of genomic segments. This would suggest that these genomic regions were derived from only two ancestral groupings (i.e., a 12 chromosome proto-Actinopterygian model; see Discussion for explanation).

**Table 1:** Ancestral linkage group arm distributions within the salmonid genome.

Ancestral Linkage Groups	*Danio rerio *linkage groups^1^	*Oryzias latipes *linkage groups^1^	Number of whole linkage group arms^4^	Number of partial linkage group arms^4^	Partial arms syntenic only in *Drer *or *Olat*
A	17, 20	22, 24	2	1	1
B	16, 19, (1)^3^	11, 16	4	0	0
C	6, 9 (11)	2, 21	3 (2 = 1)^2^	2	1
D	12, 13, (17, 20, 1)	15, 19 (1)	3	5	1
E	3, 12, (1)	8, 19, (1)	2	3	0
F	1, (7)	1, 18 (10)	4	0	0
G H	14, 15, 10, 21, (1, 5)	13, 14 (10, 11)	5	2	2
I	5, 8, 10, 21	9, 12	3	2	0
J	7, 18, 25, (19)	3, 6	2	4	0
K	4, (18, 24, 25)	6, 23	2	3	0
L	11, 23 (6, 8, 17, 22, 24)	5, 7	2	3	0
M	2, 22, 24 (6, 8, 11)	4, 17, 20 (13)	5	3	0

^1 ^Linkage group arm affinities within zebrafish and medaka to their ancestral origins are from the model presented by Nakatani et al. 2007 [10], and data from the current study. The number of whole and partial arm affinities to these chromosomal regions in the two salmonid species are indicated in the last 3 columns.

^2 ^RT-5 represents a putative whole arm inversion (i.e., 2 of the whole arms indicated may only represent one ancestral arm).

^3 ^Linkage groups specified in parentheses indicate descendancy of only minor ancestral chromosomal segments within the linkage groups specified.

^4 ^Indicates that number of whole or partial chromosome arms in rainbow trout/Atlantic salmon that are syntenic with the ancestral linkage group designated in the first column.

Although many large synteny blocks were detected in the salmonids among the various 3R teleost linkage groups (i.e., within zebrafish and medaka) (Figures 2 and 3, and Additional File 13 (rainbow trout synteny blocks), and Additional File 14 (Atlantic salmon synteny blocks)), many ancestral affinities were not readily apparent until linkage group blocks were assembled according to their ancestral homologies (e.g., compare RT9p, RT12p and RT16p affinities to zebrafish and medaka chromosomes shown in Additional File 13 with the ancestral homologies in Figure 2). Additionally, within salmonid linkage group arms retaining major synteny to whole 3R teleost chromosome arms (e.g., RT9q with Dr3 and Olat8 – Figure 2 and Additional File 13), marker positions according to the 3R species database assemblies in ENSEMBL were not linear, suggesting major internal re-arrangement and inversions have occurred within the homologous chromosomes among these species. Within several arms, homologies were also detected to multiple ancestral linkage groups (e.g., RT6p, RT7q, RT9p, RT12p, RT17p, RT24p and their respective Atlantic salmon homologues), and single-marker assignments to additional groupings are also evident, suggesting certain linkage group arms in the salmonid genome may even be more mosaic than currently depicted.

### 4R homeologies within Atlantic salmon and rainbow trout

**Figure 4:**
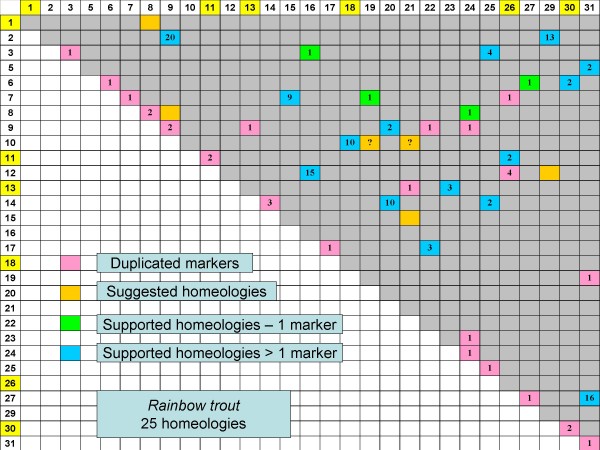
**Putative 4R homeologous linkage group affinities within the rainbow trout (Figure 4) and Atlantic salmon (Figure 5) genome based upon duplicated marker distributions.** Acrocentric linkage groups composed of only a single ancestral arm are depicted in yellow background on the Oxford grid axes. Acrocentric linkage groups are expected to only possess a single 4R homeologous affinity while metacentric linkage groups should possess at least two 4R affinities. Several arm fusions have occurred in Atlantic salmon resulting in the expectation that metacentric chromosomes such as AS-17 possess 3 whole-arm affinities, while acrocentric chromosomes AS-2, -3, -5, -6, -8, -9, -13, -16, -22, -23, and -25 will have two whole-arm affinities. For AS-10, three whole-arm affinities are expected within this large acrocentric chromosome [21]. Homeologies based upon only a single duplicated marker are in some instances supported by the mapping of single non-duplicated markers in alternate mapping parents to either of the putative duplicated homeologous linkage groups. Inferred homeologies based upon the comparative synteny mapping with zebrafish and medaka (Figures 1 – 3) are indicated as orange blocks in the Oxford grids.

Given the fact that salmonids have undergone a more recent WGD compared to most extant teleosts, it is expected that whole chromosome arms should retain duplicated marker signatures of this event. The fact that most salmonid species retain between 96 to 104 chromosome arms is also strong support that a WGD has occurred within this lineage given the observation that the modal chromosome number in teleosts is 48–50 acrocentric based chromosomes yielding 24–25 linkage groups. Thus, in salmonids we might expect a similar number of homeologous affinities to be observed (i.e., 24 up to a maximum of 27 homeologs [= pairs of chromosome arms derived from the most recent WGD]), dependent upon the number of extant chromosome arms in the species and whether individual chromosome arms may have experienced pericentric inversions during their evolutionary history. Multiple arm re-arrangements and translocations may also increase the number of arm homeologies observed, as a single chromosome arm may have affinities to more than one other chromosome arm in the genome. Such a situation is evident for the 9p arm and centromeric region of RT9, wherein multiple single marker putative homeologies have been observed. When considering syntenic block homeologies within rainbow trout, 20 linkage group arm pairs have been observed to possess either more than one duplicated marker, or in the case where only a single duplicated marker has been observed there is supporting evidence from the 3R syntenic blocks that these regions are homeologous (Figure 4). Five additional homeologues are inferred from the ancestral 3R synteny blocks. However, for two of these putative homeologues (i.e., RT8/24 and RT23/24), the marker affinities may lie with the same region on RT24p possessing ancestral M homology. Also, for the affinities inferred between RT1/8 and RT8/9, it cannot be discounted that RT1/9 may also share homeologies, and therefore, these putative blocks may only represent a single homeologous grouping. Therefore only 23 homeologies can be inferred. We have not yet detected affinities to ancestral homologies within four linkage group arms in rainbow trout (21q, 22q, 23p, and 24q), and thus the final expected minimum number of homeologues should be 25. Also RT5 appears to represent a pericentric inversion represented entirely by ancestral linkage group C.

**Figure 5:**
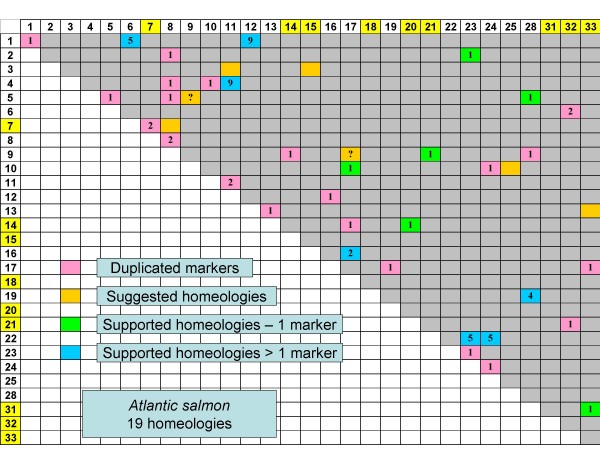
**Putative 4R homeologous linkage group affinities within the rainbow trout (Figure 4) and Atlantic salmon (Figure 5) genome based upon duplicated marker distributions.** Acrocentric linkage groups composed of only a single ancestral arm are depicted in yellow background on the Oxford grid axes. Acrocentric linkage groups are expected to only possess a single 4R homeologous affinity while metacentric linkage groups should possess at least two 4R affinities. Several arm fusions have occurred in Atlantic salmon resulting in the expectation that metacentric chromosomes such as AS-17 possess 3 whole-arm affinities, while acrocentric chromosomes AS-2, -3, -5, -6, -8, -9, -13, -16, -22, -23, and -25 will have two whole-arm affinities. For AS-10, three whole-arm affinities are expected within this large acrocentric chromosome [21]. Homeologies based upon only a single duplicated marker are in some instances supported by the mapping of single non-duplicated markers in alternate mapping parents to either of the putative duplicated homeologous linkage groups. Inferred homeologies based upon the comparative synteny mapping with zebrafish and medaka (Figures 1 – 3) are indicated as orange blocks in the Oxford grids.

The homeologies within Atlantic salmon are dependent both upon single arm affinities as well as partial arm affinities due to the whole arm fusions that have occurred within this species. Thirteen linkage groups within this species possess whole arm fusions of at least two chromosome arms [20], with two of these linkage groups possessing 3 whole arm affinities to rainbow trout. Atlantic salmon possess far fewer duplicated genetic markers compared to rainbow trout. The lack of duplicated polymorphisms within this species makes it difficult to detect homeologies. Only 5 homeologs were detected with an abundance of duplicates (≥ 5) (Figure 5), and in total, only 19 homeologies are described, with 6 of these putative homeologs based upon inferences from the ancestral linkage group affinities. Furthermore, AS5qb/AS9qa and AS17qb/9qa represent possible partial arm homeologies, as do the associations between AS24q/8qa and 8qa/9qa. Therefore only 17–18 homeologies can be inferred given that affinities within AS8qa and AS9qa appear to represent partial arm homeologies. Homeologous affinities for AS1qb, 2qb, 4p, 5qa, 6qb, 8qb, 10qb, 12p, 13qa, 16qb, 18, 24q, 28p, 25qa, and a portion of 31 could not be inferred based upon larger synteny blocks. Thus, similar to rainbow trout, it is expected that at least 25 homeologs should eventually be identified, once the 7–8 homeologous affinities represented by these unassigned chromosome arms can be identified.

## Discussion

Salmonid genomes have an architecture that is largely reflective of WGD events in their evolutionary past. When considering only the larger synteny blocks that have been tentatively identified within rainbow trout and Atlantic salmon genomes, all ancestral linkage groups with the exceptions of group A align with whole or partial arm affinities representing 4 or more arms. Following two rounds of WGD, four homologous affinities would be expected across the salmonid chromosome arms each possessing homology to a single pre-3R ancestral region. Regions homologous to ancestral grouping A have only been detected within 2 apparent whole arms (i.e., RT14q and RT24q), and one partial arm (RT25q). A fourth region of homology may reside on RT24p, but this has not been confirmed through synteny mapping within the medaka genome. Greater than 4 whole arm affinities exist for homologies detected with ancestral groupings G/H and M. If G/H do in fact represent two separate ancestral linkage groups as postulated by the Kasahara et al. [20] model then 8 arm affinities should exist in salmonids for these linkage groups. We cannot exclude this possibility with the current dataset, given the fact that 5 whole-arm and 2–4 partial-arm syntenies were identified as being derived from the G/H cluster. However, the comparative homologies with zebrafish chromosomes derived from G/H/I ancestral groupings represent a mosaic pattern across the salmonid genome, which may also be compatible with the model whereby a single chromosome within either the ancestral putative G/H grouping or I grouping underwent a duplication followed by reticulate exchange of segments between all the chromosomes in these two (i.e., G/H vs I) lineages (see details below). This same scenario appears to account for the three M lineage derived chromosomes within both the medaka and zebrafish genomes [10], and is supported by the data within the salmonids, given that 5 whole-arm and 3 partial-arm affinities exist for M lineage markers. Finally, the assignment of 3 whole-arm, and 5–6 partial-arm affinities to D lineage derived chromosomes within salmonids is more a reflection of the very incomplete information contained within certain salmonid linkage group arms. For example, while both comparative synteny mapping and duplicated marker expression support the homeologies between RT6q/RT30, the region within RT6q possessing homology to ancestral region D is currently very small. RT6 is the largest physical chromosome in the genome [21,22], and it would not be surprising to find other partial-arm affinities within RT6q.

Multiple partial arm affinities may be expected through time, following translocation and genome re-arrangement events within each species. While it can be postulated that as more comparative data is accumulated the chromosome arm affinities currently assigned to an ancestral grouping will be represented by a more mosaic association pattern among chromosome arms, the current data allows us to observe certain whole arm affinities within linkage groups that appear to have arisen by the fusion of arms within the same ancestral groupings. For example, RT30 and its homologous region AS17qa show extensive homology to group D throughout the length of the currently identified linkage group markers. Using the comparative medaka genome as a template, it can be observed that both Olat15 and Olat19 markers form two large synteny blocks within RT30, suggesting a possible fusion of group D 3R duplicates within this region of the salmonid genome. Conversely, Olat19 contributes most extensively to the RT9p/RT17q/RT22p affinities, while Olat15 underlies the RT6q and RT17p affinities (Figure 1). Similarly, AS33 also appears to represent a fusion of group D segments as the largest portion of the acrocentric chromosome is derived from Olat19 (with affinities to RT22p/RT17q), with a smaller terminal region distal to BX075694 (see Table 1 and Additional File 13 and Additional File 14) derived from Olat15, and sharing homology with RT17q. Both these regions on AS33 are divided by a smaller synteny block derived from group E.

Two ancestral linkage groups (H and I) appear to share a propensity in forming adjacent synteny blocks within chromosome arms, as indicated by their partial arm affinities within RT1/AS9qa, RT9p/AS5qb, and the whole arm fusions represented by AS25qa and AS25qb. A small region near the centromere on RT12q also possesses a synteny block derived from ancestral grouping H. These observations are of interest given the finding by Kasahara et al. [20] that ancestral groupings H and I have contributed extensively to chromosome re-arrangements within zebrafish linkage groups Dr14, Dr15, Dr10, Dr21, Dr5, and Dr8. Conversely, linkage groups derived from the ancestral H/I groups were not suggestive of rearrangements within either medaka or the greed-spotted pufferfish (*Tetraodon nigriviridis*) genomes [20]. This observation suggests that chromosomal re-arrangements in these two ancestral groupings may have been more extensive within at least some of the more basal Malcopterygian (soft-rayed) teleosts.

Similar to the single ancestral chromosome doubling within the M linkage group that was postulated to account for the retention of large synteny blocks among 3 modern day teleost linkage groups [10,20], the current data obtained from the salmonids, in combination with the genomic arrangements detected in zebrafish, suggests that one of the chromosomes within the G/H/I linkage groups underwent a single chromosome doubling event. Following this single duplication, it is unclear whether two of these chromosomes may have undergone fission events to generate 4 new chromosomes, or whether only 1 of the newly formed chromosomes may have undergone a fission event. With the former scenario, following extensive meiotic exchange among these regions, a subsequent fusion event may have occurred between a pair of these chromosomes. Evidence supporting this hypothesis is obtained using comparative syntenies within salmonids is as follows: Within the salmonids the following zebrafish and medaka homology pairings were observed within linkage group arms: Dr8 with Olat9, 2×; Dr5/10/21 with Olat12, 3×, Dr5/10/15 with Olat13, 1×; Dr5/10/21 with Olat14, 2×; Dr14 with Olat10, 2×. The observation of associations within linkage groups derived from the I cluster within medaka (i.e., Olat12), and the G/H cluster (i.e., Olat14/13), with segments derived from Dr5/10/21, and Dr15 with Olat13, suggests that extensive homology exists among all these regions.

**Figure 6:**
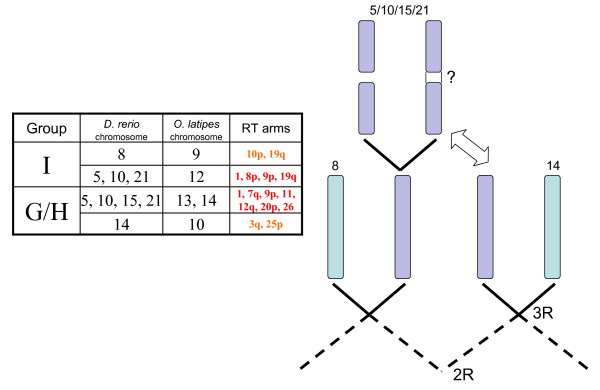
**Model of ancestral linkage group re-arrangements among G/H/I lineage chromosomes.** Extensive cross syntenies among zebrafish chromosomes 5, 10, 15, and 21 suggest that a duplication and subsequent fission of one of the two 3R chromosomes in either the I, or presumptive G/H lineage may have occurred. This fission would give the appearance of extra ancestral groupings (i.e., greater than 4 linkage group arm homologies to either the G/H or the I lineage chromosomes). Reticulate exchange among segments from both ancestral groupings may also have occurred as evidenced by the retention of G/H/I synteny blocks within rainbow trout arms 1 and 9p. Rainbow trout chromosome arms possessing multiple affinities to zebrafish linkage groups 5/10/15/21 (i.e, suggestive of greater re-arrangements), are indicated in red font in the centre of the table.

Ancestral groupings G/H/I share segments from 3 (i.e., C', F', and H') of the 10 linkage group blocks of the postulated proto-chordate genome clusters [10]. From this model, it is most likely that a chromosome within the H cluster underwent a single duplication and consequent fission, as this may have generated segments sharing homology among proto-chordate C'/F' blocks among the G/H groups, and proto-chordate C'/H' blocks between the H/I groups. There appears to have been a high degree of exchange between these regions and one of the duplicated chromosomes in the I Group (homologous to Olat12). Presumably one of the derived chromosomes in each grouping (presumptive Dr8 and Dr14, respectively) were under greater evolutionary constraint, which limited (or selected against) exchanges within these blocks. However, exchanges appear to have been prevalent among one chromosome from each of the ancestral (G/H)/I pairs, as evidenced by their mosaic distributions across the salmonid and zebrafish genomes (see Figure 6 and Additional File 15 for a further explanation of the model). Thus, the current dataset suggests that a 12 linkage group proto-karyotype proposed for the Actinopterygian fishes [9], may be more consistent with the observed distribution of linkage group affinities within the lower teleost species currently studied.

### Distribution of duplicated markers

Far fewer duplicated markers were identified in Atlantic salmon (9.5%) compared to rainbow trout (26%), despite both genetic maps currently having approximately the same number of informative type I and type II marker distributions (i.e., 1125 and 1222 potentially informative markers in rainbow trout and Atlantic salmon, respectively, excluding SNP markers which were pre-screened to remove duplicates). A greater percentage of type I gene and EST-based markers were analyzed in rainbow trout, however, compared to Atlantic salmon which may indicate that duplicate expression is retained to a greater degree within coding vs. anonymous DNA. Another complementary explanation for this phenomenon may be the lack of recombination experienced within the Atlantic salmon genome (i.e., males have dramatically reduced recombination rates compared to females [18,19,23]). Within the current dataset, the female : male recombination ratios based upon all pairwise associations of genetic markers within linkage groups ranged from 7.050:1 to 7.234:1 in the two Atlantic salmon mapping families, and 2.567:1 to 2.624:1 in the two rainbow trout mapping families. Although recombination levels are generally suppressed in all male salmonids, the degree of recombination suppression appears most extensive within Atlantic salmon, a species that has undergone extensive genome re-arrangements involving the production of metacentric chromosomes and large acrocentric chromosomes created via whole arm chromosomal fusions, in comparison to other salmonid species which have experienced more moderate rearrangements (i.e., rainbow trout possessing multiple metacentric chromosomes, or Arctic charr (*Salvelinus alpinus*) possessing a few metacentric chromosomes) [7]. While chromosome structure partially accounts for the large differences observed in female : male recombination ratios, with lower ratios observed within acrocentric-based chromosome lineages [23] (i.e., more uniform male and female recombination rates), physical chromosome structure may not entirely define the degree of retention of duplicated gene expression and differential sex-specific recombination rates. Other factors such as gene arrangements within chromosome arms, and DNA structure motifs contributing to the formation of chiasmata points [24], or even sex-specific regulation of cross-over foci [25] may also be contributing factors that are currently poorly understood in these fishes.

Within Atlantic salmon, the five homeologous pairs of linkage groups exhibiting the highest level of duplicated marker expression (≥ 5 pairs) (i.e., AS1/6; AS1/12; AS4/11; AS22/23; AS22/24) are all localized within either metacentric or larger fused acrocentric chromosomes [21]. Similarly, in rainbow trout, there are 7 homeologous pairings (i.e., RT2q/RT9q; RT27p/RT31p; RT2p/RT29q; RT7p/RT15p; RT12p/RT16p; RT14p/RT20q; and RT10q/RT18) that are represented by even greater numbers of duplicated marker affinities, and all of these regions, with the exception of RT18, fall within metacentric type chromosomes. We suggest that these regions may retain a higher percentage of duplicated marker expression due to the fact that both intra-homologous and inter-homeologous recombination levels may be increased among these linkage groups. The distribution of recombination nodes within these linkage groups will, however, require additional cytogenetic investigation.

Increased recombination among homeologs will lead to increased frequencies of multivalent formation during meiosis which often generates pseudolinkage affinities among ancestral homeologues involved in the pairing [5]. Interestingly, in our mapping panels, some of the homeologous linkage groups that exhibit the highest retention of duplicate marker expression have also been detected forming putative pseudolinkage affinities within rainbow trout mapping parents (i.e., RT2/29; RT2/RT9; RT12/RT16; RT27/31) [[23]; unpublished data ], although pseudolinkage affinities have also been observed between homeologous pairs with lower levels of duplicate gene expression (i.e., RT6/30; RT5/31). Conservation of pseudolinkage affinities is apparent across salmonid species in that 5 pseudolinkage groupings have been identified in brown trout (i.e., BT1/8; BT5/15; BT3/10; BT11/12; and BT4/34) [4] that can be assigned to homologous chromosomal regions (i.e., RT29/2; RT15/7; RT12/16; RT31/27; and RT14/unknown, respectively) in rainbow trout. Thus, all 4 of the linkage group pairs with ascertained arm assignments between rainbow trout and brown trout correspond to rainbow trout linkage groups with a high degree of duplicate gene expression. In contrast, pseudolinkage occurrence appears to be more minimal in Atlantic salmon. One of our mapping panel males has a pseudolinkage grouping between AS5/18 at a LOD 4.0 clustering level, which appears to be homologous to a RT9/24 region (i.e., is supported by evidence for a couple of duplicated markers).

More extensive arm re-arrangements within the genome of Atlantic salmon may limit the occurrence of multivalents in this species. Strain background may also influence the propensity to express pseudolinkage given that recombination rates appear more elevated in inter-strain hybrid versus pure strain genomic backgrounds [26]. Therefore, the probability of detecting pseudolinkage affinities in Atlantic salmon may be decreased due to the fact that all four Atlantic salmon parents used in this study are of pure strain origin, while two of the four rainbow trout parents used were of inter-strain origin, and one male was derived from an intra-strain cross involving a mating between temporally divergent spawning families (i.e., using cryo-preserved sperm).

The concept that the surrounding genetic structure within an ancestral genomic region may be extremely important in defining the propensity to retain duplicated gene copies can be observed within the current dataset. All five of the regions retaining duplicate gene expression patterns in Atlantic salmon, share affinity to the same ancestral teleost regions within rainbow trout genome, and are syntenic with the same chromosome arm in rainbow trout (i.e., RT2q/RT9q & AS4q/AS11q = group E affinity; RT27p/RT31p & AS1p/AS12q = group B affinity; RT2p/RT29q & AS1q/AS6qb = group D affinity; RT7p/RT15p & AS22qa/AS24p = group K affinity; and RT12p/RT16p & AS22qb/AS23qb = group C, J affinity. While further research is clearly needed to understand the underlying nature of these enhanced duplicated gene regions, there are several factors that may contribute to the observed distributions. One explanation is related to transposable element clusters that have been reported to be associated with recombination 'hot-spots' in that region-specific cross-over frequencies appear to be negatively correlated with the occurrence of transposable elements in these regions [27-29]. These elements have ubiquitous effects on underlying genomic structure, including alterations in the rates of recombination, production of genomic re-arrangements, and production of pseudogenization following insertion into gene coding sequences [30]. Therefore, regions replete with these elements may experience enhanced levels of gene silencing through one or more of the processes involved with transposition.

Transposons are also non-randomly arrayed within larger chromosomal regions that may share fairly similar recombination levels as it has been shown that large transposon-free regions (TFR) spanning several kb often surround important regulatory gene regions in the genome such as early development regulating genes [31,32]. Strong constraints likely exist on silencing of important early development genes thus purging trasnspositional inserts from these locations. These regions do not appear to be localized in particular lower frequency transposon regions of the genome, as the occurrence of transposable elements in the vicinities surrounding these transposon-free regions may be quite high, and they have been observed to be scattered throughout the length of chromosomes [31,32]. However, for the two-classes of TFR that have been categorized (i.e., high and low GC content TFR), it has been reported that high GC content TFR appear to localize more towards the telomeric regions of human chromosomes [31], which may in turn suggest that these regions may be surrounded with a higher than average density of transposable elements in heterochromatin tracts.

To investigate if the comparative homology regions within medaka or zebrafish may possess differential distributions of the most common transposon (i.e., Tc1/mariner) families in salmonids, a BLASTN search was performed using the recently documented sequences from several family members in this grouping from Atlantic salmon [33] and rainbow trout NCBI accessions (see Additional Files 16, 17). The ENSEMBL 'distant homologies' default configuration was used, with an arbitrary acceptance cut-off e-value of 10^-6^, and bp length of ≥ 100. The first 50 highest ranked fragments meeting or exceeding these criteria, for each family member were retained for the analysis. While the results for zebrafish were mixed with respect to the distribution of Tc1 elements across chromosomes, within the medaka genome, there was evidence that chromosomes belonging to ancestral groupings B, C, D, E, and K, had somewhat lower distributions of Tc1-like fragments scattered throughout their length (see Additional File 18). The distribution of Tc1-element hits to the medaka genome also paralleled the distribution of DNA family, SINE, LINE and LTR transposable elements identified within medaka chromosomes [34] given that the number of salmonid Tc1 elements assigned to a given medaka chromosome were inversely related to the average distance (bp) between medaka transposable element sites within each chromosome (see Additional File 19). In other words, medaka chromosomes that possessed a higher density of transposable elements also received a higher frequency of salmonid Tc1 element hits. Whether regions retaining higher levels of duplicate gene expression do in fact possess lower levels of interspaced transpositional elements within the salmonid genome will be an area of future research interest.

## Conclusion

Salmonids, due to their 4R polyploid ancestry retain the genetic signatures of past gene duplication events. Most extant duplicated DNA copies map to the chromosome arms that are immediate descendants of the 4R duplication. However, up to 4 different chromosomal arms in their genome can be related through comparative synteny searches, to two different chromosome arms in model teleost fishes such as zebrafish and medaka (i.e., representatives of the 3R WGD). Based upon models of vertebrate chromosome evolution [10,20], we may infer that two sets of the ancestral teleost-specific linkage groups may actually have undergone a triplication (i.e., doubling of one of the two chromosomes in the linkage group set) prior to radiation in the soft-rayed fishes. The distribution of duplicated genes in the genomes of rainbow trout and Atlantic salmon is mosaic, and it is postulated that their retention will be negatively correlated with distribution of transposable elements within the genomes of these fishes.

## Methods

Descriptions and characteristics of the two rainbow trout mapping panels, and two Atlantic salmon mapping panels used for the comparative studies have previously been provided [23]. Genetic map construction and comparative analyses among maps were performed using various modules within the software package LINKMFEX [35]. Synteny blocks were defined as regions containing two or more adjacent markers from a comparison species aligning within the same linkage group or chromosomal region within the source species. In instances where a single marker from an unrelated linkage group in the comparison species was located within the synteny block, the region was still considered to be uninterrupted. However, localization of two or more unrelated linkage group markers within the region denoted a discontinuity. Synteny block assignments were assessed using BLOCKON within the LINKMFEX package. All graphical depictions of linkage group affinities were constructed using MAPCHART [36].

Primer sequences for the rainbow trout and Atlantic salmon type I markers, SSR and EST markers can be found at the cGRASP websites:  and  with BAC end primer sequence information located exclusively at the former website. Information on the majority of the markers used in this study can also be obtained directly from the NCBI website . All SNP genotyping was performed using the MassARRAY system from Sequenom (San Diego, CA, USA), and details on the protocol, EST sequences, and SNP polymorphisms are given in Moen et al. [19].

### Comparative analyses

Homology assignments to the 3R teleost genomes were performed using the Distant Homologies BLASTN default options in ENSEMBL  v47 – v49. The most recent assemblies for the zebrafish genome (Zv7, July 2007) and medaka (MEDAKA1, October 2005) were the subjects of query searches using the various type I, SSR, EST, and BAC end sequences from the salmonids. Sequences having expectation values lower than 10^-6 ^and identity values greater than 70% were accepted as possessing acceptable homology matches to the salmonid genomes. In instances where multiple matches to several different linkage groups were detected, all with relatively low e-values, the marker assignments were ignored. In many instances, however, matches to only 1 or a few alternate linkage groups were evident in the subject searches. If the identified linkage groups were consistent with expected duplicated 3R linkage group affinities, the marker assignments were reported in Additional Files 13 and 14. EST and type I gene markers that did not find an acceptable homology match following BLASTN searches were further queried to subject model genome databases using TBLASTX. Criteria similar to the BLASTN searches were adopted. Matches obtained with this search parameter were, however, in many instances non-confirmatory (i.e., homology assignments for the query sequence would often be made to a linkage group other than the linkage group composing the surrounding synteny block assignment for multiple additional BLASTN assigned markers). This is most likely due to the fact that many of the query genes belong to gene families possessing many multiple closely related gene copies. Hence assignments to alternate linkage groups may be an expectation when using translated amino acid string queries.

Information on the assignment of ancestral proto-Actinopterygian linkage groups within the genomes of zebrafish and medaka were obtained from the Kasahara et al. study [20]. Thirteen putative ancestral linkage groups were postulated to have given rise all the extant teleost genome structures, with the 13^th ^linkage group (M) present in triplicate (following a single chromosome duplication) shortly following the origin of the ray-finned fishes. Each ancestral grouping should exhibit extensive homology to a minimum of two linkage groups within extant 3R derivative teleosts. Due to rearrangements within the genomes of these species, multiple smaller linkage group affinities are also evident for certain ancestral groups. The available data summarized in Table 1 shows the affinities of these ancestral groupings within zebrafish and medaka chromosomes. Doubly conserved synteny blocks with the 3R species were used to infer homology blocks to the ancestral chromosomes using either rainbow trout or Atlantic salmon as the reference species. Cross-referencing of the reciprocal salmonid linkage group homology blocks were also used to help reciprocally verify the ancestral assignments (see Table 1, Figure 1, Additional File 13, and Additional File 14). In other words, if an ancestral assignment to a particular rainbow trout linkage group arm was given, and this arm demonstrated extensive homology to an Atlantic salmon arm segment, the assignment of the Atlantic salmon arm segment to the same ancestral grouping was verified.

### Transposon analysis

Data for the salmonid Tc1/Mariner elements were obtained from the de Boer et al. study [33] along with sequence information downloaded from the NCBI website using key word searches filtered according to salmonid-specific sequences. Repeat Masker [37] data files specific for a search within each medaka chromosome were downloaded from the UCSC website [34]. The number of hits reported within each chromosome was simply added across all annotation classes of DNA, LINE, SINE and LTR. The total sequence length of each medaka chromosome was then divided by the total number of transposable element assignments to obtain an overall chromosome estimate of the average bp interval between site insertions.

### Designation of chromosome arms

Within metacentric chromosomes we adopt the notation that short arms are designated as 'p' arms, while the long arm within each chromosome is designated as the 'q' arm. For fused Atlantic salmon chromosome arms, the segment closest to the centromere is designated as 'a' and more distal segments are designated as 'b' and 'c' [21]. These occur within the long arm of each linkage group and thus the segments are designated qa, qb, and in one instance qc.

## Authors' contributions

RGD carried out the bioinformatics analysis and wrote the manuscript, and along with MMF and WSD helped organize the conceptual framework of the manuscript. EAD, KG, KL, HKM and JP helped with development and genotyping of EST and BAC-end sequence markers, along with BH for many of the SSR markers. RBP helped with the interpretation of chromosome arm assignments related to short/long arm designations. BFK and SL contributed information on the Atlantic salmon SNP markers. All authors read and commented on the manuscript.

## Supplementary Material

Additional File 1 **s_table1_rt_lgs.xls.** Genetic marker assignments to rainbow trout linkage groups. Click here for file

Additional File 2 **s_table2_as_lgs.xls**. Genetic marker assignments to Atlantic salmon linkage groups. Click here for file

Additional File 3 **s_table3_l25f.map**. Female genetic map for rainbow trout Lot 25 mapping panel. Click here for file

Additional File 4 **s_table4_l44f.map.** Female genetic map for rainbow trout Lot 44 mapping panel. Click here for file

Additional File 5 **s_table5_l25m.map.** Male genetic map for rainbow trout Lot 25 mapping panel. Click here for file

Additional File 6 **s_table6_l44m.map**. Male genetic map for rainbow trout Lot 44 mapping panel. Click here for file

Additional File 7 **s_table7_br5f.map**. Female genetic map for Atlantic salmon Br5 mapping panel. Click here for file

Additional File 8 **s_table8_br6f.map.** Female genetic map for Atlantic salmon Br6 mapping panel. Click here for file

Additional File 9 **s_table9_br5m.map.** Male genetic map for Atlantic salmon Br5 mapping panel. Click here for file

Additional File 10 **s_table10_br6m.map.** Male genetic map for Atlantic salmon Br6 mapping panel. Click here for file

Additional File 11 **s_figure1-rtvsas_homology.pdf**. Depiction of homologous Atlantic salmon linkage group blocks within the rainbow trout genome. Merged rainbow trout female maps are used as a template. Click here for file

Additional File 12 **s_figure2-asvsrt_homology.pdf**. Depiction of homologous rainbow trout linkage group blocks within the Atlantic salmon genome. Merged Atlantic salmon female maps are used as a template. Click here for file

Additional File 13 **s_table11_rt-vs-dr&olat_synteny.xls.** Database of zebrafish and medaka synteny blocks within the rainbow trout genome. Click here for file

Additional File 14 **s_table12_as-vs-dr&olat_synteny.xls.** Database of zebrafish and medaka synteny blocks within the Atlantic salmon genome. Click here for file

Additional File 15 **supplementary figure 3.ppt.** Regions of shared homology among zebrafish chromosomes 5, 10 and 21, and their shared affinities with zebrafish chromosome 8. Click here for file

Additional File 16 **s_table13_tc1_transposon-hits-medaka.xls**. Location of salmonid mariner/Tc1 elements in the medaka genome. Click here for file

Additional File 17 **s_table14_salmonid_transposon-hits-vs-zf&medaka.xls**. Counts of Tc1 element salmonid transposon BLASTN hits to all zebrafish and medaka linkage groups. Click here for file

Additional File 18 **supplementary figure 4.ppt**. Number of Tc1/mariner transposon hits to zebrafish and medaka linkage groups. Click here for file

Additional File 19 **supplementary figure 5.ppt**. Average bp interval among all DNA/LINE/SINE/LTR transposons detected among medaka linkage groups. Click here for file

## References

[B1] Allendorf FW, Thorgaard GH, Turner BJ (1984). Tetraploidy and the evolution of salmonid fishes. Evolutionary Genetics of Fishes.

[B2] Sakamoto T, Danzmann RG, Gharbi K, Howard P, Ozaki A, Khoo SK, Woram RA, Okamoto N, Ferguson MM (2000). A microsatellite linkage map of rainbow trout (*Oncorhynchus mykiss*) characterized by large sex-specific differences in recombination rates. Genetics.

[B3] Nichols KM, Young WP, Danzmann RG, Robison BD, Rexroad C, Noakes M, Phillips RB, Bentzen P, Spies I, Knudsen K (2003). A consolidated linkage map for rainbow trout (*Oncorhynchus mykiss*). Anim Genet.

[B4] Gharbi K, Gautier A, Danzmann RG, Gharbi S, Sakamoto T, Hoyheim B, Taggart JB, Cairney M, Powell R, Kreig F (2006). A linkage map for brown trout (*Salmo trutta*): Chromosome homeologies and comparative genome organization with other salmonid fish. Genetics.

[B5] Wright JE, Johnson K, Hollister A, May B (1983). Meiotic models to explain classical linkage, pseudolinkage, and chromosome paring in tetraploid derivative salmonid genomes. Isozymes Curr Top Biol Med Res.

[B6] Allendorf FW, Danzmann RG (1997). Secondary tetrasomic segregation of MDH-B and preferential pairing of homeologues in rainbow trout. Genetics.

[B7] Phillips RB, Rab P (2001). Chromosome evolution in the Salmonidae (Pisces): an update. Biol Rev.

[B8] Mank JE, Avise JC (2006). Phylogenetic conservation of chromosome number in Actinopterygian fishes. Genetica.

[B9] Jaillon O, Aury JM, Brunet F, Petit JL, Stange-Thomann N, Mauceli E, Bouneau L, Fischer C, Ozouf-Costaz C, Bernot  A (2004). Genome duplication in the teleost fish *Tetraodon nigroviridis *reveals the early vertebrate proto-karyotype. Nature.

[B10] Nakatani Y, Takeda H, Kohara Y, Morishita S (2007). Reconstruction of the vertebrate ancestral genome reveals dynamic genome reorganization in early vertebrates. Genome Res.

[B11] Panopoulou G, Poustka AJ (2005). Timing and mechanism of ancient vertebrate genome duplications – the adventure of a hypothesis. Trends Genet.

[B12] Vandepoele K, De Vos W, Taylor JS, Meyer A, Peer Y Van de (2004). Major events in the genome evolution of vertebrates: Panome age and size differ considerably between ray-finned fishes and land vertebrates. Proc Natl Acad Sci.

[B13] Meyer A, Peer Y Van de (2005). From 2R to 3R: evidence for a fish-specific genome duplication (FSGD). Bioessays.

[B14] Lappin TRJ, Grier DG, Thompson A, Halliday HL (2006). Hox genes: Seductive science, mysterious mechanisms. Ulster Med J.

[B15] Hoegg S, Meyer A (2005). Hox clusters as models for vertebrate genome evolution. Trends Genet.

[B16] Moghadam HK, Ferguson MM, Danzmann RG (2005). Evidence for Hox gene duplications in rainbow trout (*Oncorhynchus mykiss*): A tetraploid model species. J Mol Evol.

[B17] Moghadam HK, Ferguson MM, Danzmann RG (2005). Evolution of Hox clusters in Salmonidae: A comparative analysis between Atlantic salmon (*Salmo salar*) and rainbow trout (*Oncorhynchus mykiss*). J Mol Evol.

[B18] Moen T, Hoyheim B, Munck H, Gomez-Raya L (2004). A linkage map of Atlantic salmon (Salmo salar) reveals an uncommonly large difference in recombination rate between the sexes. Animal Genet.

[B19] Moen T, Hayes B, Baranski M, Berg PR, Kjoglum S, Koop BF, Davidson WS, Omholt SW, Lien S (2008). A linkage map of the Atlantic salmon (Salmo salar) based on EST-derived SNP markers. BMC Genomics.

[B20] Kasahara M, Naruse K, Sasaki S, Nakatani Y, Qu W, Ahsan B, Yamada T, Nagayasu Y, Doi K, Kasai  Y (2007). The medaka draft genome and insights into vertebrate genome evolution. Nature.

[B21] Phillips RB

[B22] Phillips RB, Nichols KM, DeKoning JJ, Morasch MR, Keatley KA, Rexroad C, Ghar SA, Danzmann RG, Drew RE, Thorgaard  GH (2006). Assignment of rainbow trout linkage groups to specific chromosomes. Genetics.

[B23] Danzmann RG, Cairney M, Davidson WS, Ferguson MM, Gharbi K, Guyomard R, Holm L-E, Leder E, Okamoto N, Ozaki  A (2005). A comparative analysis of the rainbow trout genome with 2 other species of fish (Arctic char and Atlantic salmon) within the tetraploid derivative Salmonidae family (subfamily: Salmoninae). Genome.

[B24] Myers S, Spencer CCA, Auton A, Bottolo L, Freeman C, Donnelly P, McVean G (2006). The distribution and causes of meiotic recombination in the human genome. Biochem Soc Trans.

[B25] Kochakpour N, Moens PB (2008). Sex-specific crossover patterns in zebrafish (*Danio rerio*). Heredity.

[B26] Woram RA, McGowan C, Stout JA, Gharbi K, Ferguson MM, Hoyheim B, Davidson EA, Davidson WS, Rexroad C, Danzmann RG (2004). A genetic linkage map for Arctic charr (*Salvelinus alpinus*): evidence for higher recombination rates and segregation distortion in hybrid versus pure strain mapping parents. Genome.

[B27] Rizzon C, Marais G, Gouy M, Biemont C (2002). Recombination rate and the distribution of transposable elements in the Drosophila melanogaster genome. Genome Res.

[B28] Bartolome C, Maside X, Charlesworth B (2002). On the abundance and distribution of transposable elements in the genome of *Drosophila melanogaster*. Mol Biol Evol.

[B29] Schwarzacher T (2003). Meiosis, recombination and chromosomes: a review of gene isolation and fluorescent *in situ *hybridization data in plants. J Exp Botany.

[B30] Biemont C, Vieira C (2005). What transposable elements tell us about genome organization and evolution: the case of *Drosophila*. Cytogenet Genome Res.

[B31] Simons C, Pheasant M, Makunin IV, Mattick JS (2005). Transposon-free regions in mammalian genomes. Genome Res.

[B32] Simons C, Makunin IV, Pheasant M, Mattick JS (2007). Maintenance of transposon-free regions throughout vertebrate evolution. BMC Genomics.

[B33] de Boer JG, Yazawa R, Davidson WS, Koop BF (2007). Bursts of horizontal evolution of DNA transposons in the speciation of pseudotetraploid salmonids. BMC Genomics.

[B34] UCSC Genome Browser. http://hgdowload.cse.ucsc.edu/downloads.html.

[B35] Danzmann RG, Gharbi K (2001). Gene mapping in fishes: a means to an end. Genetica.

[B36] Voorrips RE (2002). Mapchart: software for the graphical presentation of linkage maps and QTLs. J Hered.

[B37] Repeatmasker. http://www.repeatmasker.org.

[B38] Guyomard R, Mauger S, Tabet-Canale K, Martineau S, Genet C, Kreig, Quillet E (2006). A type I and type II microsatellite linkage map of rainbow trout (*Oncorhynchus mykiss*) with presumptive coverage of all chromosome arms. BMC Genomics.

