# Supplementary material for: Distribution of ancestral proto-Actinopterygian chromosome arms within the genomes of 4R-derivative salmonid fishes (Rainbow trout and Atlantic salmon)
Source: BMC Genomics. 2008 Nov 25;9:557. doi: 10.1186/1471-2164-9-557 (PMC2632648; doi:10.1186/1471-2164-9-557)
Supplement: Additional File 11 — s_figure1-rtvsas_homology.pdf. Depiction of homologous Atlantic salmon linkage group blocks within the rainbow trout genome. Merged rainbow trout female maps are used as a template. [file 1471-2164-9-557-S11.pdf]

Supplementary Figure 1: Rainbow trout merged female genetic map depicting all linkage groups numbered in sequence. The 'f' suffix following the linkage group name indicates it is a female map. Map distances are indicated as observed recombination distances (Θ) where 1% recombination = 1 cM. Homologous regions within rainbow trout to their respective Atlantic salmon linkage groups are indicated to the right of each linkage group. Rainbow trout chromosomes ranked according to size (Phillips et al. 2006) are indicated below each linkage group designation.

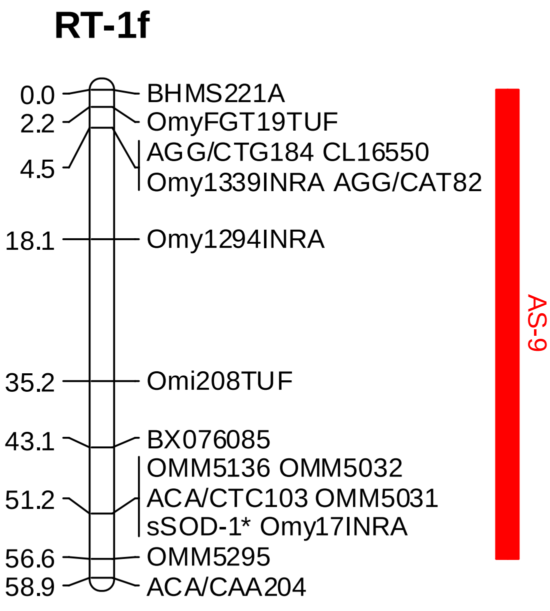

Chromosome *Sex*

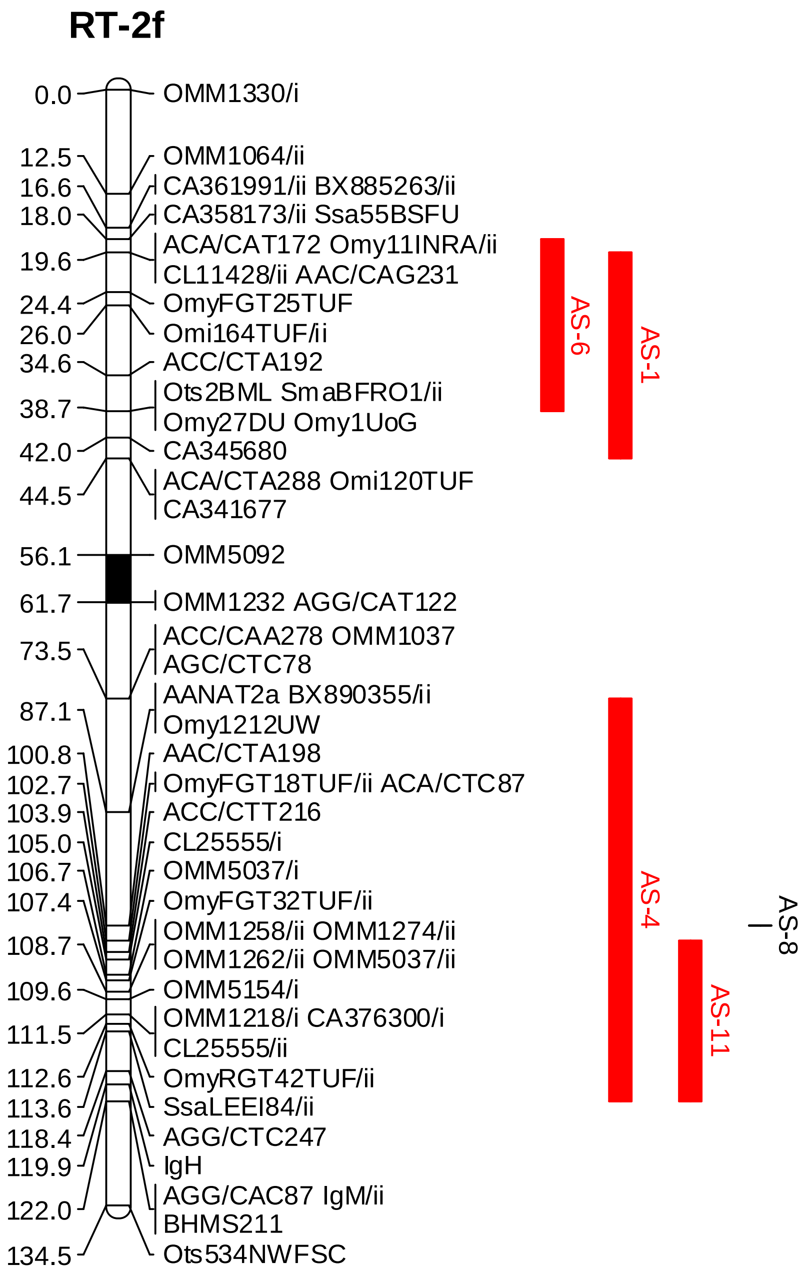

Chromosome 13

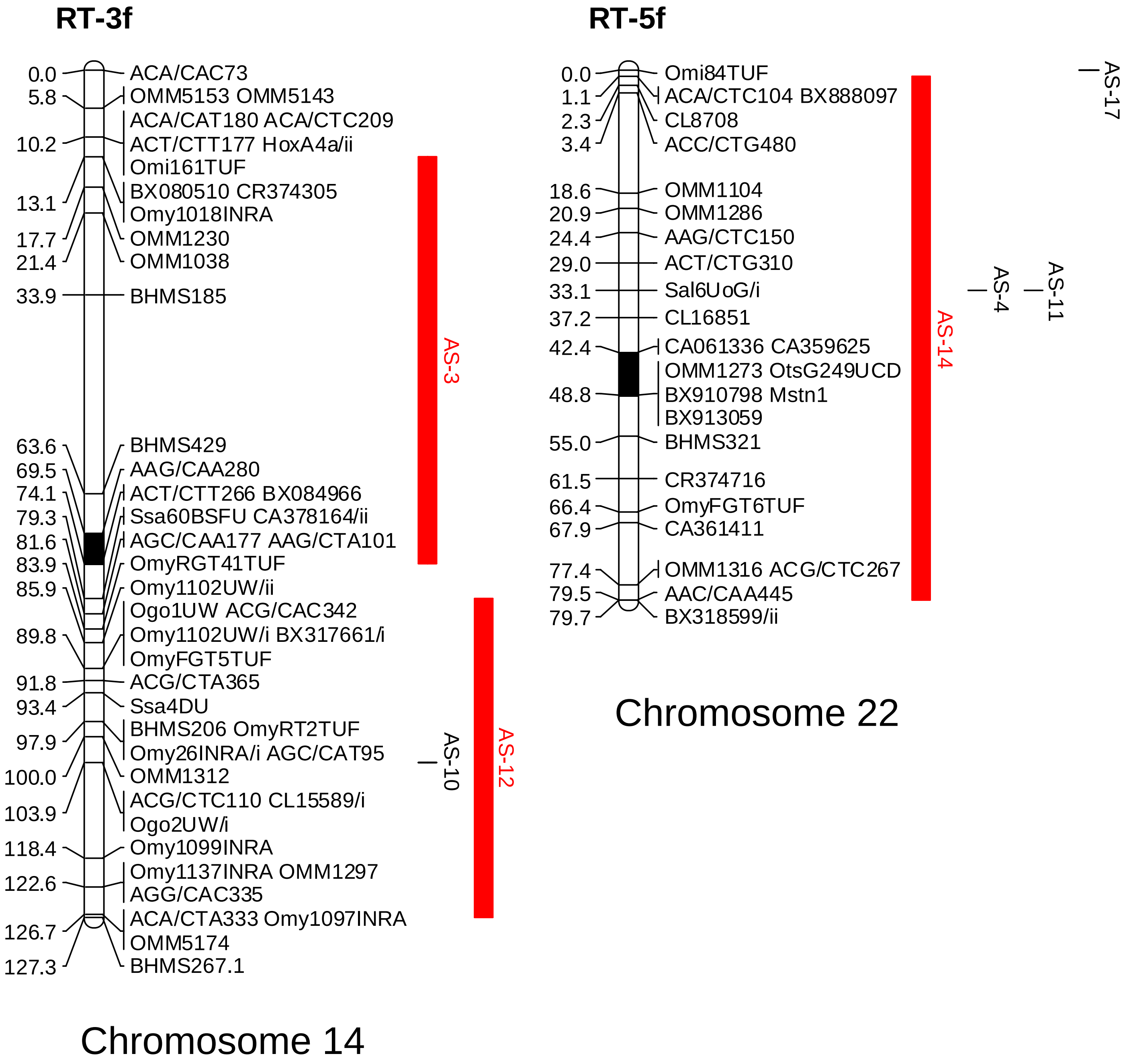
RT-3f
0.0 ACA/CAC73
5.8 OMM5153 OMM5143
ACA/CAT180 ACA/CTC209
10.2 ACT/CTT177 HoxA4a/ii
Omi161TUF
13.1 BX080510 CR374305
Omy1018INRA
17.7 OMM1230
21.4 OMM1038
33.9 BHMS185
63.6 BHMS429
69.5 AAG/CAA280
74.1 ACT/CTT266 BX084966
79.3 Ssa60BSFU CA378164/ii
81.6 AGC/CAA177 AAG/CTA101
83.9 OmyRGT41TUF
85.9 Omy1102UW/ii
Ogo1UW ACG/CAC342
89.8 Omy1102UW/i BX317661/i
OmyFGT5TUF
91.8 ACG/CTA365
93.4 Ssa4DU
BHMS206 OmyRT2TUF
97.9 Omy26INRA/i AGC/CAT95
100.0 OMM1312
ACG/CTC110 CL15589/i
103.9 Ogo2UW/i
118.4 Omy1099INRA
122.6 Omy1137INRA OMM1297
AGG/CAC335
126.7 ACA/CTA333 Omy1097INRA
OMM5174
127.3 BHMS267.1
AS-3
AS-10
AS-12
Chromosome 14
RT-5f
0.0 Omi84TUF
1.1 ACA/CTC104 BX888097
2.3 CL8708
3.4 ACC/CTG480
18.6 OMM1104
20.9 OMM1286
24.4 AAG/CTC150
29.0 ACT/CTG310
33.1 Sal6UoG/i
37.2 CL16851
42.4 CA061336 CA359625
OMM1273 OtsG249UCD
48.8 BX910798 Mstn1
BX913059
55.0 BHMS321
61.5 CR374716
66.4 OmyFGT6TUF
67.9 CA361411
77.4 OMM1316 ACG/CTC267
79.5 AAC/CAA445
79.7 BX318599/ii
AS-14
AS-4
AS-11
AS-17
Chromosome 22

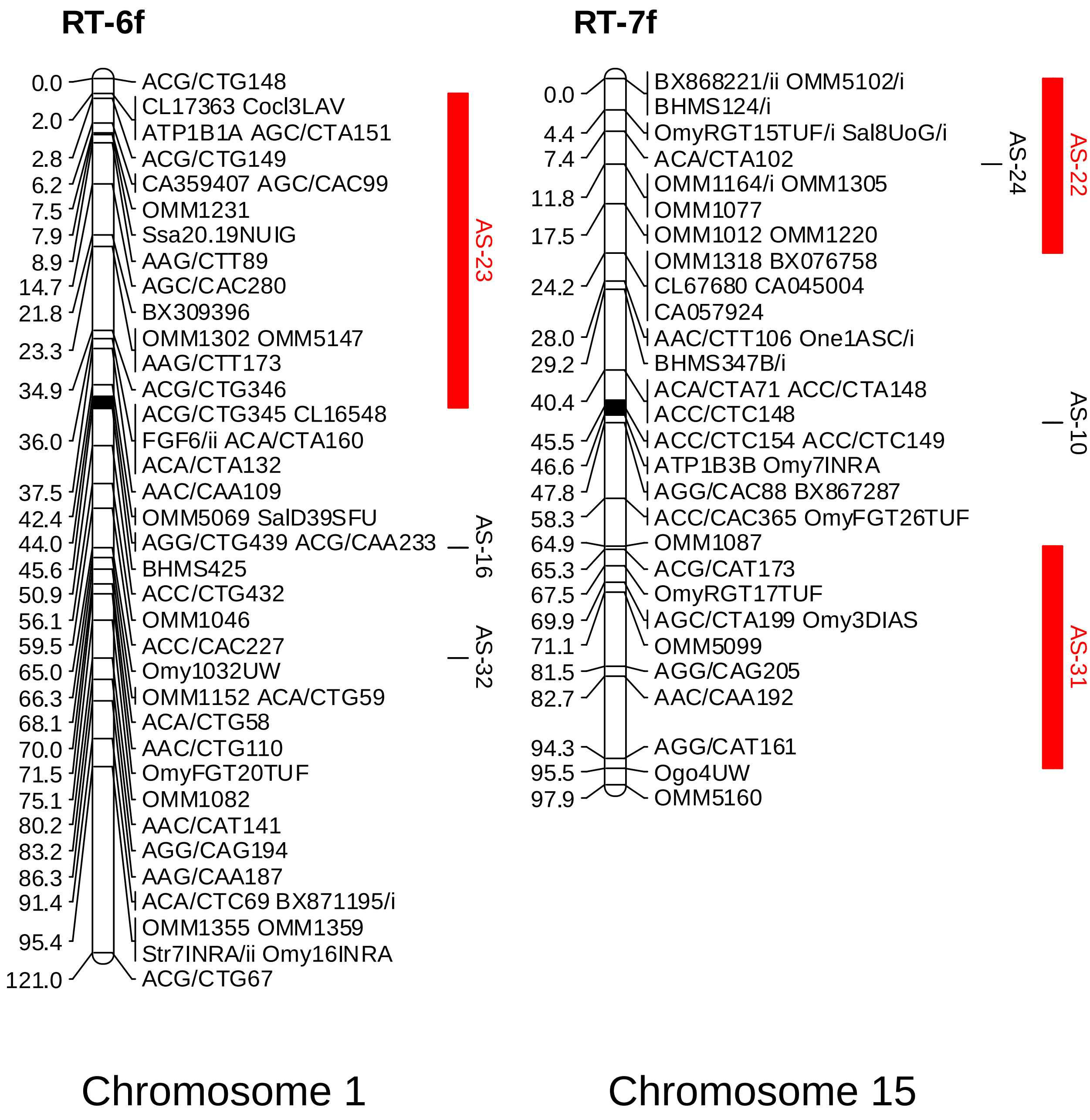
RT-6f
0.0
2.0
2.8
6.2
7.5
7.9
8.9
14.7
21.8
23.3
34.9
36.0
37.5
42.4
44.0
45.6
50.9
56.1
59.5
65.0
66.3
68.1
70.0
71.5
75.1
80.2
83.2
86.3
91.4
95.4
121.0
ACG/CTG148
CL17363 Cocl3LAV
ATP1B1A AGC/CTA151
ACG/CTG149
CA359407 AGC/CAC99
OMM1231
Ssa20.19NUIG
AAG/CTT89
AGC/CAC280
BX309396
OMM1302 OMM5147
AAG/CTT173
ACG/CTG346
ACG/CTG345 CL16548
FGF6/ii ACA/CTA160
ACA/CTA132
AAC/CAA109
OMM5069 SalD39SFU
AGG/CTG439 ACG/CAA233
BHMS425
ACC/CTG432
OMM1046
ACC/CAC227
Omy1032UW
OMM1152 ACA/CTG59
ACA/CTG58
AAC/CTG110
OmyFGT20TUF
OMM1082
AAC/CAT141
AGG/CAG194
AAG/CAA187
ACA/CTC69 BX871195/i
OMM1355 OMM1359
Str7INRA/ii Omy16INRA
ACG/CTG67
AS-23
AS-16
AS-32
Chromosome 1
RT-7f
0.0
4.4
7.4
11.8
17.5
24.2
28.0
29.2
40.4
45.5
46.6
47.8
58.3
64.9
65.3
67.5
69.9
71.1
81.5
82.7
94.3
95.5
97.9
BX868221/ii OMM5102/i
BHMS124/i
OmyRGT15TUF/i Sal8UoG/i
ACA/CTA102
OMM1164/i OMM1305
OMM1077
OMM1012 OMM1220
OMM1318 BX076758
CL67680 CA045004
CA057924
AAC/CTT106 One1ASC/i
BHMS347B/i
ACA/CTA71 ACC/CTA148
ACC/CTC148
ACC/CTC154 ACC/CTC149
ATP1B3B Omy7INRA
AGG/CAC88 BX867287
ACC/CAC365 OmyFGT26TUF
OMM1087
ACG/CAT173
OmyRGT17TUF
AGC/CTA199 Omy3DIAS
OMM5099
AGG/CAG205
AAC/CAA192
AGG/CAT161
Ogo4UW
OMM5160
AS-24
AS-22
AS-10
AS-31
Chromosome 15

RT-8f

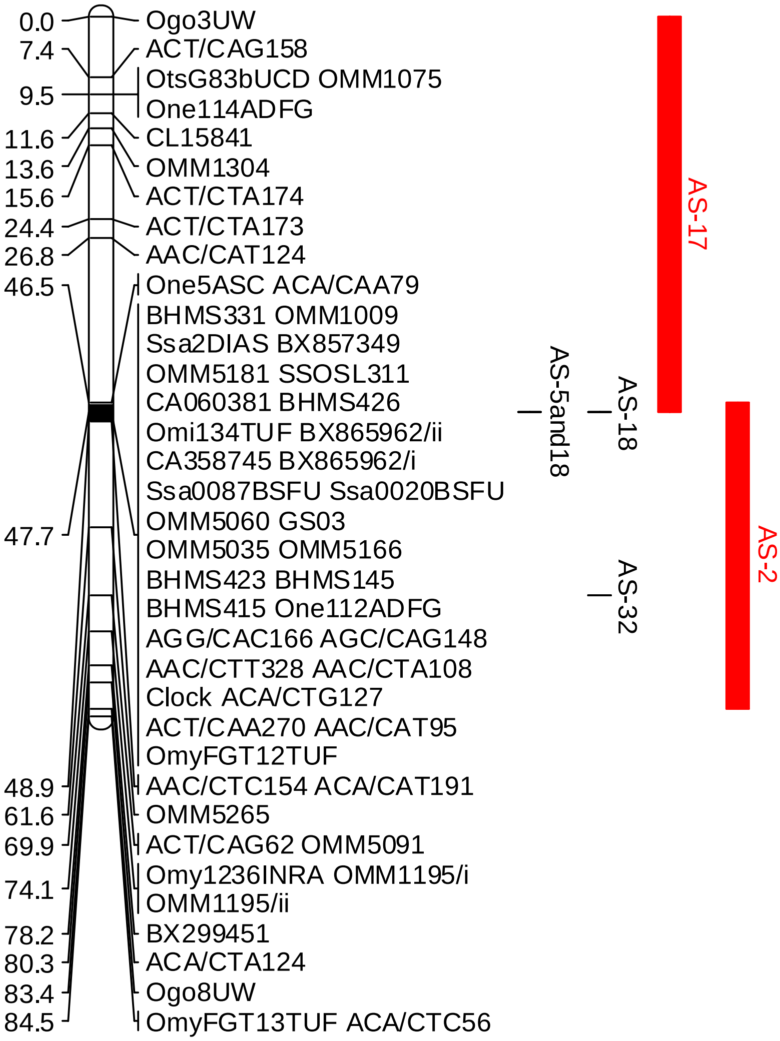

Chromosome 5

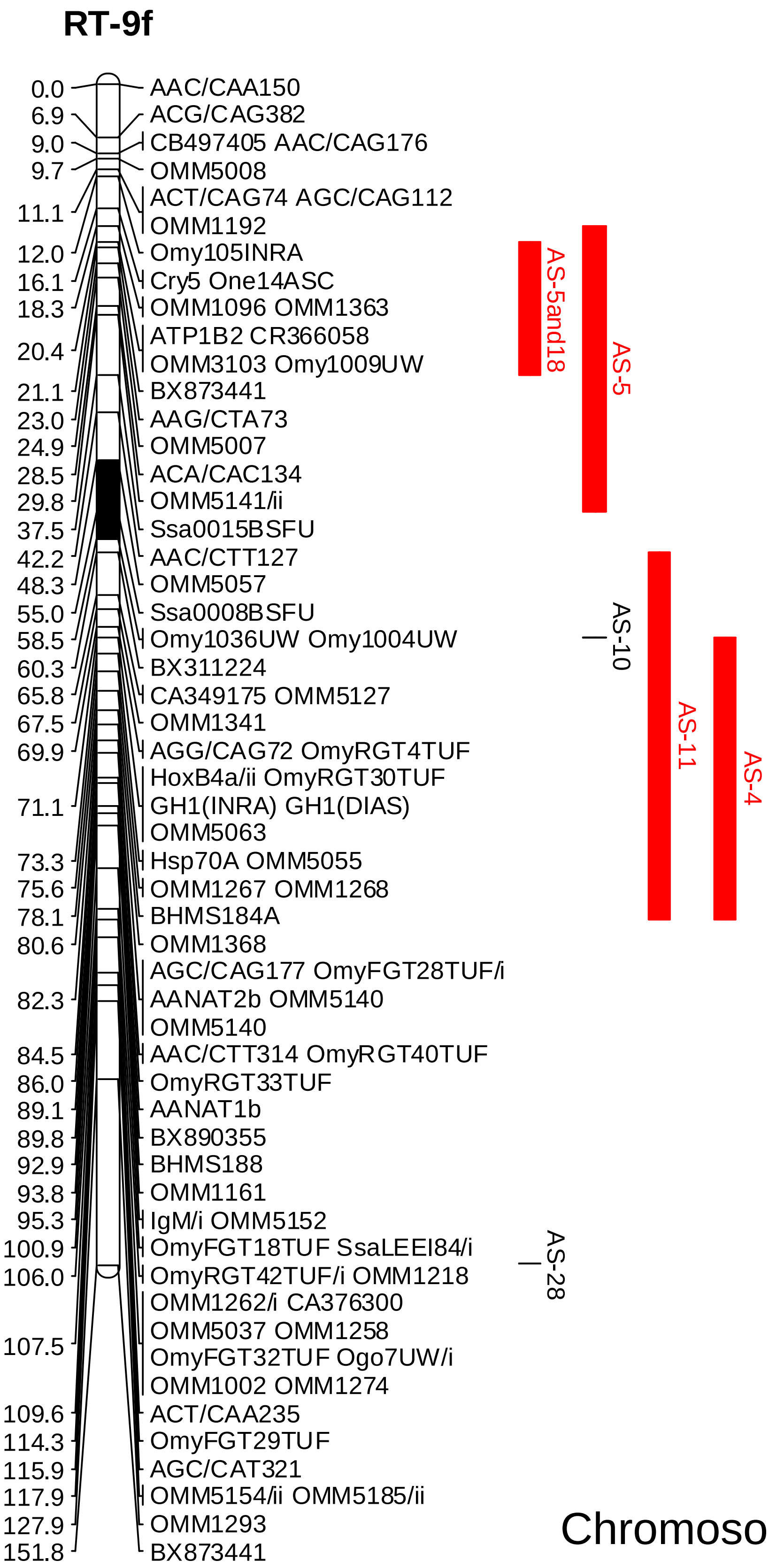

Chromosome 12

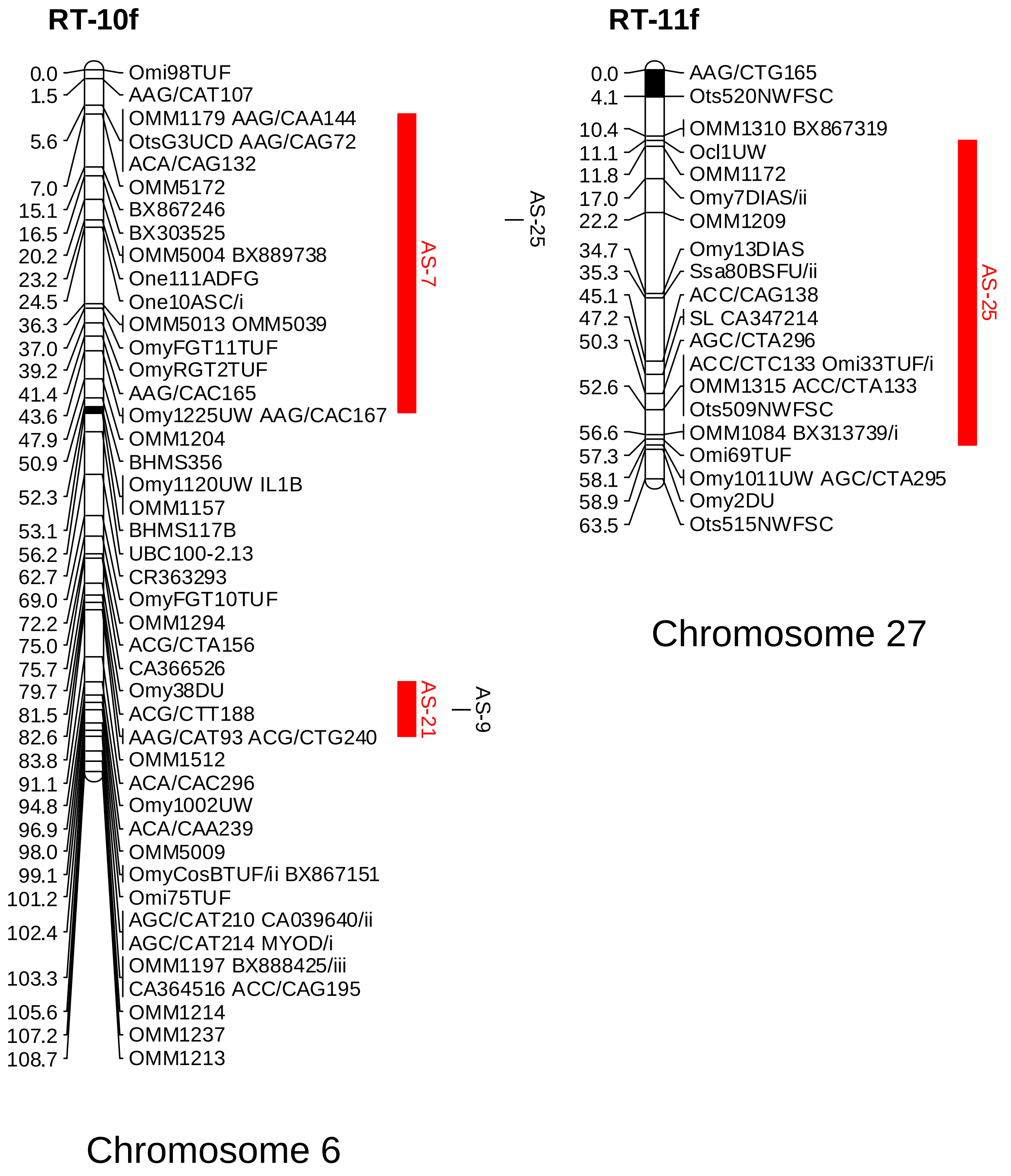
RT-10f
0.0 Omi98TUF
1.5 AAG/CAT107
5.6 OMM1179 AAG/CAA144
OtsG3UCD AAG/CAG72
ACA/CAG132
7.0 OMM5172
15.1 BX867246
16.5 BX303525
20.2 OMM5004 BX889738
23.2 One111ADFG
24.5 One10ASC/i
36.3 OMM5013 OMM5039
37.0 OmyFGT11TUF
39.2 OmyRGT2TUF
41.4 AAG/CAC165
43.6 Omy1225UW AAG/CAC167
47.9 OMM1204
50.9 BHMS356
52.3 Omy1120UW IL1B
OMM1157
53.1 BHMS117B
56.2 UBC100-2.13
62.7 CR363293
69.0 OmyFGT10TUF
72.2 OMM1294
75.0 ACG/CTA156
75.7 CA366526
79.7 Omy38DU
81.5 ACG/CTT188
82.6 AAG/CAT93 ACG/CTG240
83.8 OMM1512
91.1 ACA/CAC296
94.8 Omy1002UW
96.9 ACA/CAA239
98.0 OMM5009
99.1 OmyCosBTUF/ii BX867151
101.2 Omi75TUF
102.4 AGC/CAT210 CA039640/ii
AGC/CAT214 MYOD/i
103.3 OMM1197 BX888425/iii
CA364516 ACC/CAG195
105.6 OMM1214
107.2 OMM1237
108.7 OMM1213
AS-7
AS-25
AS-21
AS-9
Chromosome 6
RT-11f
0.0 AAG/CTG165
4.1 Ots520NWFSC
10.4 OMM1310 BX867319
11.1 Ocl1UW
11.8 OMM1172
17.0 Omy7DIAS/ii
22.2 OMM1209
34.7 Omy13DIAS
35.3 Ssa80BSFU/ii
45.1 ACC/CAG138
47.2 SL CA347214
50.3 AGC/CTA296
52.6 ACC/CTC133 Omi33TUF/i
OMM1315 ACC/CTA133
Ots509NWFSC
56.6 OMM1084 BX313739/i
57.3 Omi69TUF
58.1 Omy1011UW AGC/CTA295
58.9 Omy2DU
63.5 Ots515NWFSC
AS-25
Chromosome 27

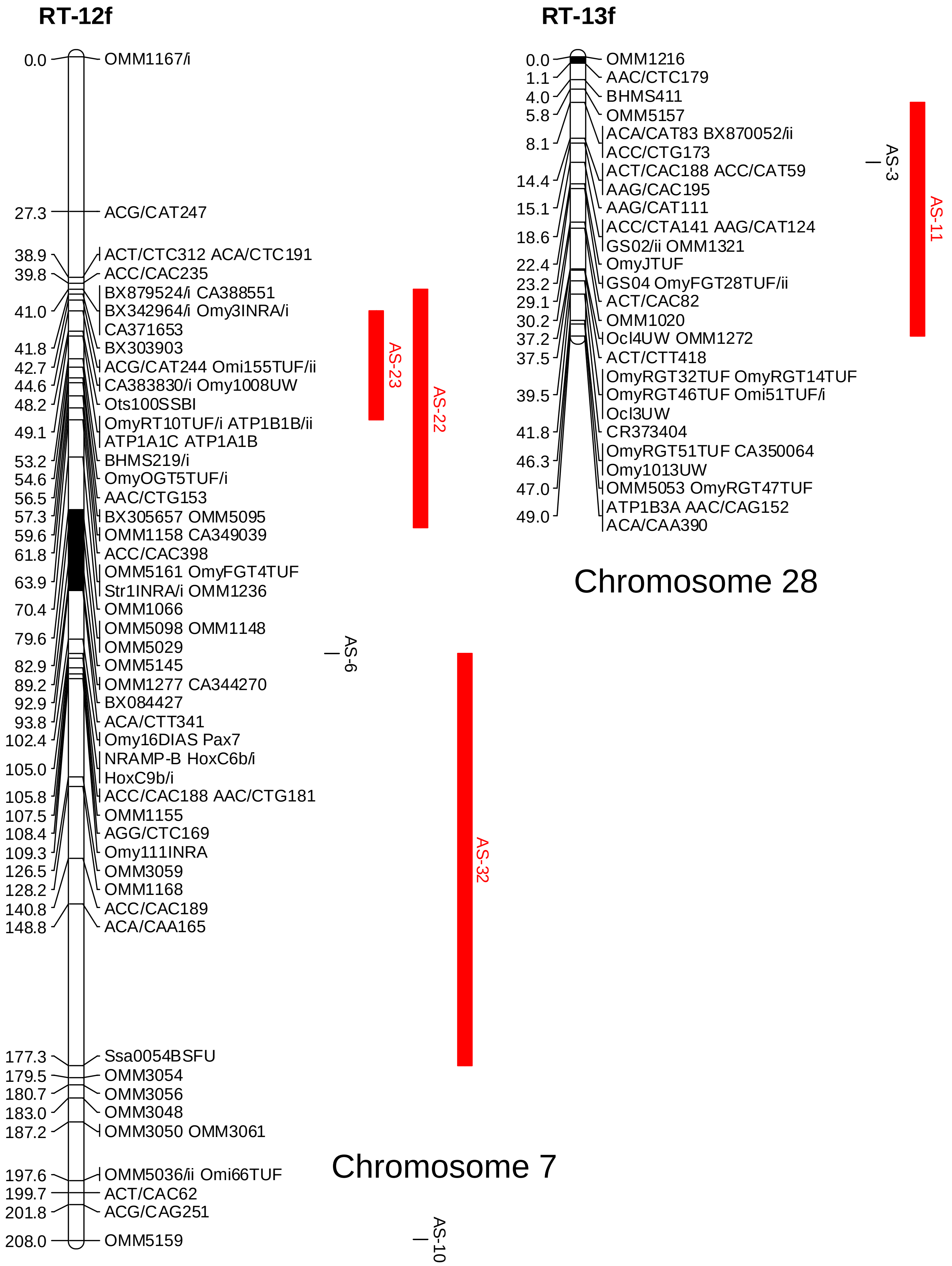
RT-12f
0.0 OMM1167/i
27.3 ACG/CAT247
38.9 ACT/CTC312 ACA/CTC191
39.8 ACC/CAC235
41.0 BX879524/i CA388551
BX342964/i Omy3INRA/i
CA371653
41.8 BX303903
42.7 ACG/CAT244 Omi155TUF/ii
44.6 CA383830/i Omy1008UW
48.2 Ots100SSBI
49.1 OmyRT10TUF/i ATP1B1B/ii
ATP1A1C ATP1A1B
53.2 BHMS219/i
54.6 OmyOGT5TUF/i
56.5 AAC/CTG153
57.3 BX305657 OMM5095
59.6 OMM1158 CA349039
61.8 ACC/CAC398
63.9 OMM5161 OmyFGT4TUF
Str1INRA/i OMM1236
70.4 OMM1066
79.6 OMM5098 OMM1148
OMM5029
82.9 OMM5145
89.2 OMM1277 CA344270
92.9 BX084427
93.8 ACA/CTT341
102.4 Omy16DIAS Pax7
105.0 NRAMP-B HoxC6b/i
HoxC9b/i
105.8 ACC/CAC188 AAC/CTG181
107.5 OMM1155
108.4 AGG/CTC169
109.3 Omy111INRA
126.5 OMM3059
128.2 OMM1168
140.8 ACC/CAC189
148.8 ACA/CAA165
177.3 Ssa0054BSFU
179.5 OMM3054
180.7 OMM3056
183.0 OMM3048
187.2 OMM3050 OMM3061
197.6 OMM5036/ii Omi66TUF
199.7 ACT/CAC62
201.8 ACG/CAG251
208.0 OMM5159
AS-23
AS-22
AS-6
AS-32
AS-10
Chromosome 7
RT-13f
0.0 OMM1216
1.1 AAC/CTC179
4.0 BHMS411
5.8 OMM5157
8.1 ACA/CAT83 BX870052/ii
ACC/CTG173
14.4 ACT/CAC188 ACC/CAT59
AAG/CAC195
15.1 AAG/CAT111
18.6 ACC/CTA141 AAG/CAT124
GS02/ii OMM1321
22.4 OmyJTUF
23.2 GS04 OmyFGT28TUF/ii
29.1 ACT/CAC82
30.2 OMM1020
37.2 Ocl4UW OMM1272
37.5 ACT/CTT418
39.5 OmyRGT32TUF OmyRGT14TUF
OmyRGT46TUF Omi51TUF/i
Ocl3UW
41.8 CR373404
46.3 OmyRGT51TUF CA350064
Omy1013UW
47.0 OMM5053 OmyRGT47TUF
49.0 ATP1B3A AAC/CAG152
ACA/CAA390
AS-3
AS-11
Chromosome 28

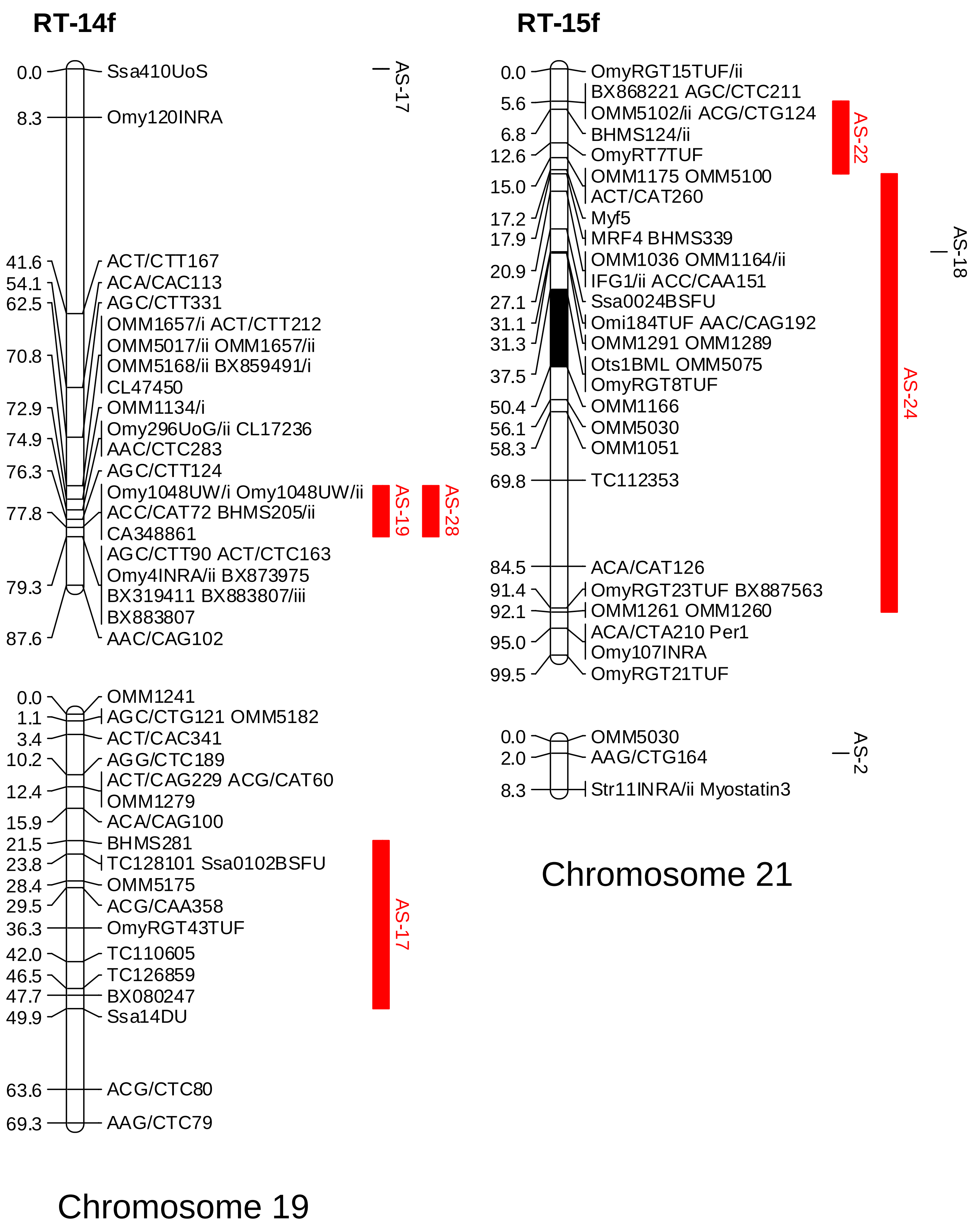
RT-14f
RT-15f
0.0 Ssa410UoS
8.3 Omy120INRA
AS-17
41.6 ACT/CTT167
54.1 ACA/CAC113
62.5 AGC/CTT331
OMM1657/i ACT/CTT212
OMM5017/ii OMM1657/ii
70.8 OMM5168/ii BX859491/i
CL47450
72.9 OMM1134/i
Omy296UoG/ii CL17236
74.9 AAC/CTC283
76.3 AGC/CTT124
Omy1048UW/i Omy1048UW/ii
77.8 ACC/CAT72 BHMS205/ii
CA348861
AGC/CTT90 ACT/CTC163
Omy4INRA/ii BX873975
79.3 BX319411 BX883807/iii
BX883807
87.6 AAC/CAG102
AS-19
AS-28
0.0 OMM1241
1.1 AGC/CTG121 OMM5182
3.4 ACT/CAC341
10.2 AGG/CTC189
ACT/CAG229 ACG/CAT60
12.4 OMM1279
15.9 ACA/CAG100
21.5 BHMS281
23.8 TC128101 Ssa0102BSFU
28.4 OMM5175
29.5 ACG/CAA358
36.3 OmyRGT43TUF
42.0 TC110605
46.5 TC126859
47.7 BX080247
49.9 Ssa14DU
63.6 ACG/CTC80
69.3 AAG/CTC79
AS-17
Chromosome 19
0.0 OmyRGT15TUF/ii
5.6 BX868221 AGC/CTC211
OMM5102/ii ACG/CTG124
6.8 BHMS124/ii
12.6 OmyRT7TUF
15.0 OMM1175 OMM5100
ACT/CAT260
17.2 Myf5
17.9 MRF4 BHMS339
20.9 OMM1036 OMM1164/ii
IFG1/ii ACC/CAA151
27.1 Ssa0024BSFU
31.1 Omi184TUF AAC/CAG192
31.3 OMM1291 OMM1289
37.5 Ots1BML OMM5075
OmyRGT8TUF
50.4 OMM1166
56.1 OMM5030
58.3 OMM1051
69.8 TC112353
84.5 ACA/CAT126
91.4 OmyRGT23TUF BX887563
92.1 OMM1261 OMM1260
95.0 ACA/CTA210 Per1
Omy107INRA
99.5 OmyRGT21TUF
AS-22
AS-24
AS-18
0.0 OMM5030
2.0 AAG/CTG164
8.3 Str11INRA/ii Myostatin3
AS-2
Chromosome 21

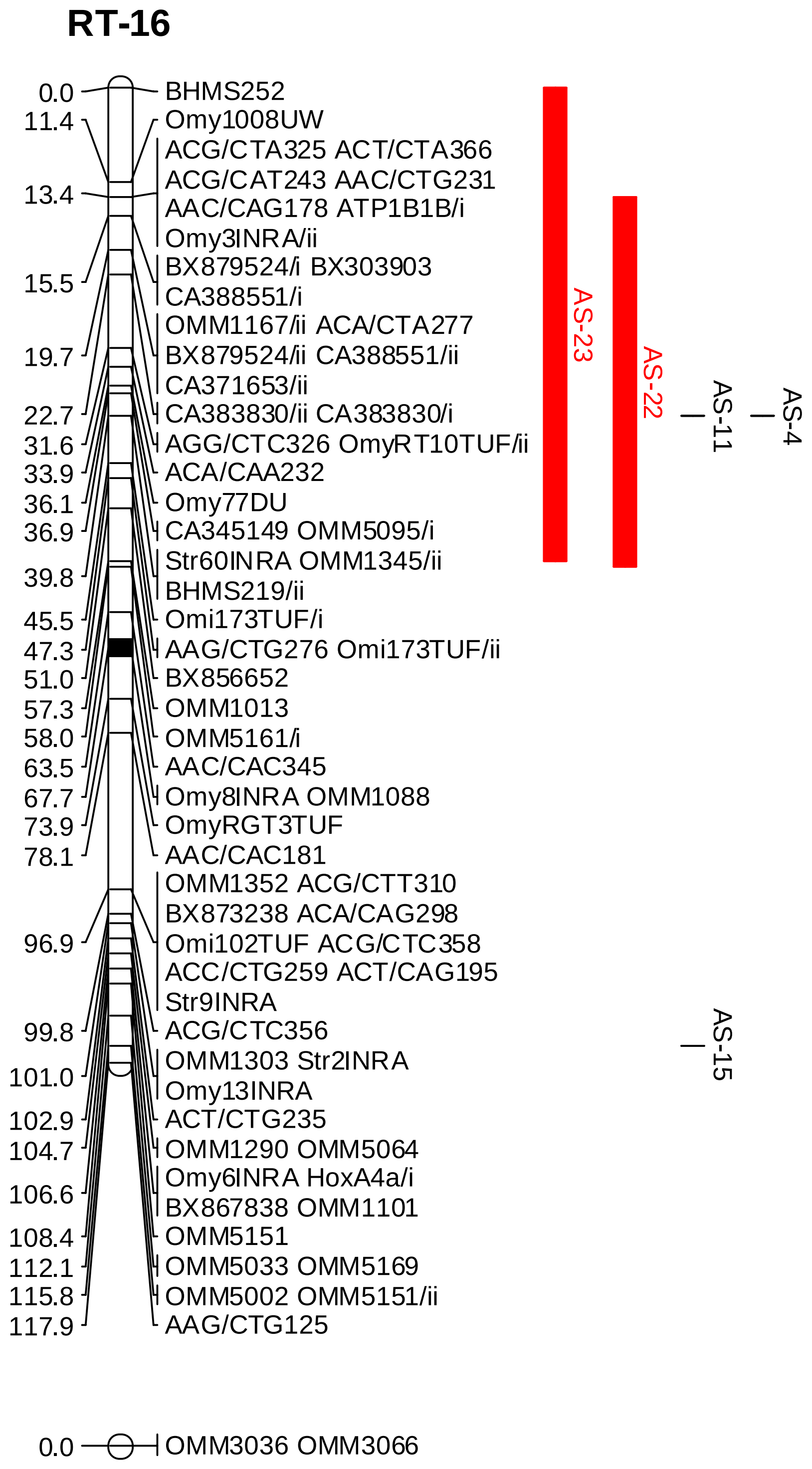

Chromosome 18

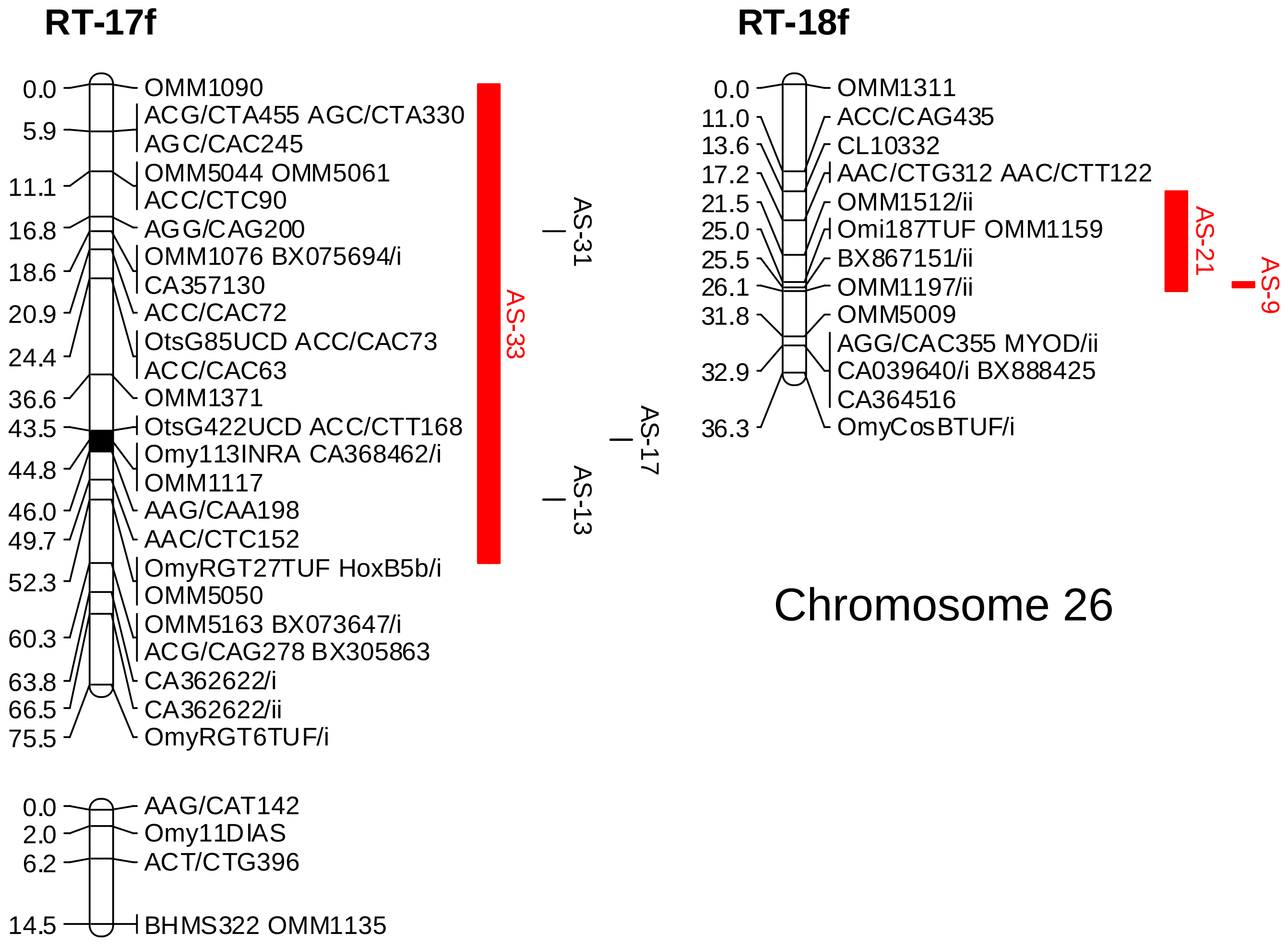

Chromosome 20

Chromosome 26

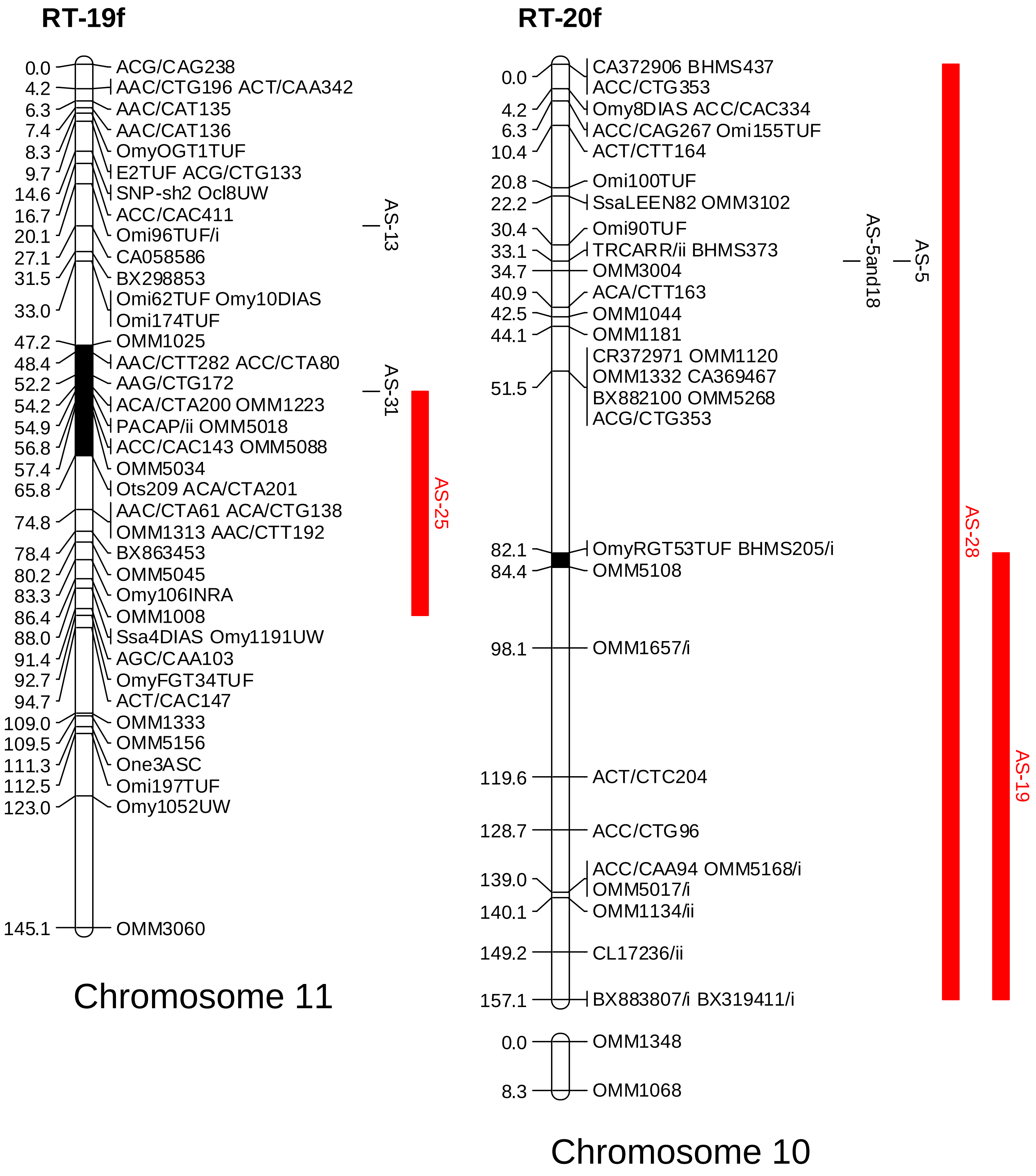

RT-19f
0.0 ACG/CAG238
4.2 AAC/CTG196 ACT/CAA342
6.3 AAC/CAT135
7.4 AAC/CAT136
8.3 OmyOGT1TUF
9.7 E2TUF ACG/CTG133
14.6 SNP-sh2 Ocl8UW
16.7 ACC/CAC411
20.1 Omi96TUF/i
27.1 CA058586
31.5 BX298853
33.0 Omi62TUF Omy10DIAS
Omi174TUF
47.2 OMM1025
48.4 AAC/CTT282 ACC/CTA80
52.2 AAG/CTG172
54.2 ACA/CTA200 OMM1223
54.9 PACAP/ii OMM5018
56.8 ACC/CAC143 OMM5088
57.4 OMM5034
65.8 Ots209 ACA/CTA201
74.8 AAC/CTA61 ACA/CTG138
OMM1313 AAC/CTT192
78.4 BX863453
80.2 OMM5045
83.3 Omy106INRA
86.4 OMM1008
88.0 Ssa4DIAS Omy1191UW
91.4 AGC/CAA103
92.7 OmyFGT34TUF
94.7 ACT/CAC147
109.0 OMM1333
109.5 OMM5156
111.3 One3ASC
112.5 Omi197TUF
123.0 Omy1052UW
145.1 OMM3060
AS-13
AS-31
AS-25
Chromosome 11
RT-20f
0.0 CA372906 BHMS437
ACC/CTG353
4.2 Omy8DIAS ACC/CAC334
6.3 ACC/CAG267 Omi155TUF
10.4 ACT/CTT164
20.8 Omi100TUF
22.2 SsaLEEN82 OMM3102
30.4 Omi90TUF
33.1 TRCARR/ii BHMS373
34.7 OMM3004
40.9 ACA/CTT163
42.5 OMM1044
44.1 OMM1181
51.5 CR372971 OMM1120
OMM1332 CA369467
BX882100 OMM5268
ACG/CTG353
82.1 OmyRGT53TUF BHMS205/i
84.4 OMM5108
98.1 OMM1657/i
119.6 ACT/CTC204
128.7 ACC/CTG96
139.0 ACC/CAA94 OMM5168/i
OMM5017/i
140.1 OMM1134/ii
149.2 CL17236/ii
157.1 BX883807/i BX319411/i
0.0 OMM1348
8.3 OMM1068
AS-5and18
AS-5
AS-28
AS-19
Chromosome 10

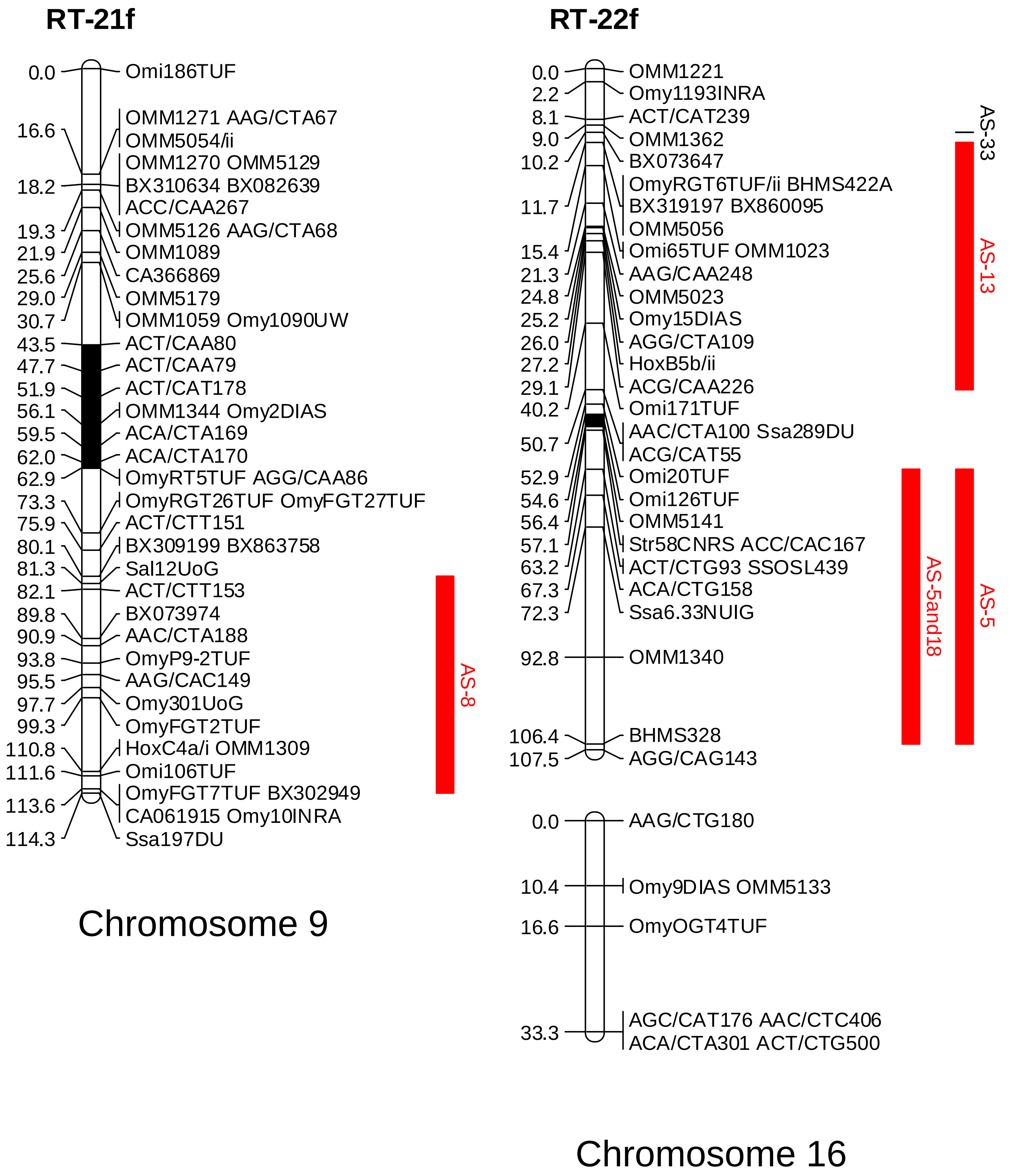

RT-21f
0.0 Omi186TUF
16.6 OMM1271 AAG/CTA67
OMM5054/ii
OMM1270 OMM5129
18.2 BX310634 BX082639
ACC/CAA267
19.3 OMM5126 AAG/CTA68
21.9 OMM1089
25.6 CA366869
29.0 OMM5179
30.7 OMM1059 Omy1090UW
43.5 ACT/CAA80
47.7 ACT/CAA79
51.9 ACT/CAT178
56.1 OMM1344 Omy2DIAS
59.5 ACA/CTA169
62.0 ACA/CTA170
62.9 OmyRT5TUF AGG/CAA86
73.3 OmyRGT26TUF OmyFGT27TUF
75.9 ACT/CTT151
80.1 BX309199 BX863758
81.3 Sal12UoG
82.1 ACT/CTT153
89.8 BX073974
90.9 AAC/CTA188
93.8 OmyP9-2TUF
95.5 AAG/CAC149
97.7 Omy301UoG
99.3 OmyFGT2TUF
110.8 HoxC4a/i OMM1309
111.6 Omi106TUF
113.6 OmyFGT7TUF BX302949
CA061915 Omy10INRA
114.3 Ssa197DU
AS-8
Chromosome 9
RT-22f
0.0 OMM1221
2.2 Omy1193INRA
8.1 ACT/CAT239
9.0 OMM1362
10.2 BX073647
OmyRGT6TUF/ii BHMS422A
11.7 BX319197 BX860095
OMM5056
15.4 Omi65TUF OMM1023
21.3 AAG/CAA248
24.8 OMM5023
25.2 Omy15DIAS
26.0 AGG/CTA109
27.2 HoxB5b/ii
29.1 ACG/CAA226
40.2 Omi171TUF
50.7 AAC/CTA100 Ssa289DU
ACG/CAT55
52.9 Omi20TUF
54.6 Omi126TUF
56.4 OMM5141
57.1 Str58CNRS ACC/CAC167
63.2 ACT/CTG93 SSOSL439
67.3 ACA/CTG158
72.3 Ssa6.33NUIG
92.8 OMM1340
106.4 BHMS328
107.5 AGG/CAG143
0.0 AAG/CTG180
10.4 Omy9DIAS OMM5133
16.6 OmyOGT4TUF
33.3 AGC/CAT176 AAC/CTC406
ACA/CTA301 ACT/CTG500
AS-33
AS-13
AS-5and18
AS-5
Chromosome 16

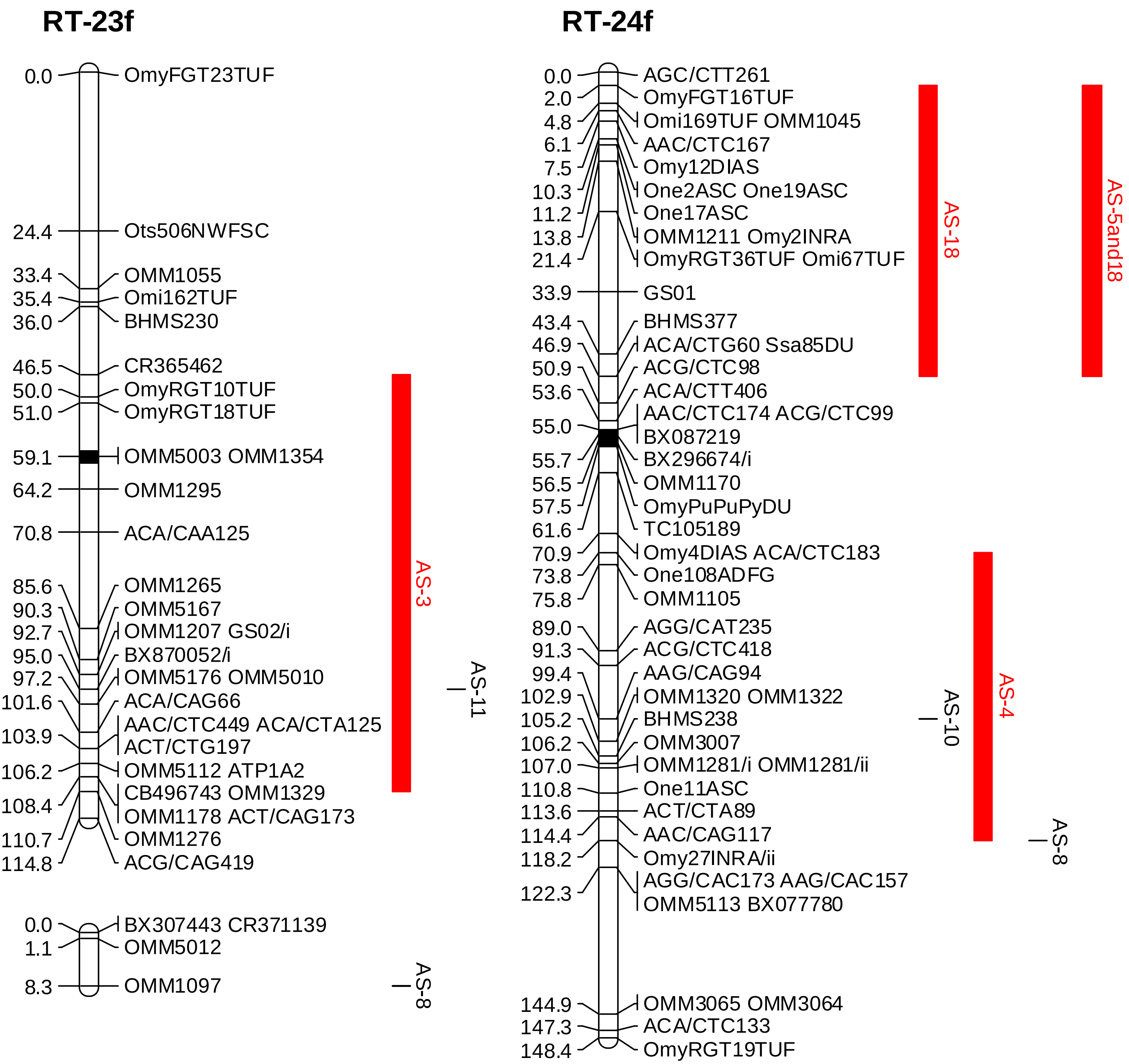

Chromosome 8

Chromosome 4

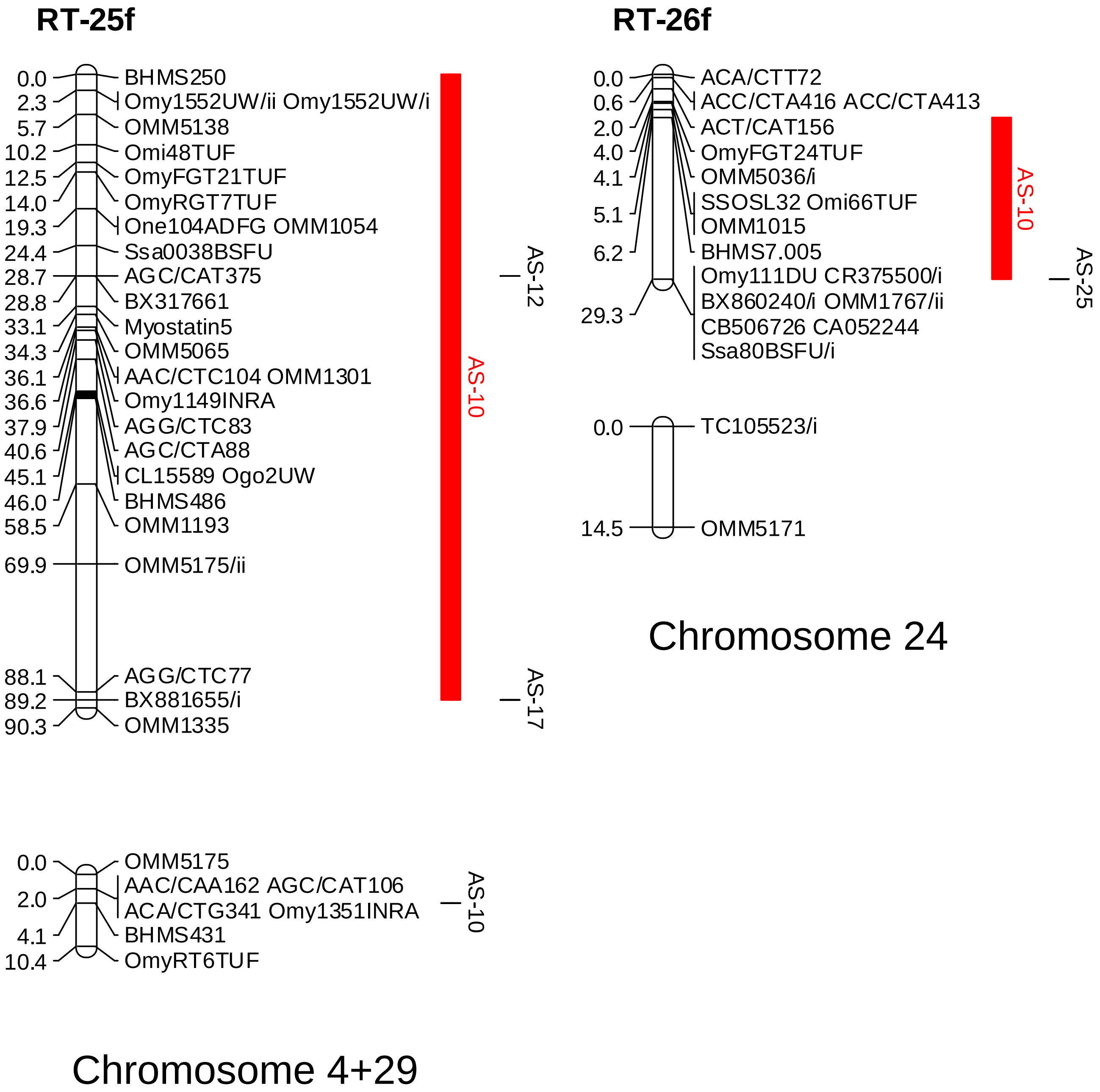
RT-25f
0.0 BHMS250
2.3 Omy1552UW/ii Omy1552UW/i
5.7 OMM5138
10.2 Omi48TUF
12.5 OmyFGT21TUF
14.0 OmyRGT7TUF
19.3 One104ADFG OMM1054
24.4 Ssa0038BSFU
28.7 AGC/CAT375
28.8 BX317661
33.1 Myostatin5
34.3 OMM5065
36.1 AAC/CTC104 OMM1301
36.6 Omy1149INRA
37.9 AGG/CTC83
40.6 AGC/CTA88
45.1 CL15589 Ogo2UW
46.0 BHMS486
58.5 OMM1193
69.9 OMM5175/ii
88.1 AGG/CTC77
89.2 BX881655/i
90.3 OMM1335
AS-10
AS-12
AS-17
0.0 OMM5175
2.0 AAC/CAA162 AGC/CAT106
ACA/CTG341 Omy1351INRA
4.1 BHMS431
10.4 OmyRT6TUF
AS-10
Chromosome 4+29
RT-26f
0.0 ACA/CTT72
0.6 ACC/CTA416 ACC/CTA413
2.0 ACT/CAT156
4.0 OmyFGT24TUF
4.1 OMM5036/i
5.1 SSOSL32 Omi66TUF
OMM1015
6.2 BHMS7.005
Omy111DU CR375500/i
29.3 BX860240/i OMM1767/ii
CB506726 CA052244
Ssa80BSFU/i
AS-10
AS-25
0.0 TC105523/i
14.5 OMM5171
Chromosome 24

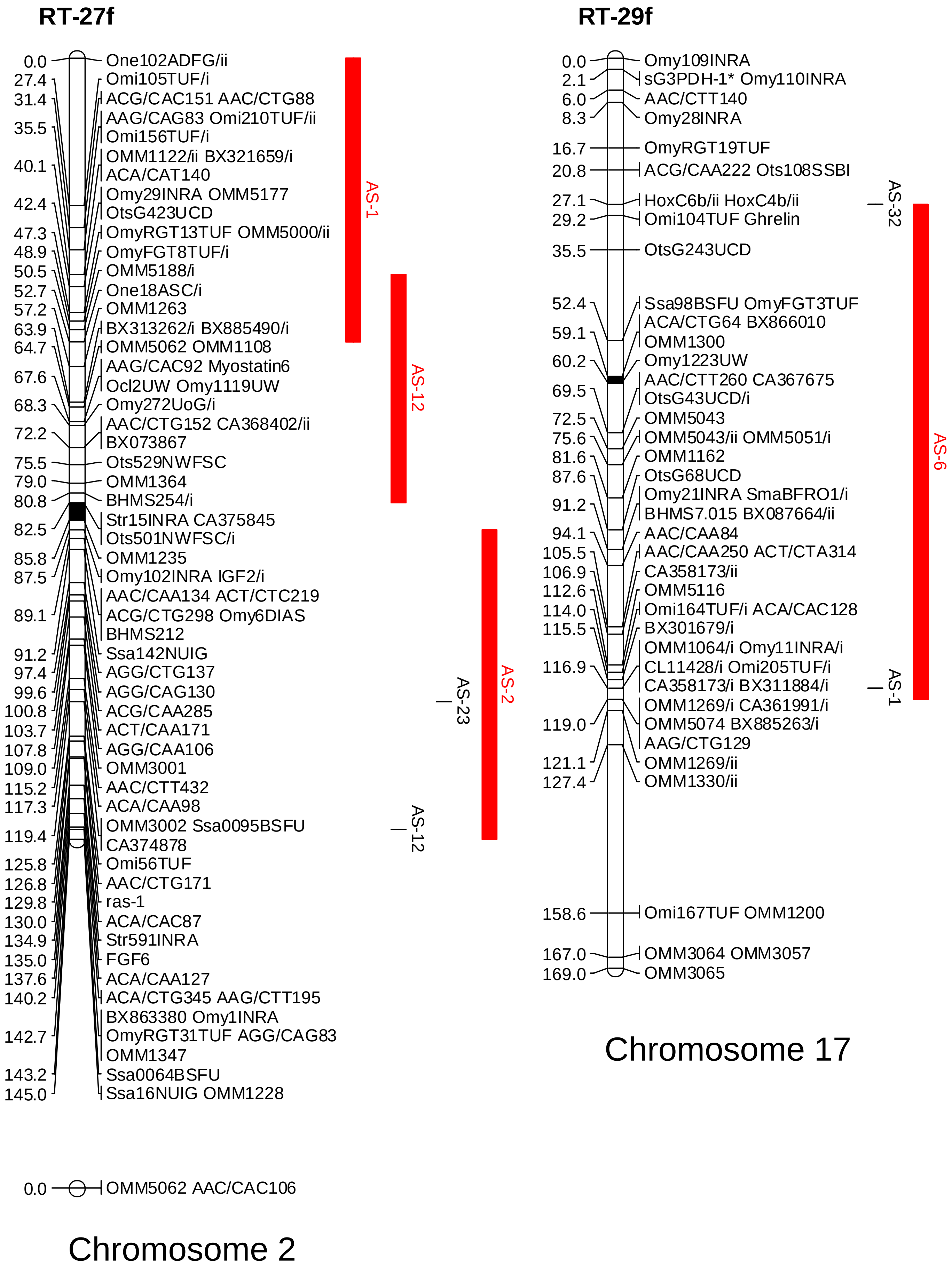

RT-27f
0.0 One102ADFG/ii
27.4 Omi105TUF/i
31.4 ACG/CAC151 AAC/CTG88
35.5 AAG/CAG83 Omi210TUF/ii
Omi156TUF/i
40.1 OMM1122/ii BX321659/i
ACA/CAT140
42.4 Omy29INRA OMM5177
OtsG423UCD
47.3 OmyRGT13TUF OMM5000/ii
48.9 OmyFGT8TUF/i
50.5 OMM5188/i
52.7 One18ASC/i
57.2 OMM1263
63.9 BX313262/i BX885490/i
64.7 OMM5062 OMM1108
67.6 AAG/CAC92 Myostatin6
Ocl2UW Omy1119UW
68.3 Omy272UoG/i
72.2 AAC/CTG152 CA368402/ii
BX073867
75.5 Ots529NWFSC
79.0 OMM1364
80.8 BHMS254/i
82.5 Str15INRA CA375845
Ots501NWFSC/i
85.8 OMM1235
87.5 Omy102INRA IGF2/i
89.1 AAC/CAA134 ACT/CTC219
ACG/CTG298 Omy6DIAS
BHMS212
91.2 Ssa142NUIG
97.4 AGG/CTG137
99.6 AGG/CAG130
100.8 ACG/CAA285
103.7 ACT/CAA171
107.8 AGG/CAA106
109.0 OMM3001
115.2 AAC/CTT432
117.3 ACA/CAA98
119.4 OMM3002 Ssa0095BSFU
CA374878
125.8 Omi56TUF
126.8 AAC/CTG171
129.8 ras-1
130.0 ACA/CAC87
134.9 Str591INRA
135.0 FGF6
137.6 ACA/CAA127
140.2 ACA/CTG345 AAG/CTT195
BX863380 Omy1INRA
142.7 OmyRGT31TUF AGG/CAG83
OMM1347
143.2 Ssa0064BSFU
145.0 Ssa16NUIG OMM1228
0.0 OMM5062 AAC/CAC106
AS-1
AS-12
AS-2
AS-23
AS-12
Chromosome 2
RT-29f
0.0 Omy109INRA
2.1 sG3PDH-1* Omy110INRA
6.0 AAC/CTT140
8.3 Omy28INRA
16.7 OmyRGT19TUF
20.8 ACG/CAA222 Ots108SSBI
27.1 HoxC6b/ii HoxC4b/ii
29.2 Omi104TUF Ghrelin
35.5 OtsG243UCD
52.4 Ssa98BSFU OmyFGT3TUF
59.1 ACA/CTG64 BX866010
OMM1300
60.2 Omy1223UW
69.5 AAC/CTT260 CA367675
OtsG43UCD/i
72.5 OMM5043
75.6 OMM5043/ii OMM5051/i
81.6 OMM1162
87.6 OtsG68UCD
91.2 Omy21INRA SmaBFRO1/i
BHMS7.015 BX087664/ii
94.1 AAC/CAA84
105.5 AAC/CAA250 ACT/CTA314
106.9 CA358173/ii
112.6 OMM5116
114.0 Omi164TUF/i ACA/CAC128
115.5 BX301679/i
OMM1064/i Omy11INRA/i
116.9 CL11428/i Omi205TUF/i
CA358173/i BX311884/i
OMM1269/i CA361991/i
119.0 OMM5074 BX885263/i
AAG/CTG129
121.1 OMM1269/ii
127.4 OMM1330/ii
158.6 Omi167TUF OMM1200
167.0 OMM3064 OMM3057
169.0 OMM3065
AS-32
AS-6
AS-1
Chromosome 17

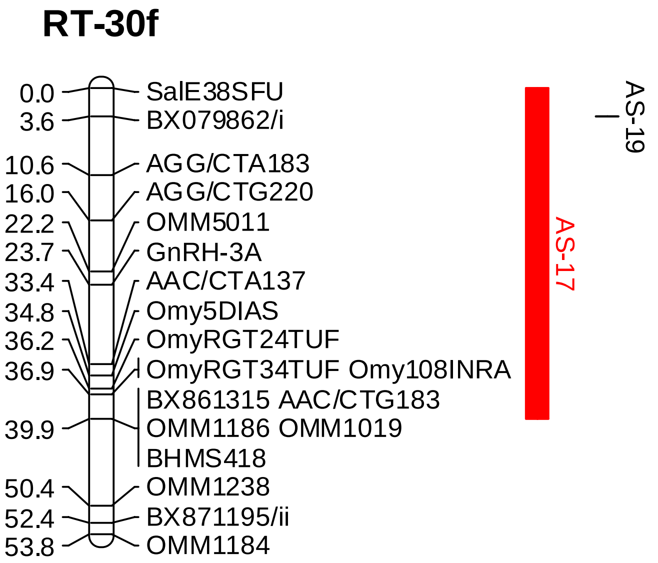

Chromosome 23

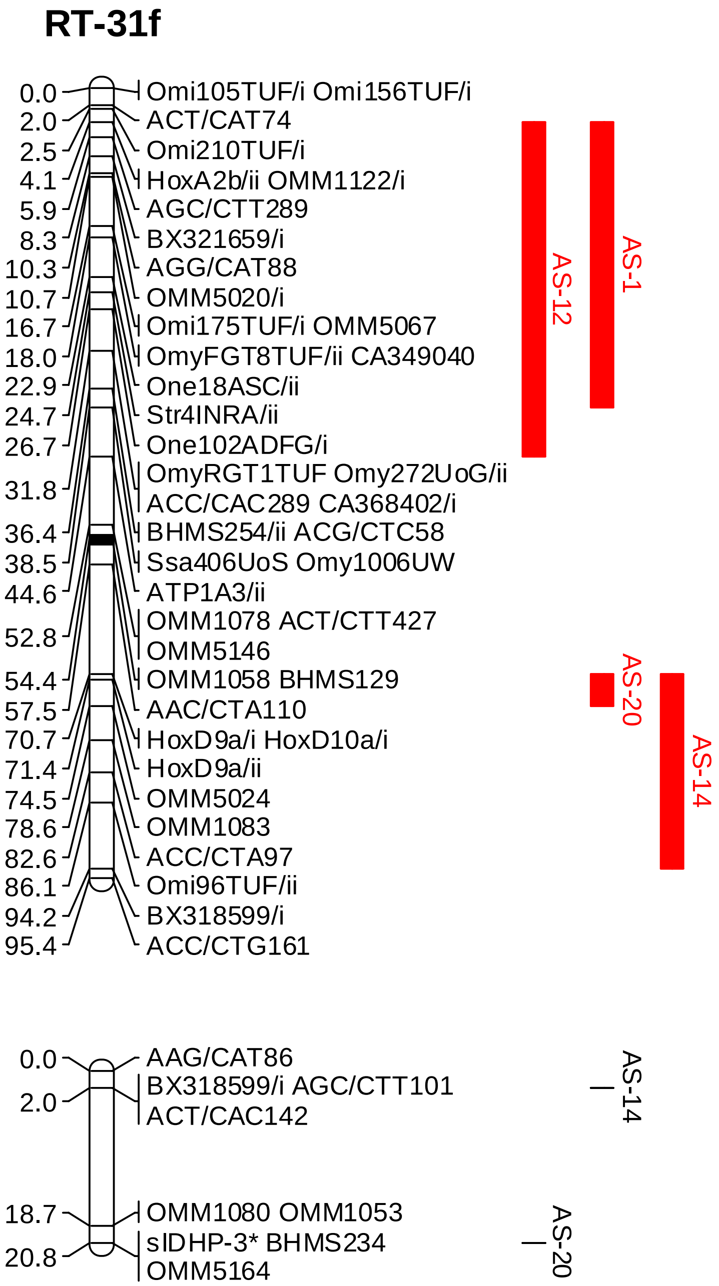

Chromosome 3
