# Supplementary material for: Distribution of ancestral proto-Actinopterygian chromosome arms within the genomes of 4R-derivative salmonid fishes (Rainbow trout and Atlantic salmon)
Source: BMC Genomics. 2008 Nov 25;9:557. doi: 10.1186/1471-2164-9-557 (PMC2632648; doi:10.1186/1471-2164-9-557)
Supplement: Additional File 12 — s_figure2-asvsrt_homology.pdf. Depiction of homologous rainbow trout linkage group blocks within the Atlantic salmon genome. Merged Atlantic salmon female maps are used as a template. [file 1471-2164-9-557-S12.pdf]

Supplementary Figure 2: Atlantic salmon merged female genetic map depicting all linkage groups numbered in sequence. The 'f' suffix following the linkage group name indicates it is a female map. Map distances are indicated as observed recombination distances (Θ) where 1% recombination = 1 cM. Homologous regions within Atlantic salmon to their respective rainbow trout linkage groups are indicated to the right of each linkage group. Atlantic salmon chromosomes ranked according to size (Phillips et al. unpublished) are indicated below each linkage group designation.

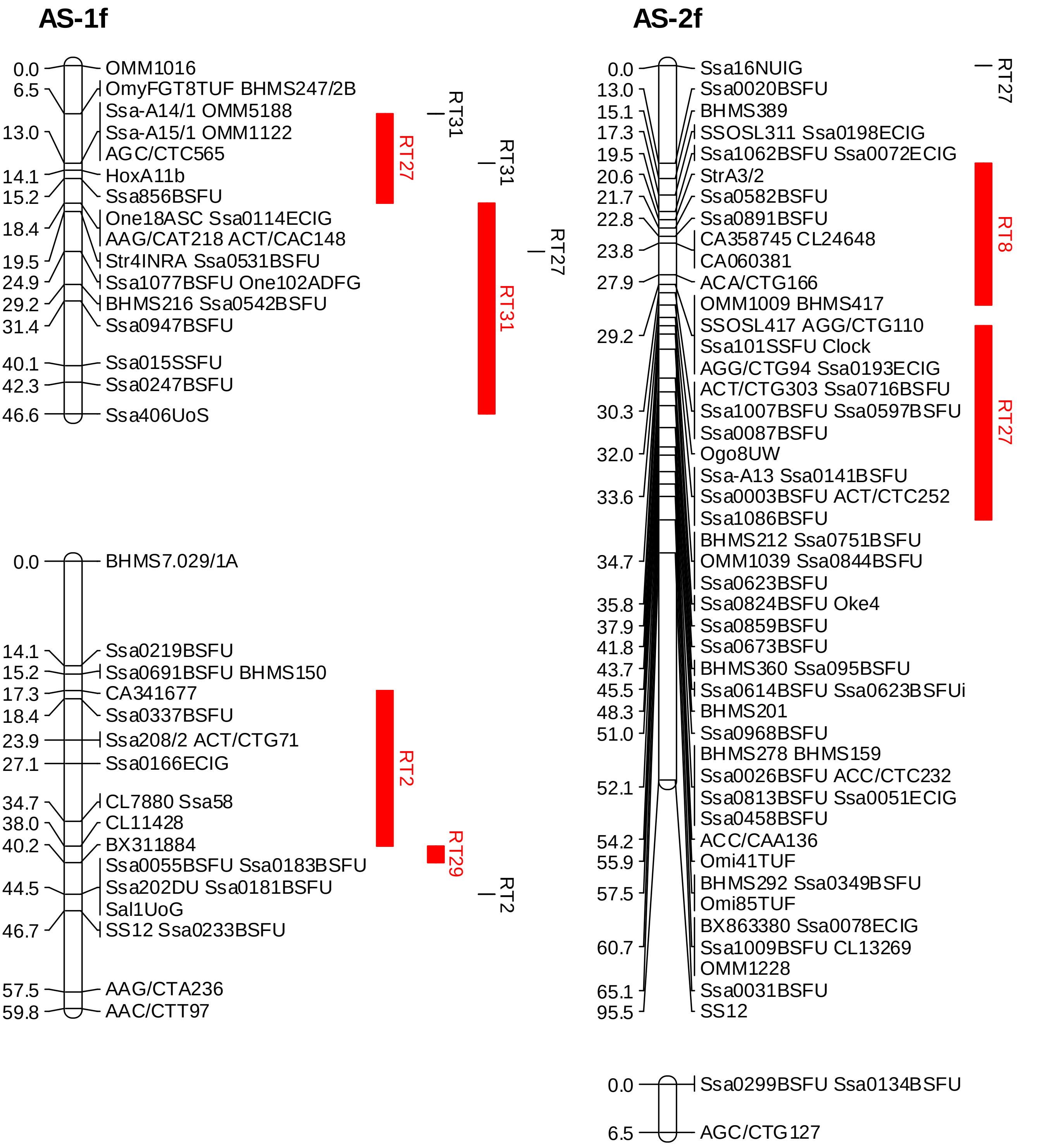
AS-1f
AS-2f
0.0 OMM1016
6.5 OmyFGT8TUF BHMS247/2B
Ssa-A14/1 OMM5188
13.0 Ssa-A15/1 OMM1122
AGC/CTC565
14.1 HoxA11b
15.2 Ssa856BSFU
One18ASC Ssa0114ECIG
18.4 AAG/CAT218 ACT/CAC148
19.5 Str4INRA Ssa0531BSFU
24.9 Ssa1077BSFU One102ADFG
29.2 BHMS216 Ssa0542BSFU
31.4 Ssa0947BSFU
40.1 Ssa015SSFU
42.3 Ssa0247BSFU
46.6 Ssa406UoS
RT27
RT31
RT31
RT31
RT27
0.0 BHMS7.029/1A
14.1 Ssa0219BSFU
15.2 Ssa0691BSFU BHMS150
17.3 CA341677
18.4 Ssa0337BSFU
23.9 Ssa208/2 ACT/CTG71
27.1 Ssa0166ECIG
34.7 CL7880 Ssa58
38.0 CL11428
40.2 BX311884
Ssa0055BSFU Ssa0183BSFU
44.5 Ssa202DU Ssa0181BSFU
Sal1UoG
46.7 SS12 Ssa0233BSFU
57.5 AAG/CTA236
59.8 AAC/CTT97
RT2
RT29
RT2
0.0 Ssa16NUIG
13.0 Ssa0020BSFU
15.1 BHMS389
17.3 SSOSL311 Ssa0198ECIG
19.5 Ssa1062BSFU Ssa0072ECIG
20.6 StrA3/2
21.7 Ssa0582BSFU
22.8 Ssa0891BSFU
23.8 CA358745 CL24648
CA060381
27.9 ACA/CTG166
OMM1009 BHMS417
29.2 SSOSL417 AGG/CTG110
Ssa101SSFU Clock
AGG/CTG94 Ssa0193ECIG
ACT/CTG303 Ssa0716BSFU
30.3 Ssa1007BSFU Ssa0597BSFU
Ssa0087BSFU
32.0 Ogo8UW
Ssa-A13 Ssa0141BSFU
33.6 Ssa0003BSFU ACT/CTC252
Ssa1086BSFU
BHMS212 Ssa0751BSFU
34.7 OMM1039 Ssa0844BSFU
Ssa0623BSFU
35.8 Ssa0824BSFU Oke4
37.9 Ssa0859BSFU
41.8 Ssa0673BSFU
43.7 BHMS360 Ssa095BSFU
45.5 Ssa0614BSFU Ssa0623BSFUi
48.3 BHMS201
51.0 Ssa0968BSFU
BHMS278 BHMS159
52.1 Ssa0026BSFU ACC/CTC232
Ssa0813BSFU Ssa0051ECIG
Ssa0458BSFU
54.2 ACC/CAA136
55.9 Omi41TUF
57.5 BHMS292 Ssa0349BSFU
Omi85TUF
BX863380 Ssa0078ECIG
60.7 Ssa1009BSFU CL13269
OMM1228
65.1 Ssa0031BSFU
95.5 SS12
RT27
RT8
RT27
0.0 Ssa0299BSFU Ssa0134BSFU
6.5 AGC/CTG127

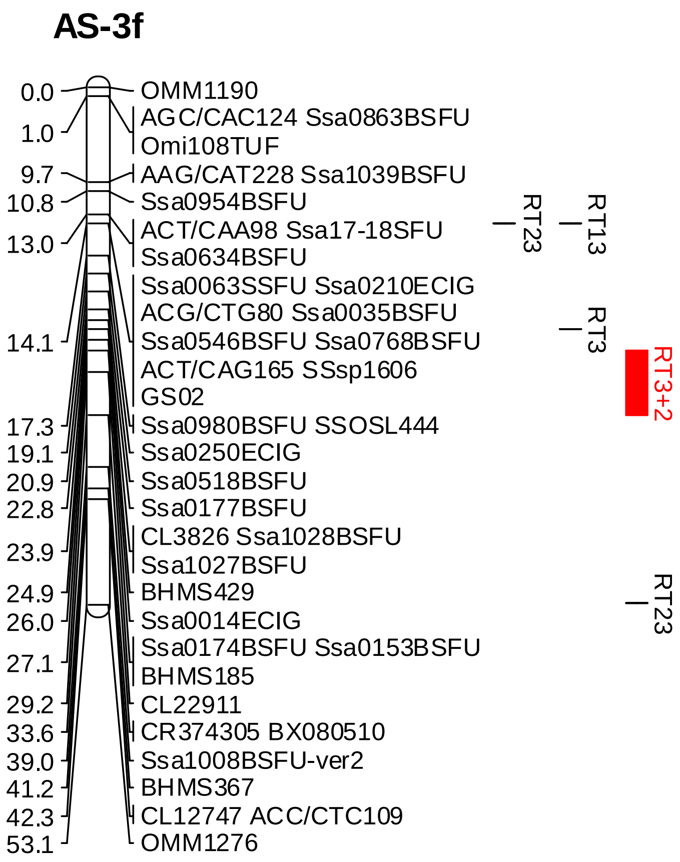
AS-3f
0.0
1.0
9.7
10.8
13.0
14.1
17.3
19.1
20.9
22.8
23.9
24.9
26.0
27.1
29.2
33.6
39.0
41.2
42.3
53.1
OMM1190
AGC/CAC124 Ssa0863BSFU
Omi108TUF
AAG/CAT228 Ssa1039BSFU
Ssa0954BSFU
ACT/CAA98 Ssa17-18SFU
Ssa0634BSFU
Ssa0063SSFU Ssa0210ECIG
ACG/CTG80 Ssa0035BSFU
Ssa0546BSFU Ssa0768BSFU
ACT/CAG165 SSsp1606
GS02
Ssa0980BSFU SSOSL444
Ssa0250ECIG
Ssa0518BSFU
Ssa0177BSFU
CL3826 Ssa1028BSFU
Ssa1027BSFU
BHMS429
Ssa0014ECIG
Ssa0174BSFU Ssa0153BSFU
BHMS185
CL22911
CR374305 BX080510
Ssa1008BSFU-ver2
BHMS367
CL12747 ACC/CTC109
OMM1276
RT23
RT13
RT3
RT3+2
RT23

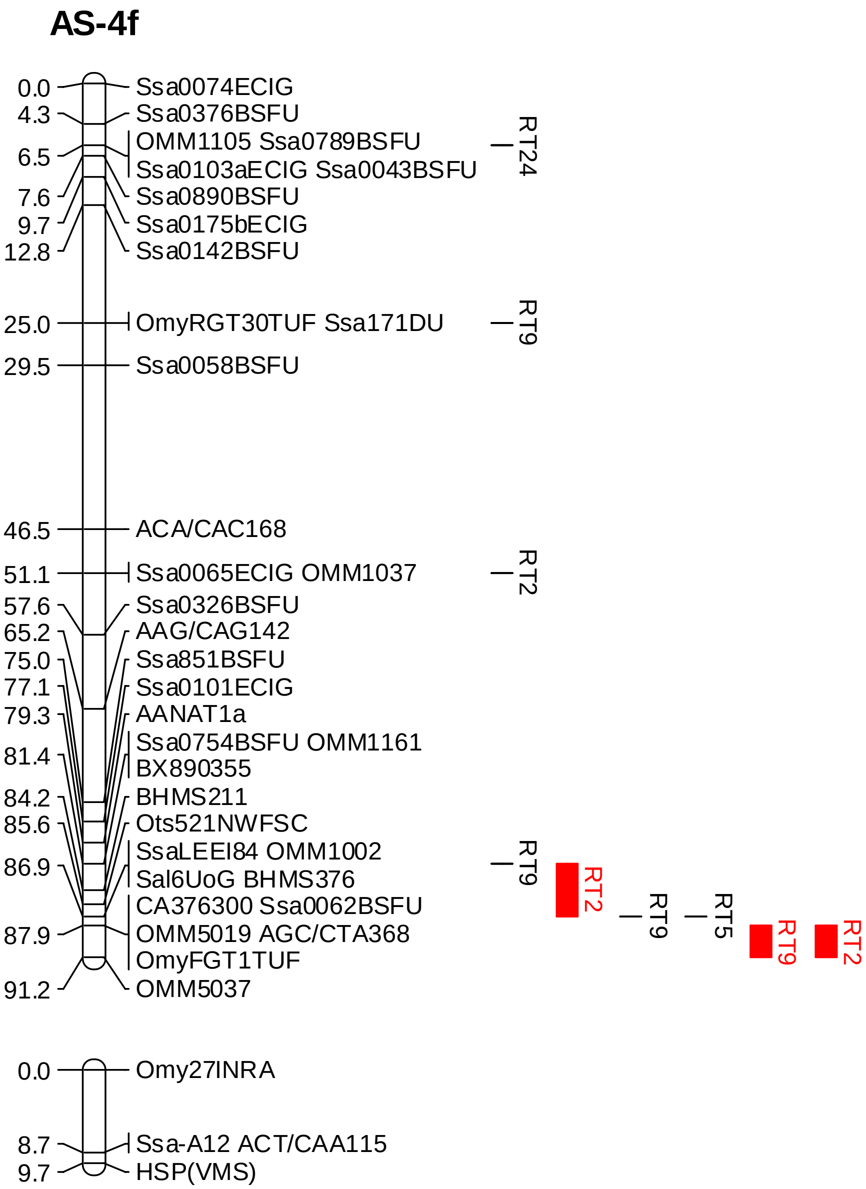
AS-4f
0.0 Ssa0074ECIG
4.3 Ssa0376BSFU
6.5 OMM1105 Ssa0789BSFU
Ssa0103aECIG Ssa0043BSFU
7.6 Ssa0890BSFU
9.7 Ssa0175bECIG
12.8 Ssa0142BSFU
25.0 OmyRGT30TUF Ssa171DU
29.5 Ssa0058BSFU
46.5 ACA/CAC168
51.1 Ssa0065ECIG OMM1037
57.6 Ssa0326BSFU
65.2 AAG/CAG142
75.0 Ssa851BSFU
77.1 Ssa0101ECIG
79.3 AANAT1a
81.4 Ssa0754BSFU OMM1161
BX890355
84.2 BHMS211
85.6 Ots521NWFSC
86.9 SsaLEEI84 OMM1002
Sal6UoG BHMS376
CA376300 Ssa0062BSFU
87.9 OMM5019 AGC/CTA368
OmyFGT1TUF
91.2 OMM5037
RT24
RT9
RT2
RT9
RT2
RT9
RT5
RT9
RT2
0.0 Omy27INRA
8.7 Ssa-A12 ACT/CAA115
9.7 HSP(VMS)

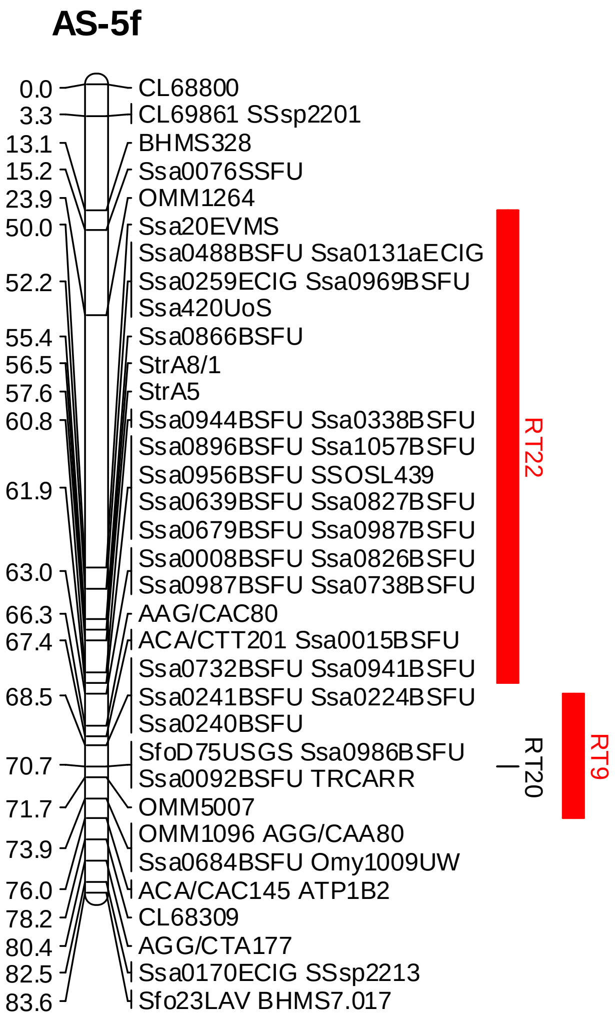
AS-5f
0.0
3.3
13.1
15.2
23.9
50.0
52.2
55.4
56.5
57.6
60.8
61.9
63.0
66.3
67.4
68.5
70.7
71.7
73.9
76.0
78.2
80.4
82.5
83.6
CL68800
CL69861 SSsp2201
BHMS328
Ssa0076SSFU
OMM1264
Ssa20EVMS
Ssa0488BSFU Ssa0131aECIG
Ssa0259ECIG Ssa0969BSFU
Ssa420UoS
Ssa0866BSFU
StrA8/1
StrA5
Ssa0944BSFU Ssa0338BSFU
Ssa0896BSFU Ssa1057BSFU
Ssa0956BSFU SSOSL439
Ssa0639BSFU Ssa0827BSFU
Ssa0679BSFU Ssa0987BSFU
Ssa0008BSFU Ssa0826BSFU
Ssa0987BSFU Ssa0738BSFU
AAG/CAC80
ACA/CTT201 Ssa0015BSFU
Ssa0732BSFU Ssa0941BSFU
Ssa0241BSFU Ssa0224BSFU
Ssa0240BSFU
SfoD75USGS Ssa0986BSFU
Ssa0092BSFU TRCARR
OMM5007
OMM1096 AGG/CAA80
Ssa0684BSFU Omy1009UW
ACA/CAC145 ATP1B2
CL68309
AGG/CTA177
Ssa0170ECIG SSsp2213
Sfo23LAV BHMS7.017
RT22
RT20
RT9

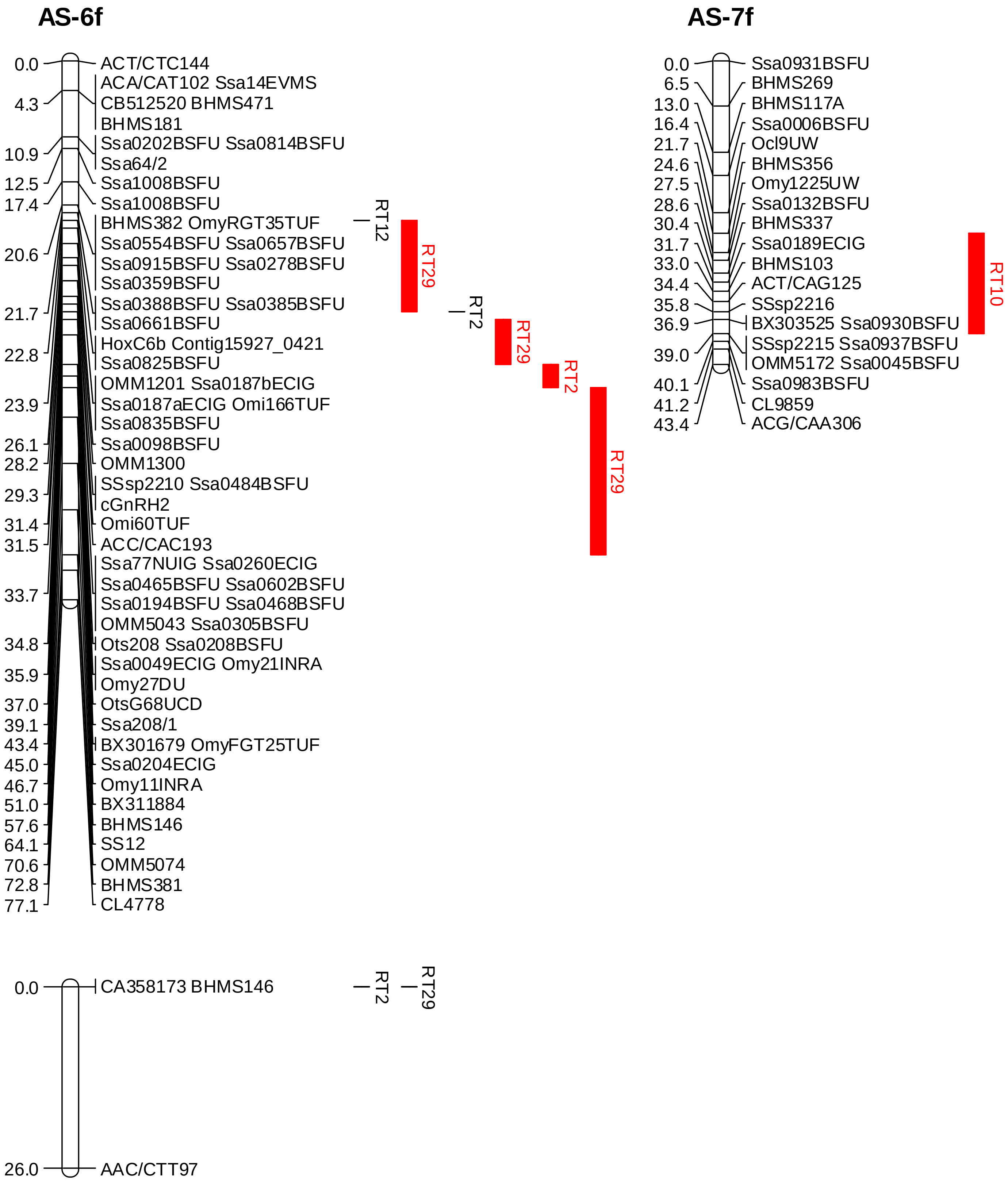
AS-6f
AS-7f
0.0 ACT/CTC144
ACA/CAT102 Ssa14EVMS
4.3 CB512520 BHMS471
BHMS181
Ssa0202BSFU Ssa0814BSFU
10.9 Ssa64/2
12.5 Ssa1008BSFU
17.4 Ssa1008BSFU
BHMS382 OmyRGT35TUF
20.6 Ssa0554BSFU Ssa0657BSFU
Ssa0915BSFU Ssa0278BSFU
Ssa0359BSFU
21.7 Ssa0388BSFU Ssa0385BSFU
Ssa0661BSFU
22.8 HoxC6b Contig15927_0421
Ssa0825BSFU
23.9 OMM1201 Ssa0187bECIG
Ssa0187aECIG Omi166TUF
Ssa0835BSFU
26.1 Ssa0098BSFU
28.2 OMM1300
29.3 SSsp2210 Ssa0484BSFU
cGnRH2
31.4 Omi60TUF
31.5 ACC/CAC193
33.7 Ssa77NUIG Ssa0260ECIG
Ssa0465BSFU Ssa0602BSFU
Ssa0194BSFU Ssa0468BSFU
OMM5043 Ssa0305BSFU
34.8 Ots208 Ssa0208BSFU
35.9 Ssa0049ECIG Omy21INRA
Omy27DU
37.0 OtsG68UCD
39.1 Ssa208/1
43.4 BX301679 OmyFGT25TUF
45.0 Ssa0204ECIG
46.7 Omy11INRA
51.0 BX311884
57.6 BHMS146
64.1 SS12
70.6 OMM5074
72.8 BHMS381
77.1 CL4778
RT12
RT29
RT2
RT29
RT2
RT29
0.0 CA358173 BHMS146
26.0 AAC/CTT97
RT2
RT29
0.0 Ssa0931BSFU
6.5 BHMS269
13.0 BHMS117A
16.4 Ssa0006BSFU
21.7 Ocl9UW
24.6 BHMS356
27.5 Omy1225UW
28.6 Ssa0132BSFU
30.4 BHMS337
31.7 Ssa0189ECIG
33.0 BHMS103
34.4 ACT/CAG125
35.8 SSsp2216
36.9 BX303525 Ssa0930BSFU
39.0 SSsp2215 Ssa0937BSFU
OMM5172 Ssa0045BSFU
40.1 Ssa0983BSFU
41.2 CL9859
43.4 ACG/CAA306
RT10

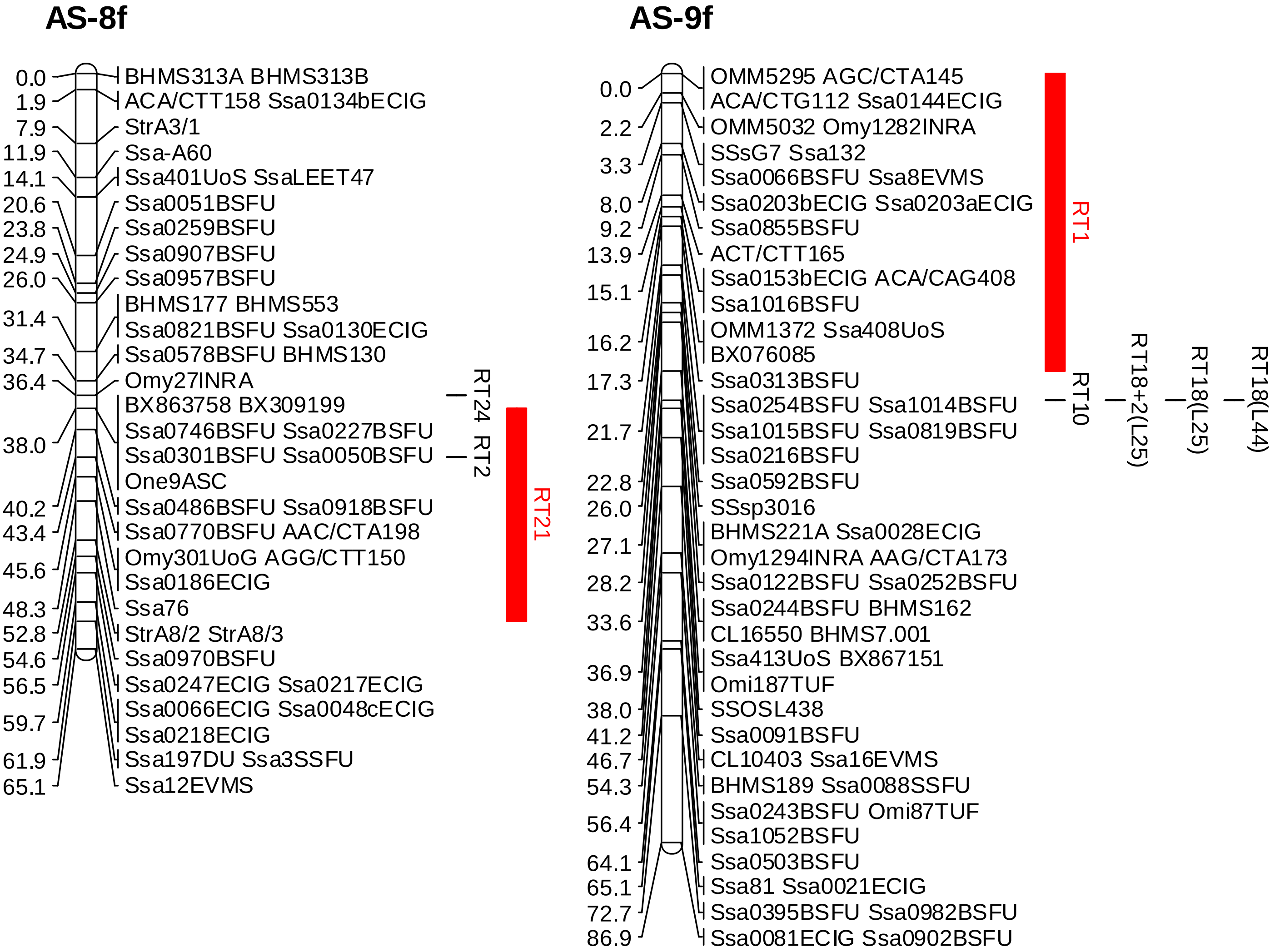
AS-8f
0.0 BHMS313A BHMS313B
1.9 ACA/CTT158 Ssa0134bECIG
7.9 StrA3/1
11.9 Ssa-A60
14.1 Ssa401UoS SsaLEET47
20.6 Ssa0051BSFU
23.8 Ssa0259BSFU
24.9 Ssa0907BSFU
26.0 Ssa0957BSFU
31.4 BHMS177 BHMS553
Ssa0821BSFU Ssa0130ECIG
34.7 Ssa0578BSFU BHMS130
36.4 Omy27INRA
BX863758 BX309199
38.0 Ssa0746BSFU Ssa0227BSFU
Ssa0301BSFU Ssa0050BSFU
One9ASC
40.2 Ssa0486BSFU Ssa0918BSFU
43.4 Ssa0770BSFU AAC/CTA198
45.6 Omy301UoG AGG/CTT150
Ssa0186ECIG
48.3 Ssa76
52.8 StrA8/2 StrA8/3
54.6 Ssa0970BSFU
56.5 Ssa0247ECIG Ssa0217ECIG
59.7 Ssa0066ECIG Ssa0048cECIG
Ssa0218ECIG
61.9 Ssa197DU Ssa3SSFU
65.1 Ssa12EVMS
RT24
RT2
RT21
AS-9f
0.0 OMM5295 AGC/CTA145
ACA/CTG112 Ssa0144ECIG
2.2 OMM5032 Omy1282INRA
SSsG7 Ssa132
3.3 Ssa0066BSFU Ssa8EVMS
8.0 Ssa0203bECIG Ssa0203aECIG
9.2 Ssa0855BSFU
13.9 ACT/CTT165
Ssa0153bECIG ACA/CAG408
15.1 Ssa1016BSFU
OMM1372 Ssa408UoS
16.2 BX076085
17.3 Ssa0313BSFU
Ssa0254BSFU Ssa1014BSFU
21.7 Ssa1015BSFU Ssa0819BSFU
Ssa0216BSFU
22.8 Ssa0592BSFU
26.0 SSsp3016
BHMS221A Ssa0028ECIG
27.1 Omy1294INRA AAG/CTA173
28.2 Ssa0122BSFU Ssa0252BSFU
Ssa0244BSFU BHMS162
33.6 CL16550 BHMS7.001
Ssa413UoS BX867151
36.9 Omi187TUF
38.0 SSOSL438
41.2 Ssa0091BSFU
46.7 CL10403 Ssa16EVMS
54.3 BHMS189 Ssa0088SSFU
Ssa0243BSFU Omi87TUF
56.4 Ssa1052BSFU
64.1 Ssa0503BSFU
65.1 Ssa81 Ssa0021ECIG
72.7 Ssa0395BSFU Ssa0982BSFU
86.9 Ssa0081ECIG Ssa0902BSFU
RT1
RT10
RT18+2(L25)
RT18(L25)
RT18(L44)

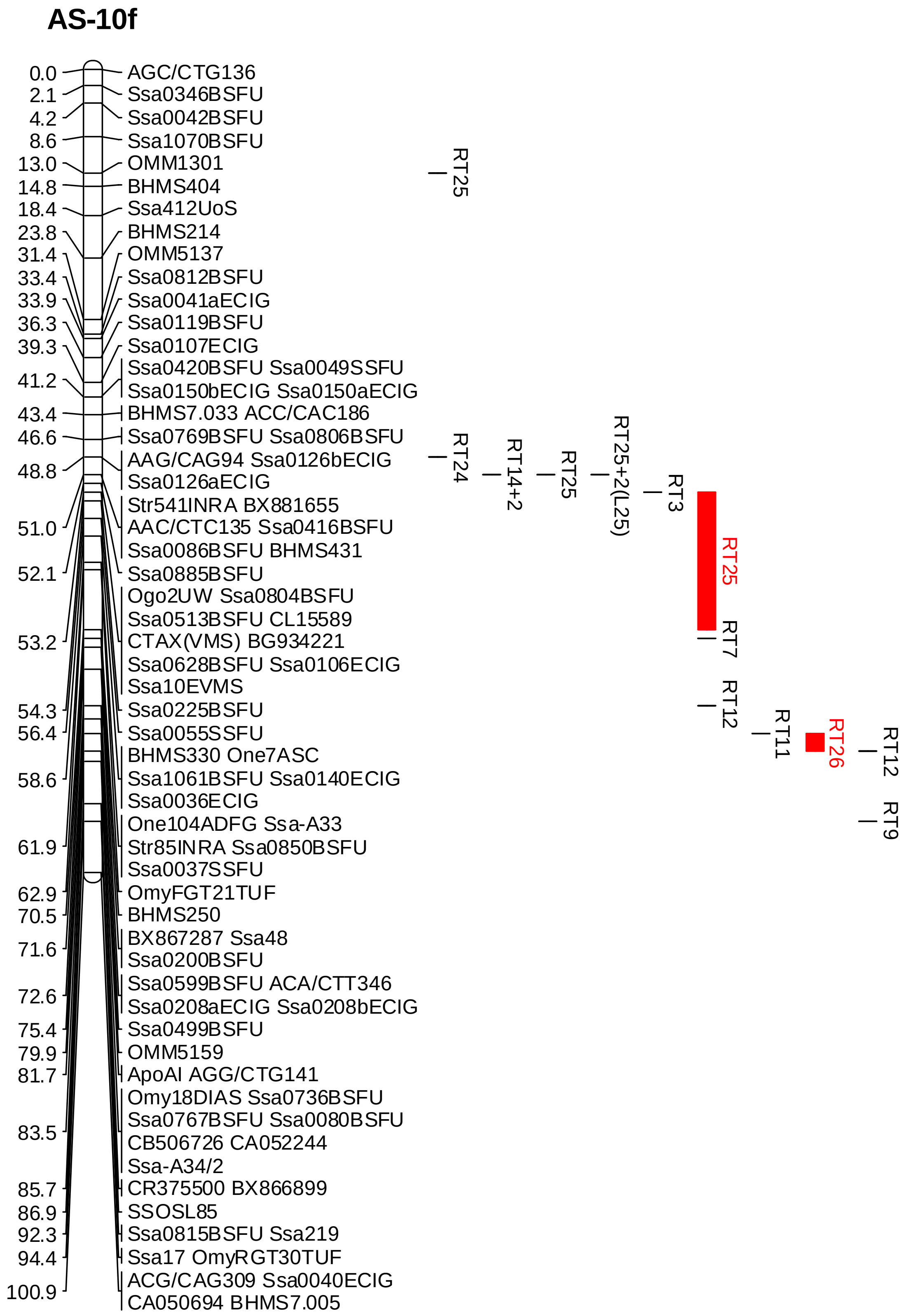
AS-10f
0.0 AGC/CTG136
2.1 Ssa0346BSFU
4.2 Ssa0042BSFU
8.6 Ssa1070BSFU
13.0 OMM1301
14.8 BHMS404
18.4 Ssa412UoS
23.8 BHMS214
31.4 OMM5137
33.4 Ssa0812BSFU
33.9 Ssa0041aECIG
36.3 Ssa0119BSFU
39.3 Ssa0107ECIG
41.2 Ssa0420BSFU Ssa0049SSFU
Ssa0150bECIG Ssa0150aECIG
43.4 BHMS7.033 ACC/CAC186
46.6 Ssa0769BSFU Ssa0806BSFU
48.8 AAG/CAG94 Ssa0126bECIG
Ssa0126aECIG
Str541INRA BX881655
51.0 AAC/CTC135 Ssa0416BSFU
Ssa0086BSFU BHMS431
52.1 Ssa0885BSFU
Ogo2UW Ssa0804BSFU
Ssa0513BSFU CL15589
53.2 CTAX(VMS) BG934221
Ssa0628BSFU Ssa0106ECIG
Ssa10EVMS
54.3 Ssa0225BSFU
56.4 Ssa0055SSFU
BHMS330 One7ASC
58.6 Ssa1061BSFU Ssa0140ECIG
Ssa0036ECIG
One104ADFG Ssa-A33
61.9 Str85INRA Ssa0850BSFU
Ssa0037SSFU
62.9 OmyFGT21TUF
70.5 BHMS250
71.6 BX867287 Ssa48
Ssa0200BSFU
72.6 Ssa0599BSFU ACA/CTT346
Ssa0208aECIG Ssa0208bECIG
75.4 Ssa0499BSFU
79.9 OMM5159
81.7 ApoAI AGG/CTG141
Omy18DIAS Ssa0736BSFU
83.5 Ssa0767BSFU Ssa0080BSFU
CB506726 CA052244
Ssa-A34/2
85.7 CR375500 BX866899
86.9 SSOSL85
92.3 Ssa0815BSFU Ssa219
94.4 Ssa17 OmyRGT30TUF
100.9 ACG/CAG309 Ssa0040ECIG
CA050694 BHMS7.005
RT25
RT24
RT14+2
RT25
RT25+2(L25)
RT3
RT25
RT7
RT12
RT11
RT26
RT12
RT9

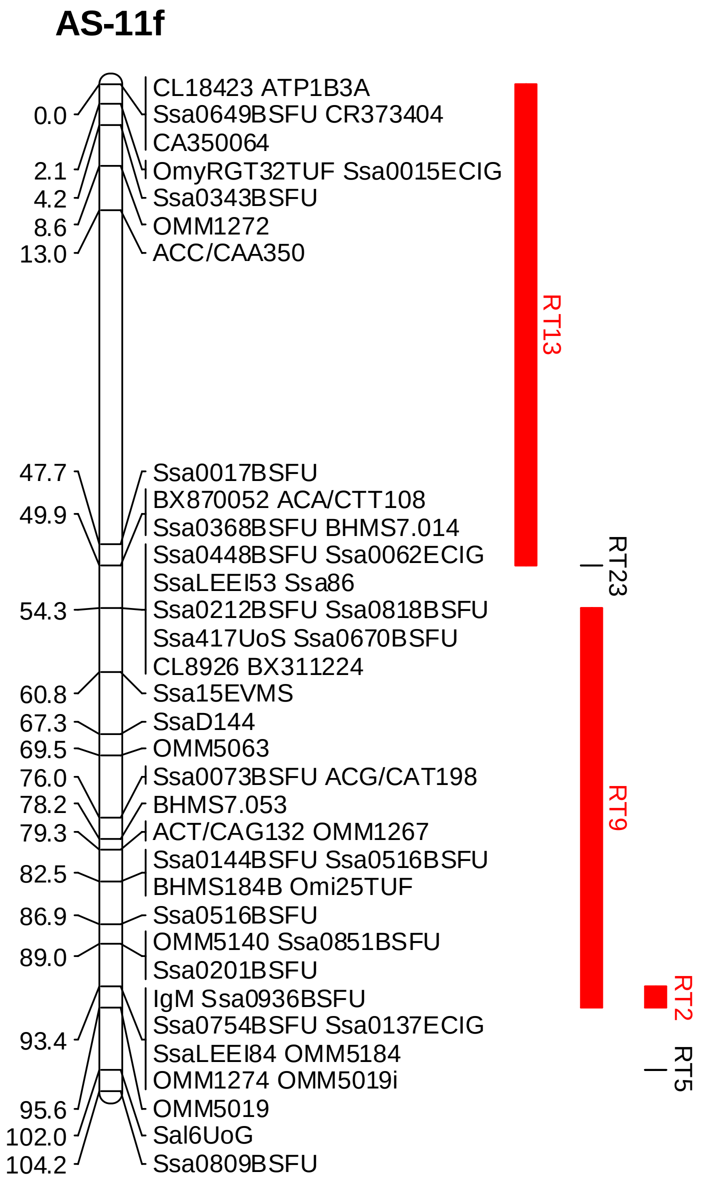
AS-11f
0.0
2.1
4.2
8.6
13.0
47.7
49.9
54.3
60.8
67.3
69.5
76.0
78.2
79.3
82.5
86.9
89.0
93.4
95.6
102.0
104.2
CL18423 ATP1B3A
Ssa0649BSFU CR373404
CA350064
OmyRGT32TUF Ssa0015ECIG
Ssa0343BSFU
OMM1272
ACC/CAA350
Ssa0017BSFU
BX870052 ACA/CTT108
Ssa0368BSFU BHMS7.014
Ssa0448BSFU Ssa0062ECIG
SsaLEEI53 Ssa86
Ssa0212BSFU Ssa0818BSFU
Ssa417UoS Ssa0670BSFU
CL8926 BX311224
Ssa15EVMS
SsaD144
OMM5063
Ssa0073BSFU ACG/CAT198
BHMS7.053
ACT/CAG132 OMM1267
Ssa0144BSFU Ssa0516BSFU
BHMS184B Omi25TUF
Ssa0516BSFU
OMM5140 Ssa0851BSFU
Ssa0201BSFU
IgM Ssa0936BSFU
Ssa0754BSFU Ssa0137ECIG
SsaLEEI84 OMM5184
OMM1274 OMM5019i
OMM5019
Sal6UoG
Ssa0809BSFU
RT13
RT23
RT9
RT2
RT5

AS-12f

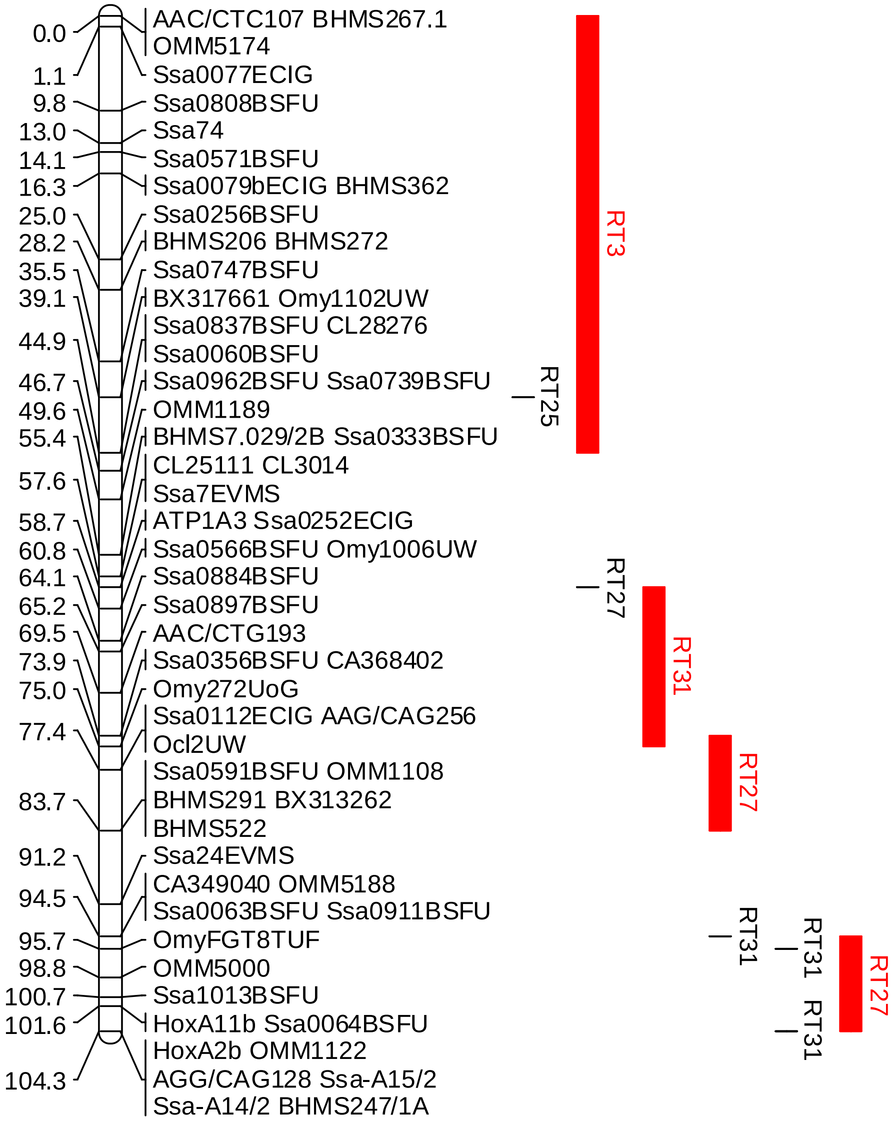

0.0
1.1
9.8
13.0
14.1
16.3
25.0
28.2
35.5
39.1
44.9
46.7
49.6
55.4
57.6
58.7
60.8
64.1
65.2
69.5
73.9
75.0
77.4
83.7
91.2
94.5
95.7
98.8
100.7
101.6
104.3
AAC/CTC107 BHMS267.1
OMM5174
Ssa0077ECIG
Ssa0808BSFU
Ssa74
Ssa0571BSFU
Ssa0079bECIG BHMS362
Ssa0256BSFU
BHMS206 BHMS272
Ssa0747BSFU
BX317661 Omy1102UW
Ssa0837BSFU CL28276
Ssa0060BSFU
Ssa0962BSFU Ssa0739BSFU
OMM1189
BHMS7.029/2B Ssa0333BSFU
CL25111 CL3014
Ssa7EVMS
ATP1A3 Ssa0252ECIG
Ssa0566BSFU Omy1006UW
Ssa0884BSFU
Ssa0897BSFU
AAC/CTG193
Ssa0356BSFU CA368402
Omy272UoG
Ssa0112ECIG AAG/CAG256
Ocl2UW
Ssa0591BSFU OMM1108
BHMS291 BX313262
BHMS522
Ssa24EVMS
CA349040 OMM5188
Ssa0063BSFU Ssa0911BSFU
OmyFGT8TUF
OMM5000
Ssa1013BSFU
HoxA11b Ssa0064BSFU
HoxA2b OMM1122
AGG/CAG128 Ssa-A15/2
Ssa-A14/2 BHMS247/1A
RT3
RT25
RT27
RT31
RT27
RT31
RT31
RT27
RT31

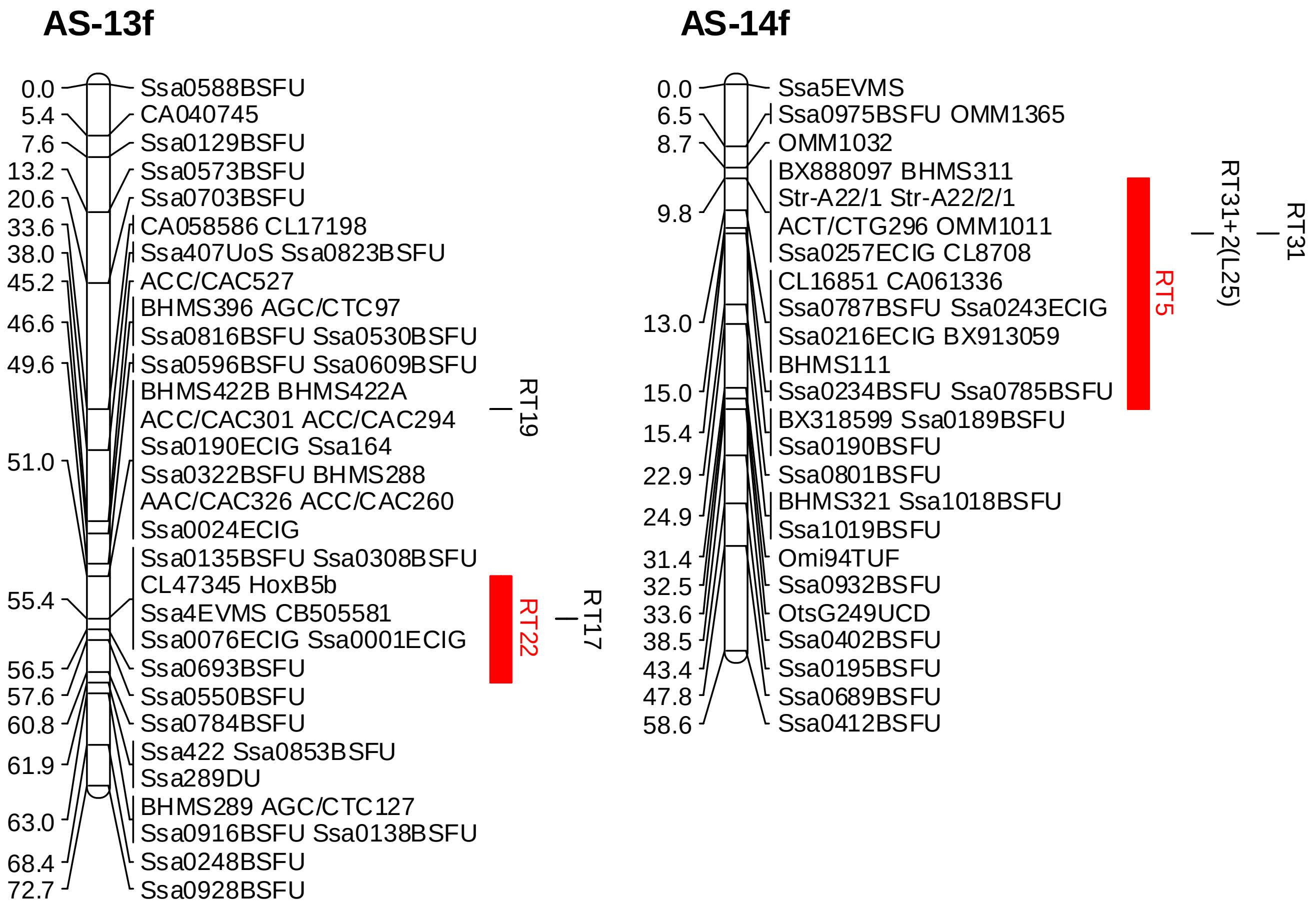
AS-13f
0.0 Ssa0588BSFU
5.4 CA040745
7.6 Ssa0129BSFU
13.2 Ssa0573BSFU
20.6 Ssa0703BSFU
33.6 CA058586 CL17198
38.0 Ssa407UoS Ssa0823BSFU
45.2 ACC/CAC527
46.6 BHMS396 AGC/CTC97
Ssa0816BSFU Ssa0530BSFU
49.6 Ssa0596BSFU Ssa0609BSFU
BHMS422B BHMS422A
ACC/CAC301 ACC/CAC294
Ssa0190ECIG Ssa164
51.0 Ssa0322BSFU BHMS288
AAC/CAC326 ACC/CAC260
Ssa0024ECIG
Ssa0135BSFU Ssa0308BSFU
CL47345 HoxB5b
55.4 Ssa4EVMS CB505581
Ssa0076ECIG Ssa0001ECIG
56.5 Ssa0693BSFU
57.6 Ssa0550BSFU
60.8 Ssa0784BSFU
61.9 Ssa422 Ssa0853BSFU
Ssa289DU
63.0 BHMS289 AGC/CTC127
Ssa0916BSFU Ssa0138BSFU
68.4 Ssa0248BSFU
72.7 Ssa0928BSFU
RT19
RT22
RT17
AS-14f
0.0 Ssa5EVMS
6.5 Ssa0975BSFU OMM1365
8.7 OMM1032
BX888097 BHMS311
Str-A22/1 Str-A22/2/1
9.8 ACT/CTG296 OMM1011
Ssa0257ECIG CL8708
CL16851 CA061336
Ssa0787BSFU Ssa0243ECIG
13.0 Ssa0216ECIG BX913059
BHMS111
15.0 Ssa0234BSFU Ssa0785BSFU
15.4 BX318599 Ssa0189BSFU
Ssa0190BSFU
22.9 Ssa0801BSFU
24.9 BHMS321 Ssa1018BSFU
Ssa1019BSFU
31.4 Omi94TUF
32.5 Ssa0932BSFU
33.6 OtsG249UCD
38.5 Ssa0402BSFU
43.4 Ssa0195BSFU
47.8 Ssa0689BSFU
58.6 Ssa0412BSFU
RT5
RT31+2(L25)
RT31

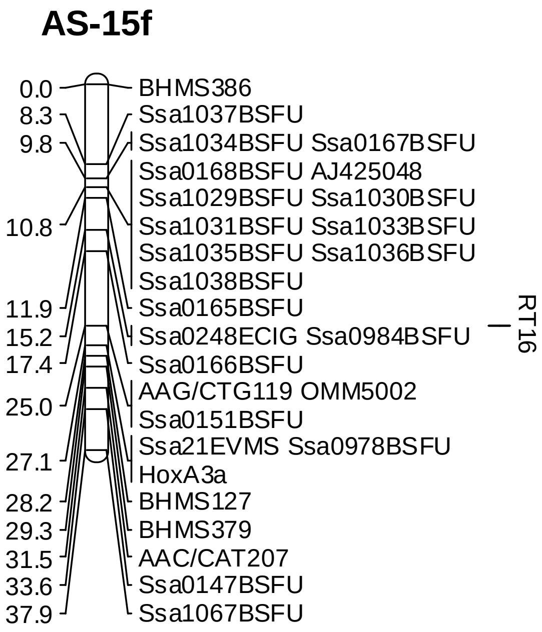
AS-15f
0.0
8.3
9.8
10.8
11.9
15.2
17.4
25.0
27.1
28.2
29.3
31.5
33.6
37.9
BHMS386
Ssa1037BSFU
Ssa1034BSFU Ssa0167BSFU
Ssa0168BSFU AJ425048
Ssa1029BSFU Ssa1030BSFU
Ssa1031BSFU Ssa1033BSFU
Ssa1035BSFU Ssa1036BSFU
Ssa1038BSFU
Ssa0165BSFU
Ssa0248ECIG Ssa0984BSFU
Ssa0166BSFU
AAG/CTG119 OMM5002
Ssa0151BSFU
Ssa21EVMS Ssa0978BSFU
HoxA3a
BHMS127
BHMS379
AAC/CAT207
Ssa0147BSFU
Ssa1067BSFU
RT16

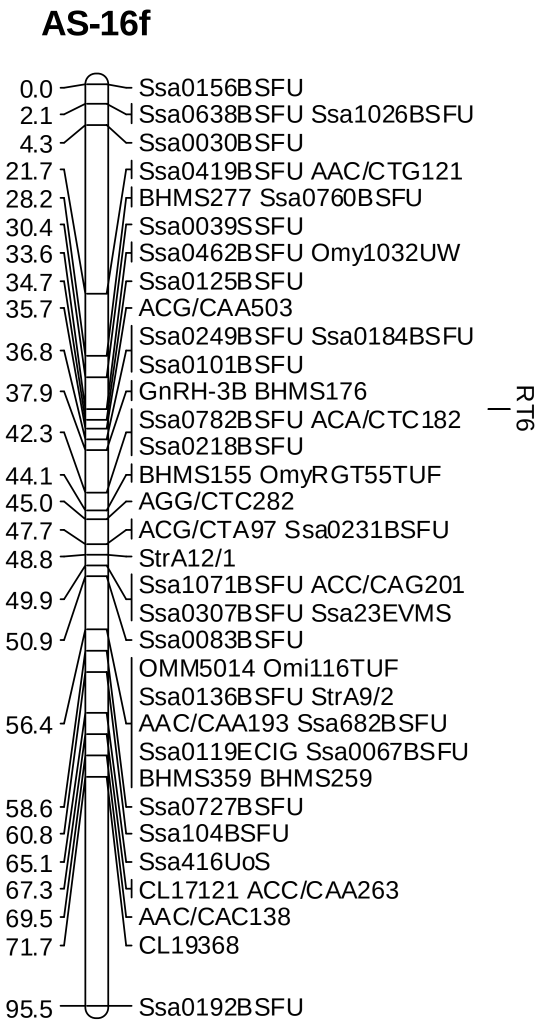
AS-16f
0.0
2.1
4.3
21.7
28.2
30.4
33.6
34.7
35.7
36.8
37.9
42.3
44.1
45.0
47.7
48.8
49.9
50.9
56.4
58.6
60.8
65.1
67.3
69.5
71.7
95.5
Ssa0156BSFU
Ssa0638BSFU Ssa1026BSFU
Ssa0030BSFU
Ssa0419BSFU AAC/CTG121
BHMS277 Ssa0760BSFU
Ssa0039SSFU
Ssa0462BSFU Omy1032UW
Ssa0125BSFU
ACG/CAA503
Ssa0249BSFU Ssa0184BSFU
Ssa0101BSFU
GnRH-3B BHMS176
Ssa0782BSFU ACA/CTC182
Ssa0218BSFU
BHMS155 OmyRGT55TUF
AGG/CTC282
ACG/CTA97 Ssa0231BSFU
StrA12/1
Ssa1071BSFU ACC/CAG201
Ssa0307BSFU Ssa23EVMS
Ssa0083BSFU
OMM5014 Omi116TUF
Ssa0136BSFU StrA9/2
AAC/CAA193 Ssa682BSFU
Ssa0119ECIG Ssa0067BSFU
BHMS359 BHMS259
Ssa0727BSFU
Ssa104BSFU
Ssa416UoS
CL17121 ACC/CAA263
AAC/CAC138
CL19368
Ssa0192BSFU
RT6

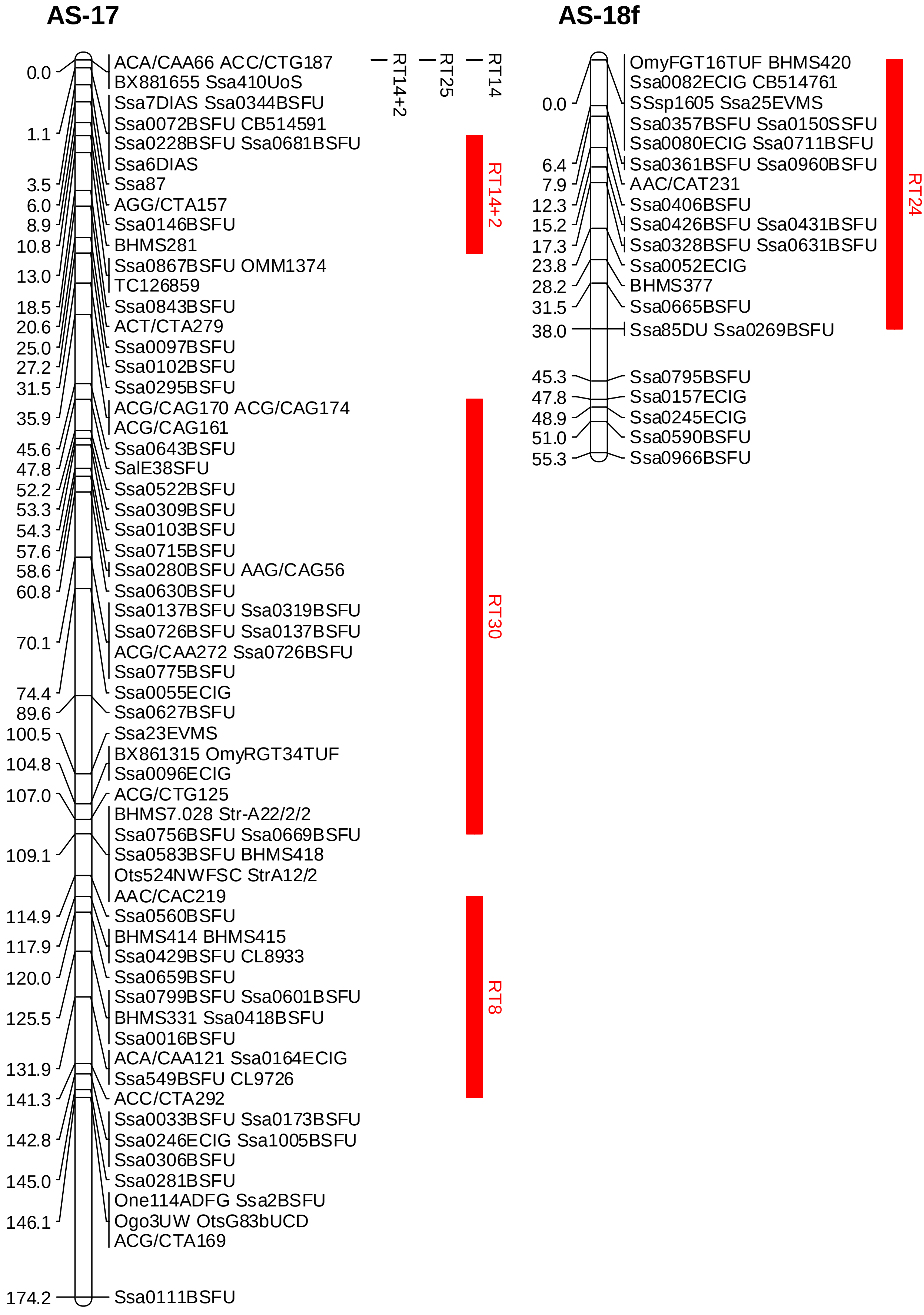

AS-17
AS-18f
0.0
1.1
3.5
6.0
8.9
10.8
13.0
18.5
20.6
25.0
27.2
31.5
35.9
45.6
47.8
52.2
53.3
54.3
57.6
58.6
60.8
70.1
74.4
89.6
100.5
104.8
107.0
109.1
114.9
117.9
120.0
125.5
131.9
141.3
142.8
145.0
146.1
174.2
ACA/CAA66 ACC/CTG187
BX881655 Ssa410UoS
Ssa7DIAS Ssa0344BSFU
Ssa0072BSFU CB514591
Ssa0228BSFU Ssa0681BSFU
Ssa6DIAS
Ssa87
AGG/CTA157
Ssa0146BSFU
BHMS281
Ssa0867BSFU OMM1374
TC126859
Ssa0843BSFU
ACT/CTA279
Ssa0097BSFU
Ssa0102BSFU
Ssa0295BSFU
ACG/CAG170 ACG/CAG174
ACG/CAG161
Ssa0643BSFU
SalE38SFU
Ssa0522BSFU
Ssa0309BSFU
Ssa0103BSFU
Ssa0715BSFU
Ssa0280BSFU AAG/CAG56
Ssa0630BSFU
Ssa0137BSFU Ssa0319BSFU
Ssa0726BSFU Ssa0137BSFU
ACG/CAA272 Ssa0726BSFU
Ssa0775BSFU
Ssa0055ECIG
Ssa0627BSFU
Ssa23EVMS
BX861315 OmyRGT34TUF
Ssa0096ECIG
ACG/CTG125
BHMS7.028 Str-A22/2/2
Ssa0756BSFU Ssa0669BSFU
Ssa0583BSFU BHMS418
Ots524NWFSC StrA12/2
AAC/CAC219
Ssa0560BSFU
BHMS414 BHMS415
Ssa0429BSFU CL8933
Ssa0659BSFU
Ssa0799BSFU Ssa0601BSFU
BHMS331 Ssa0418BSFU
Ssa0016BSFU
ACA/CAA121 Ssa0164ECIG
Ssa549BSFU CL9726
ACC/CTA292
Ssa0033BSFU Ssa0173BSFU
Ssa0246ECIG Ssa1005BSFU
Ssa0306BSFU
Ssa0281BSFU
One114ADFG Ssa2BSFU
Ogo3UW OtsG83bUCD
ACG/CTA169
Ssa0111BSFU
RT14+2
RT25
RT14
RT14+2
RT30
RT8
0.0
6.4
7.9
12.3
15.2
17.3
23.8
28.2
31.5
38.0
45.3
47.8
48.9
51.0
55.3
OmyFGT16TUF BHMS420
Ssa0082ECIG CB514761
SSsp1605 Ssa25EVMS
Ssa0357BSFU Ssa0150SSFU
Ssa0080ECIG Ssa0711BSFU
Ssa0361BSFU Ssa0960BSFU
AAC/CAT231
Ssa0406BSFU
Ssa0426BSFU Ssa0431BSFU
Ssa0328BSFU Ssa0631BSFU
Ssa0052ECIG
BHMS377
Ssa0665BSFU
Ssa85DU Ssa0269BSFU
Ssa0795BSFU
Ssa0157ECIG
Ssa0245ECIG
Ssa0590BSFU
Ssa0966BSFU
RT24

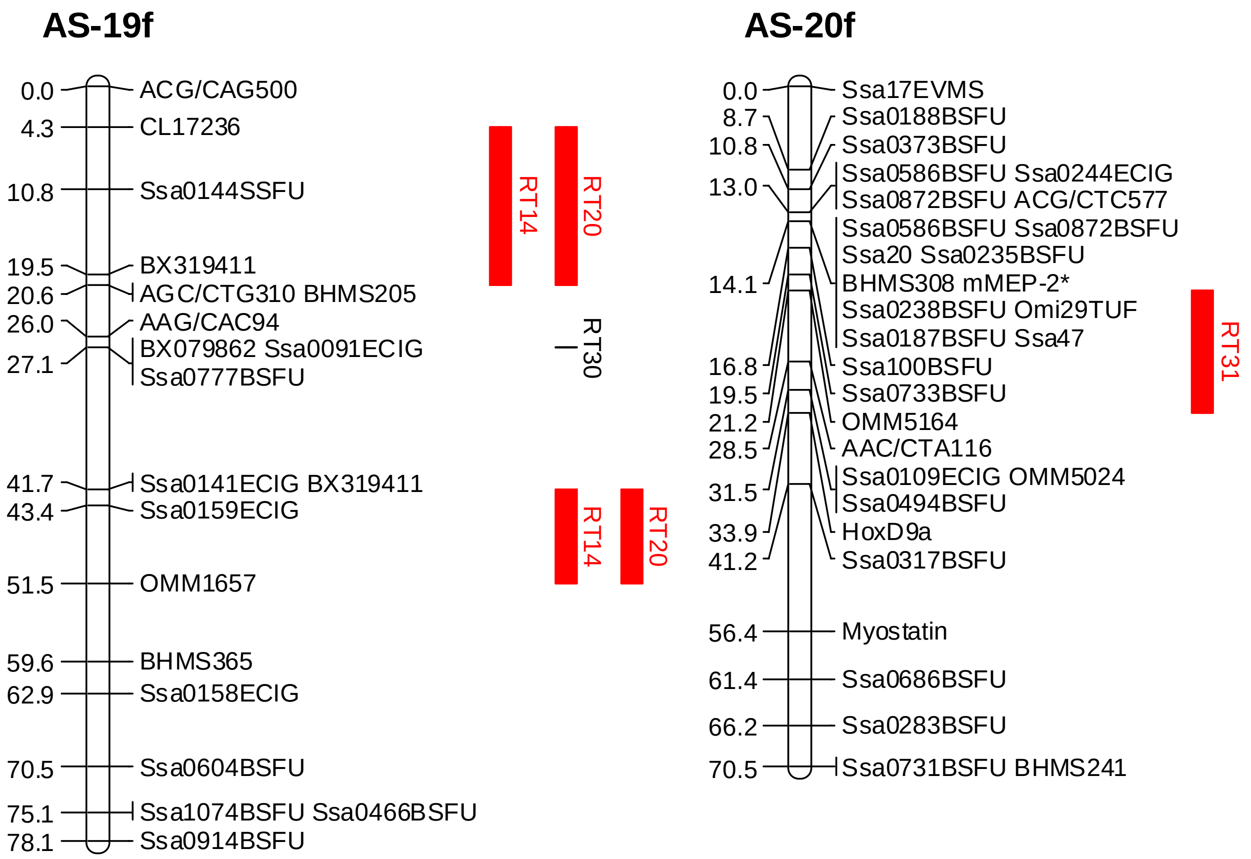
AS-19f
0.0
ACG/CAG500
4.3
CL17236
10.8
Ssa0144SSFU
19.5
BX319411
20.6
AGC/CTG310 BHMS205
26.0
AAG/CAC94
27.1
BX079862 Ssa0091ECIG
Ssa0777BSFU
41.7
Ssa0141ECIG BX319411
43.4
Ssa0159ECIG
51.5
OMM1657
59.6
BHMS365
62.9
Ssa0158ECIG
70.5
Ssa0604BSFU
75.1
Ssa1074BSFU Ssa0466BSFU
78.1
Ssa0914BSFU
RT14
RT20
RT30
RT14
RT20
AS-20f
0.0
Ssa17EVMS
8.7
Ssa0188BSFU
10.8
Ssa0373BSFU
13.0
Ssa0586BSFU Ssa0244ECIG
Ssa0872BSFU ACG/CTC577
14.1
Ssa0586BSFU Ssa0872BSFU
Ssa20 Ssa0235BSFU
BHMS308 mMEP-2*
Ssa0238BSFU Omi29TUF
Ssa0187BSFU Ssa47
16.8
Ssa100BSFU
19.5
Ssa0733BSFU
21.2
OMM5164
28.5
AAC/CTA116
31.5
Ssa0109ECIG OMM5024
Ssa0494BSFU
33.9
HoxD9a
41.2
Ssa0317BSFU
56.4
Myostatin
61.4
Ssa0686BSFU
66.2
Ssa0283BSFU
70.5
Ssa0731BSFU BHMS241
RT31

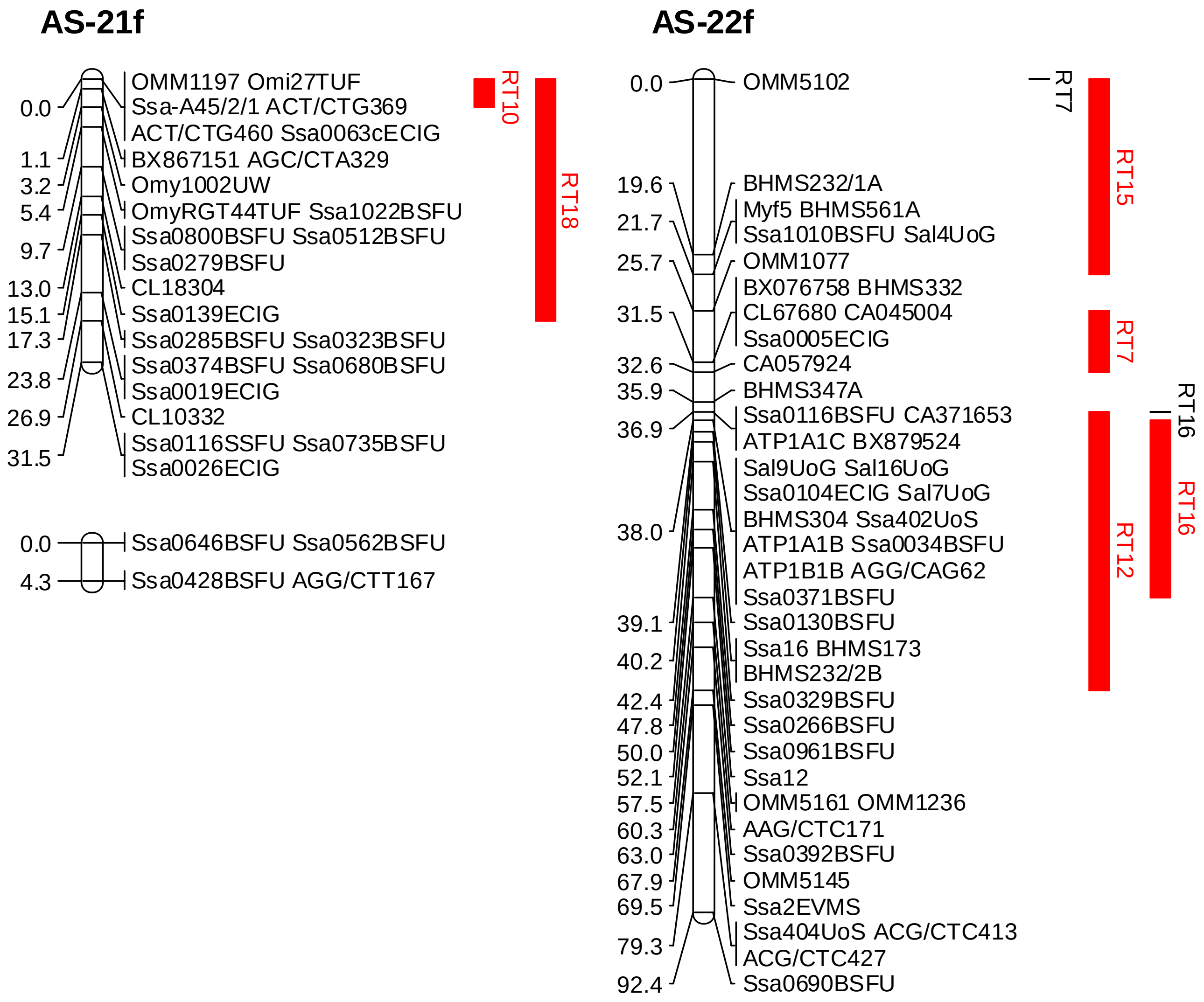
AS-21f
0.0
1.1
3.2
5.4
9.7
13.0
15.1
17.3
23.8
26.9
31.5
OMM1197 Omi27TUF
Ssa-A45/2/1 ACT/CTG369
ACT/CTG460 Ssa0063cECIG
BX867151 AGC/CTA329
Omy1002UW
OmyRGT44TUF Ssa1022BSFU
Ssa0800BSFU Ssa0512BSFU
Ssa0279BSFU
CL18304
Ssa0139ECIG
Ssa0285BSFU Ssa0323BSFU
Ssa0374BSFU Ssa0680BSFU
Ssa0019ECIG
CL10332
Ssa0116SSFU Ssa0735BSFU
Ssa0026ECIG
RT10
RT18
0.0
4.3
Ssa0646BSFU Ssa0562BSFU
Ssa0428BSFU AGG/CTT167
AS-22f
0.0
19.6
21.7
25.7
31.5
32.6
35.9
36.9
38.0
39.1
40.2
42.4
47.8
50.0
52.1
57.5
60.3
63.0
67.9
69.5
79.3
92.4
OMM5102
BHMS232/1A
Myf5 BHMS561A
Ssa1010BSFU Sal4UoG
OMM1077
BX076758 BHMS332
CL67680 CA045004
Ssa0005ECIG
CA057924
BHMS347A
Ssa0116BSFU CA371653
ATP1A1C BX879524
Sal9UoG Sal16UoG
Ssa0104ECIG Sal7UoG
BHMS304 Ssa402UoS
ATP1A1B Ssa0034BSFU
ATP1B1B AGG/CAG62
Ssa0371BSFU
Ssa0130BSFU
Ssa16 BHMS173
BHMS232/2B
Ssa0329BSFU
Ssa0266BSFU
Ssa0961BSFU
Ssa12
OMM5161 OMM1236
AAG/CTC171
Ssa0392BSFU
OMM5145
Ssa2EVMS
Ssa404UoS ACG/CTC413
ACG/CTC427
Ssa0690BSFU
RT7
RT15
RT7
RT16
RT16
RT12

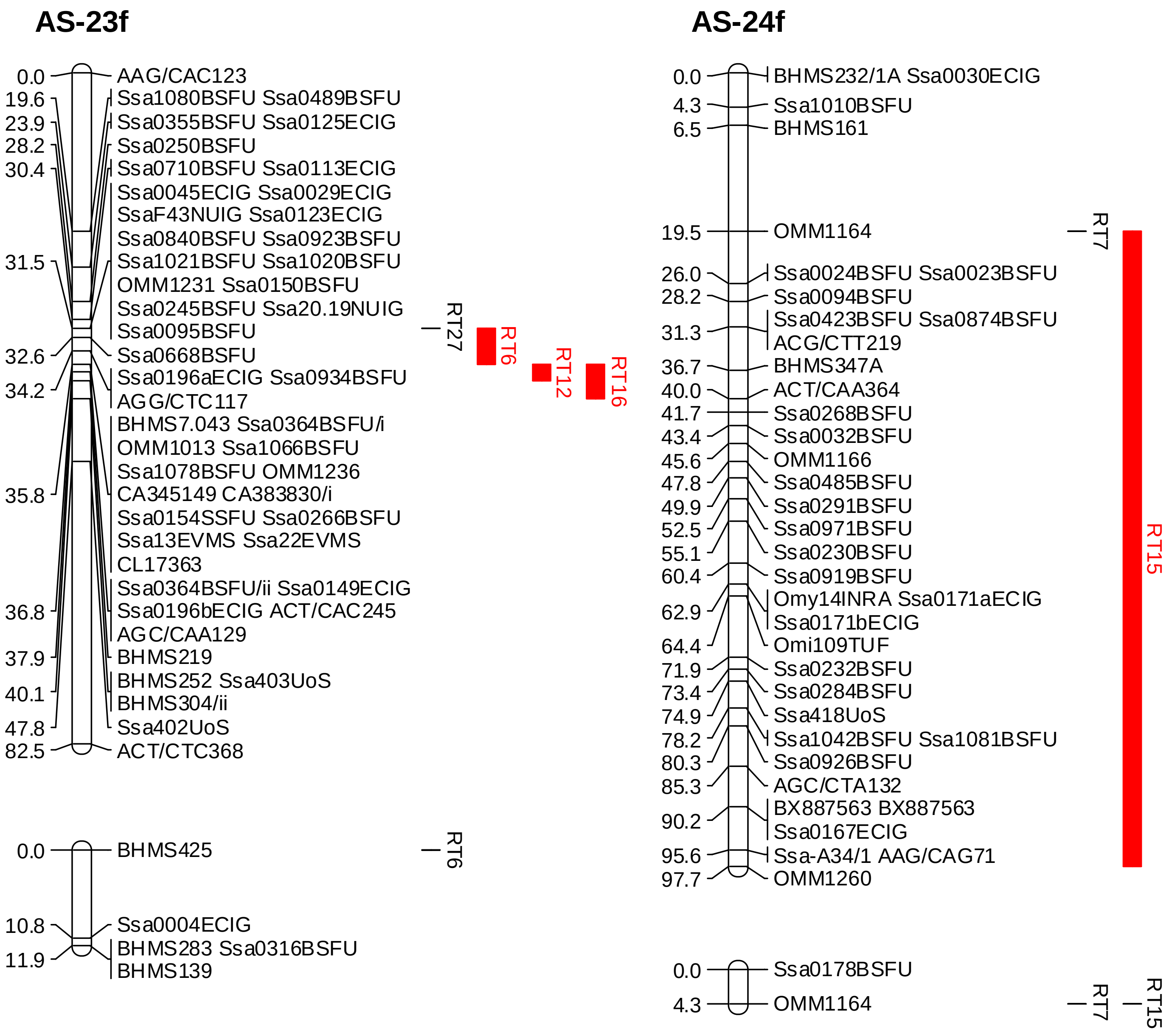
AS-23f
AS-24f
0.0 AAG/CAC123
19.6 Ssa1080BSFU Ssa0489BSFU
23.9 Ssa0355BSFU Ssa0125ECIG
28.2 Ssa0250BSFU
30.4 Ssa0710BSFU Ssa0113ECIG
Ssa0045ECIG Ssa0029ECIG
SsaF43NUIG Ssa0123ECIG
Ssa0840BSFU Ssa0923BSFU
31.5 Ssa1021BSFU Ssa1020BSFU
OMM1231 Ssa0150BSFU
Ssa0245BSFU Ssa20.19NUIG
Ssa0095BSFU
32.6 Ssa0668BSFU
34.2 Ssa0196aECIG Ssa0934BSFU
AGG/CTC117
BHMS7.043 Ssa0364BSFU/i
OMM1013 Ssa1066BSFU
Ssa1078BSFU OMM1236
35.8 CA345149 CA383830/i
Ssa0154SSFU Ssa0266BSFU
Ssa13EVMS Ssa22EVMS
CL17363
Ssa0364BSFU/ii Ssa0149ECIG
36.8 Ssa0196bECIG ACT/CAC245
AGC/CAA129
37.9 BHMS219
BHMS252 Ssa403UoS
40.1 BHMS304/ii
47.8 Ssa402UoS
82.5 ACT/CTC368
RT27
RT6
RT12
RT16
0.0 BHMS425
RT6
10.8 Ssa0004ECIG
BHMS283 Ssa0316BSFU
11.9 BHMS139
0.0 BHMS232/1A Ssa0030ECIG
4.3 Ssa1010BSFU
6.5 BHMS161
19.5 OMM1164
RT7
26.0 Ssa0024BSFU Ssa0023BSFU
28.2 Ssa0094BSFU
31.3 Ssa0423BSFU Ssa0874BSFU
ACG/CTT219
36.7 BHMS347A
40.0 ACT/CAA364
41.7 Ssa0268BSFU
43.4 Ssa0032BSFU
45.6 OMM1166
47.8 Ssa0485BSFU
49.9 Ssa0291BSFU
52.5 Ssa0971BSFU
55.1 Ssa0230BSFU
60.4 Ssa0919BSFU
62.9 Omy14INRA Ssa0171aECIG
Ssa0171bECIG
64.4 Omi109TUF
71.9 Ssa0232BSFU
73.4 Ssa0284BSFU
74.9 Ssa418UoS
78.2 Ssa1042BSFU Ssa1081BSFU
80.3 Ssa0926BSFU
85.3 AGC/CTA132
BX887563 BX887563
90.2 Ssa0167ECIG
95.6 Ssa-A34/1 AAG/CAG71
97.7 OMM1260
RT15
0.0 Ssa0178BSFU
4.3 OMM1164
RT7
RT15

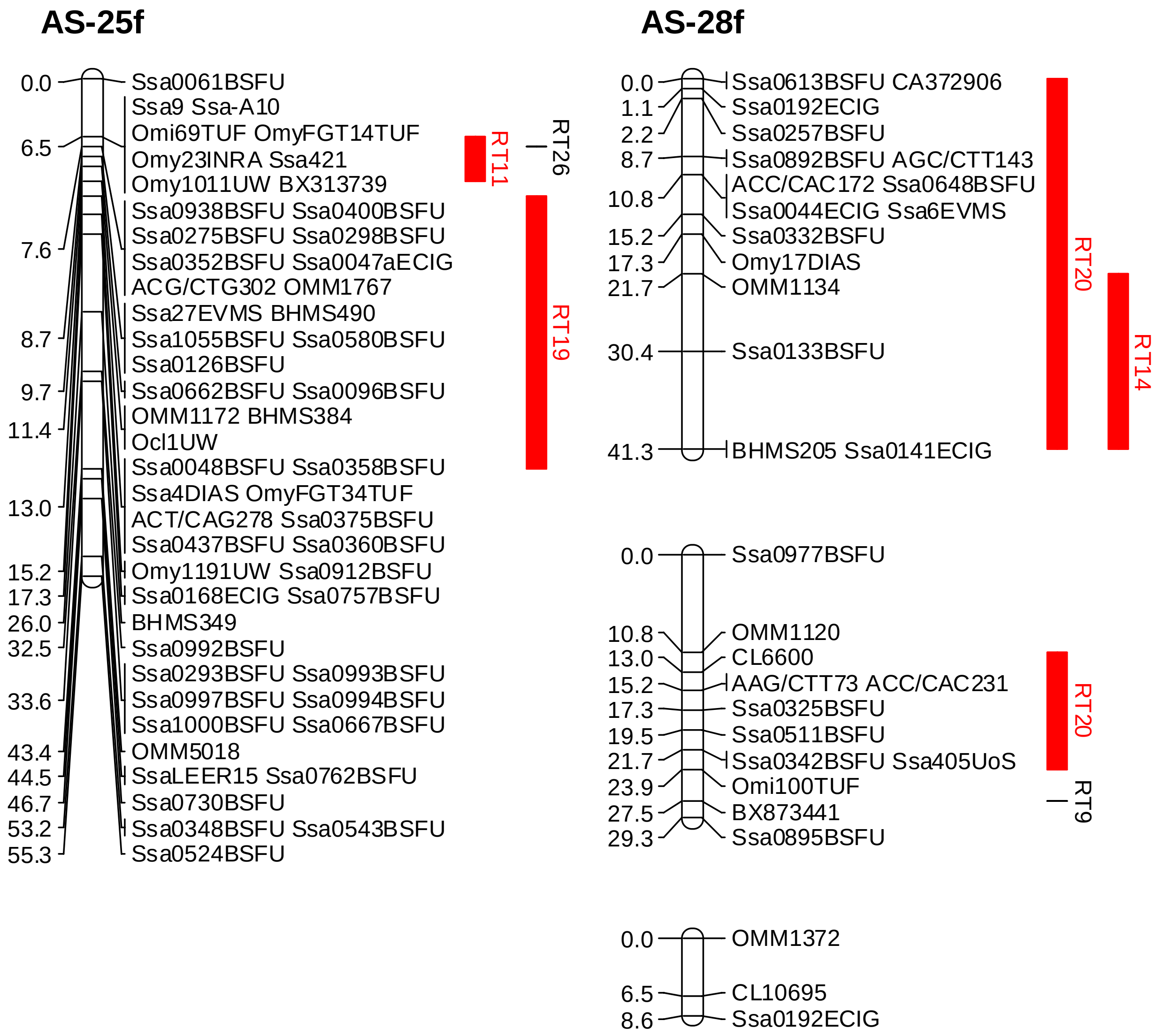

AS-25f
0.0
Ssa0061BSFU
Ssa9 Ssa-A10
Omi69TUF OmyFGT14TUF
6.5
Omy23INRA Ssa421
Omy1011UW BX313739
Ssa0938BSFU Ssa0400BSFU
Ssa0275BSFU Ssa0298BSFU
7.6
Ssa0352BSFU Ssa0047aECIG
ACG/CTG302 OMM1767
Ssa27EVMS BHMS490
8.7
Ssa1055BSFU Ssa0580BSFU
Ssa0126BSFU
9.7
Ssa0662BSFU Ssa0096BSFU
OMM1172 BHMS384
11.4
Ocl1UW
Ssa0048BSFU Ssa0358BSFU
Ssa4DIAS OmyFGT34TUF
13.0
ACT/CAG278 Ssa0375BSFU
Ssa0437BSFU Ssa0360BSFU
15.2
Omy1191UW Ssa0912BSFU
17.3
Ssa0168ECIG Ssa0757BSFU
26.0
BHMS349
32.5
Ssa0992BSFU
Ssa0293BSFU Ssa0993BSFU
33.6
Ssa0997BSFU Ssa0994BSFU
Ssa1000BSFU Ssa0667BSFU
43.4
OMM5018
44.5
SsaLEER15 Ssa0762BSFU
46.7
Ssa0730BSFU
53.2
Ssa0348BSFU Ssa0543BSFU
55.3
Ssa0524BSFU
RT11
RT26
RT19
AS-28f
0.0
Ssa0613BSFU CA372906
1.1
Ssa0192ECIG
2.2
Ssa0257BSFU
8.7
Ssa0892BSFU AGC/CTT143
10.8
ACC/CAC172 Ssa0648BSFU
Ssa0044ECIG Ssa6EVMS
15.2
Ssa0332BSFU
17.3
Omy17DIAS
21.7
OMM1134
30.4
Ssa0133BSFU
41.3
BHMS205 Ssa0141ECIG
RT20
RT14
0.0
Ssa0977BSFU
10.8
OMM1120
13.0
CL6600
15.2
AAG/CTT73 ACC/CAC231
17.3
Ssa0325BSFU
19.5
Ssa0511BSFU
21.7
Ssa0342BSFU Ssa405UoS
23.9
Omi100TUF
27.5
BX873441
29.3
Ssa0895BSFU
RT20
RT9
0.0
OMM1372
6.5
CL10695
8.6
Ssa0192ECIG

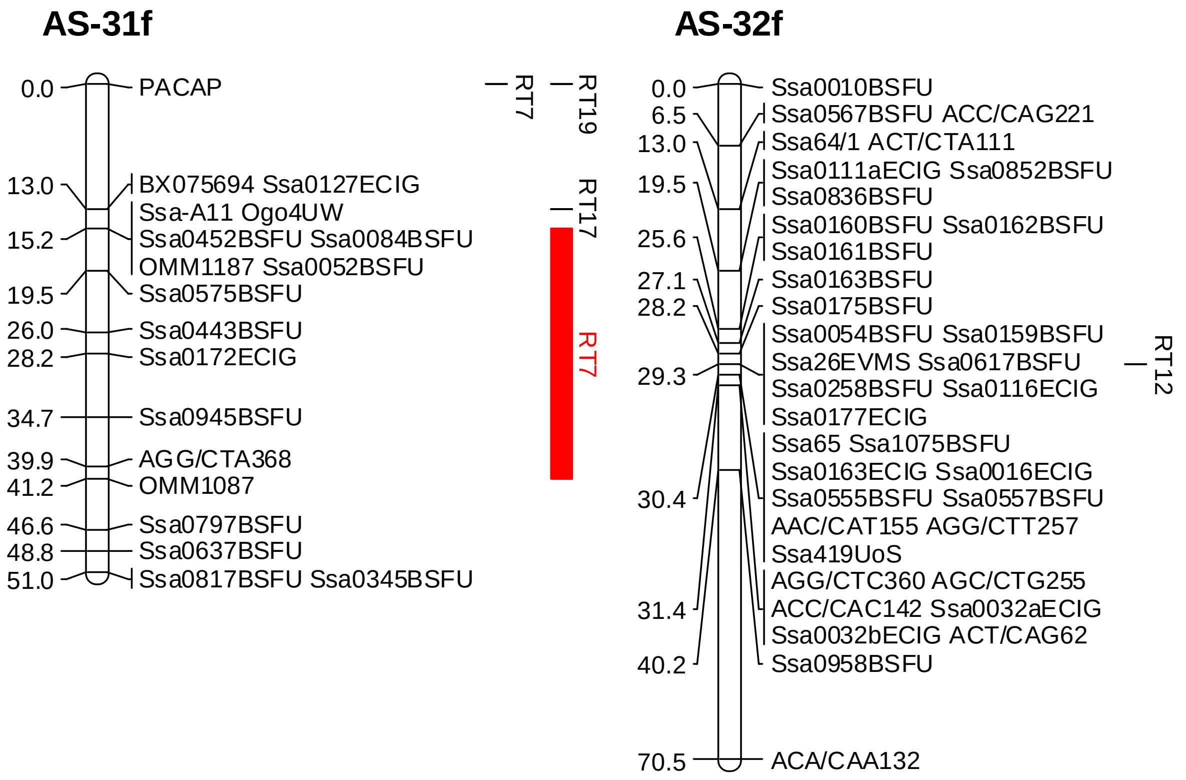
AS-31f
0.0 PACAP
13.0 BX075694 Ssa0127ECIG
Ssa-A11 Ogo4UW
15.2 Ssa0452BSFU Ssa0084BSFU
OMM1187 Ssa0052BSFU
19.5 Ssa0575BSFU
26.0 Ssa0443BSFU
28.2 Ssa0172ECIG
34.7 Ssa0945BSFU
39.9 AGG/CTA368
41.2 OMM1087
46.6 Ssa0797BSFU
48.8 Ssa0637BSFU
51.0 Ssa0817BSFU Ssa0345BSFU
RT7
RT19
RT17
RT7
AS-32f
0.0 Ssa0010BSFU
6.5 Ssa0567BSFU ACC/CAG221
13.0 Ssa64/1 ACT/CTA111
19.5 Ssa0111aECIG Ssa0852BSFU
Ssa0836BSFU
25.6 Ssa0160BSFU Ssa0162BSFU
Ssa0161BSFU
27.1 Ssa0163BSFU
28.2 Ssa0175BSFU
Ssa0054BSFU Ssa0159BSFU
29.3 Ssa26EVMS Ssa0617BSFU
Ssa0258BSFU Ssa0116ECIG
Ssa0177ECIG
Ssa65 Ssa1075BSFU
Ssa0163ECIG Ssa0016ECIG
30.4 Ssa0555BSFU Ssa0557BSFU
AAC/CAT155 AGG/CTT257
Ssa419UoS
AGG/CTC360 AGC/CTG255
31.4 ACC/CAC142 Ssa0032aECIG
Ssa0032bECIG ACT/CAG62
40.2 Ssa0958BSFU
70.5 ACA/CAA132
RT12

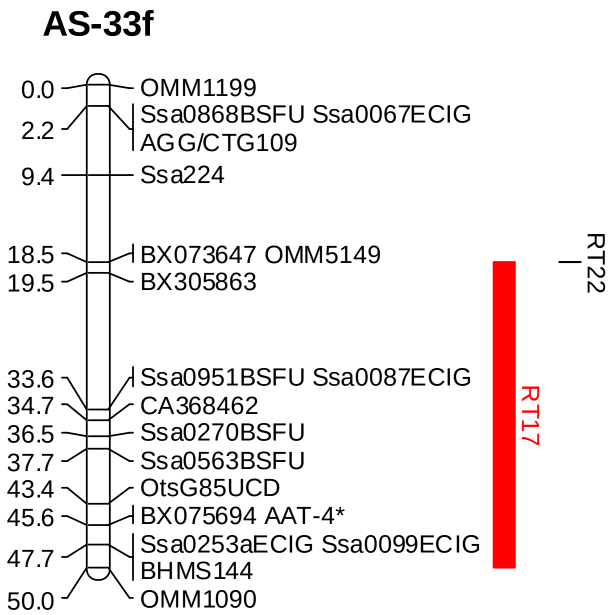
AS-33f
0.0
OMM1199
2.2
Ssa0868BSFU Ssa0067ECIG
AGG/CTG109
9.4
Ssa224
18.5
BX073647 OMM5149
19.5
BX305863
33.6
Ssa0951BSFU Ssa0087ECIG
34.7
CA368462
36.5
Ssa0270BSFU
37.7
Ssa0563BSFU
43.4
OtsG85UCD
45.6
BX075694 AAT-4*
47.7
Ssa0253aECIG Ssa0099ECIG
BHMS144
50.0
OMM1090
RT22
RT17
