# Supplementary figures and images for: Distribution of ancestral proto-Actinopterygian chromosome arms within the genomes of 4R-derivative salmonid fishes (Rainbow trout and Atlantic salmon)
Source: BMC Genomics. 2008 Nov 25;9:557. doi: 10.1186/1471-2164-9-557 (PMC2632648; doi:10.1186/1471-2164-9-557)

## Slide 1
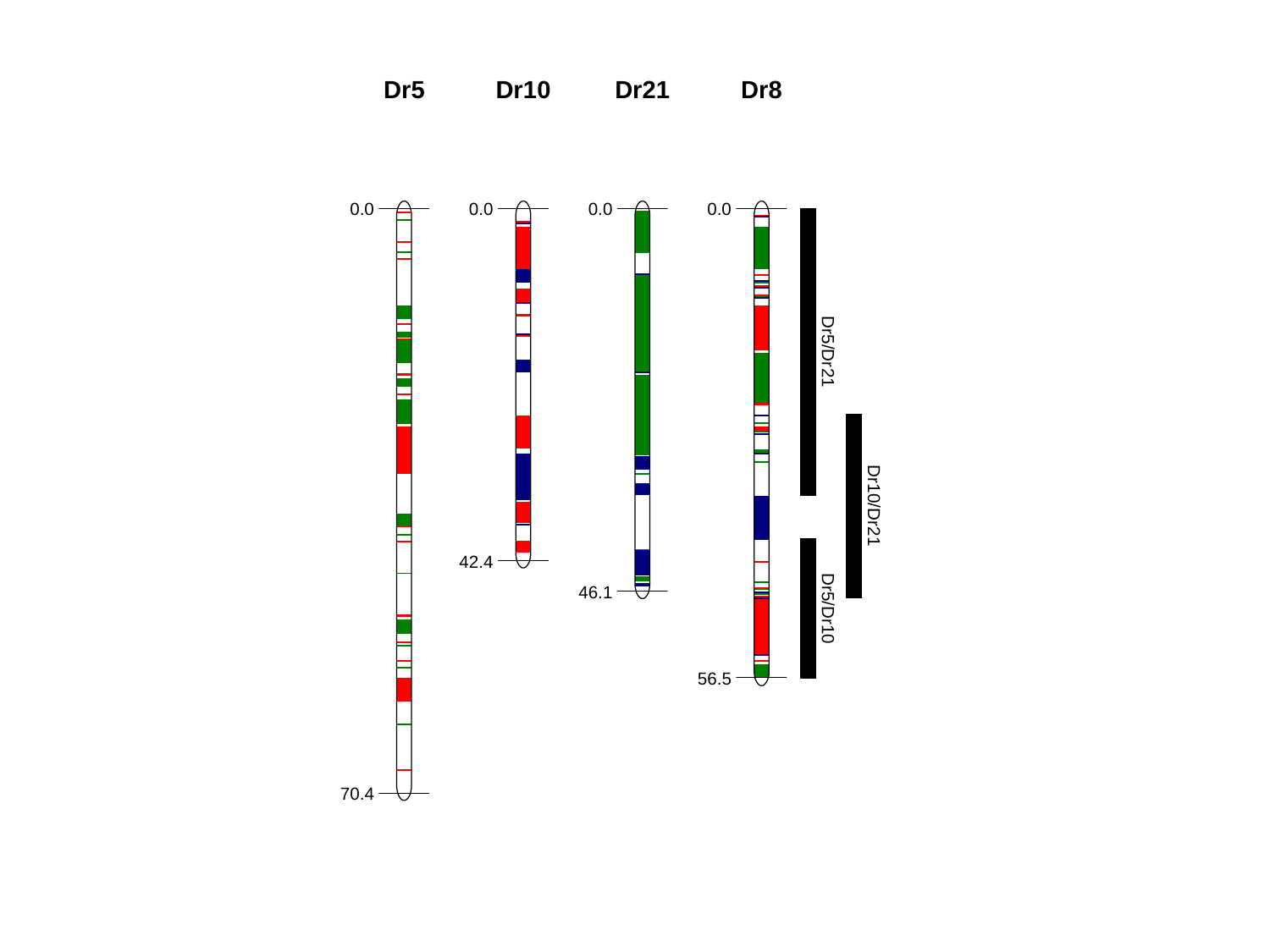

Supplement: Additional File 15 — supplementary figure 3.ppt. Regions of shared homology among zebrafish chromosomes 5, 10 and 21, and their shared affinities with zebrafish chromosome 8. [file 1471-2164-9-557-S15.ppt]

## Slide 1
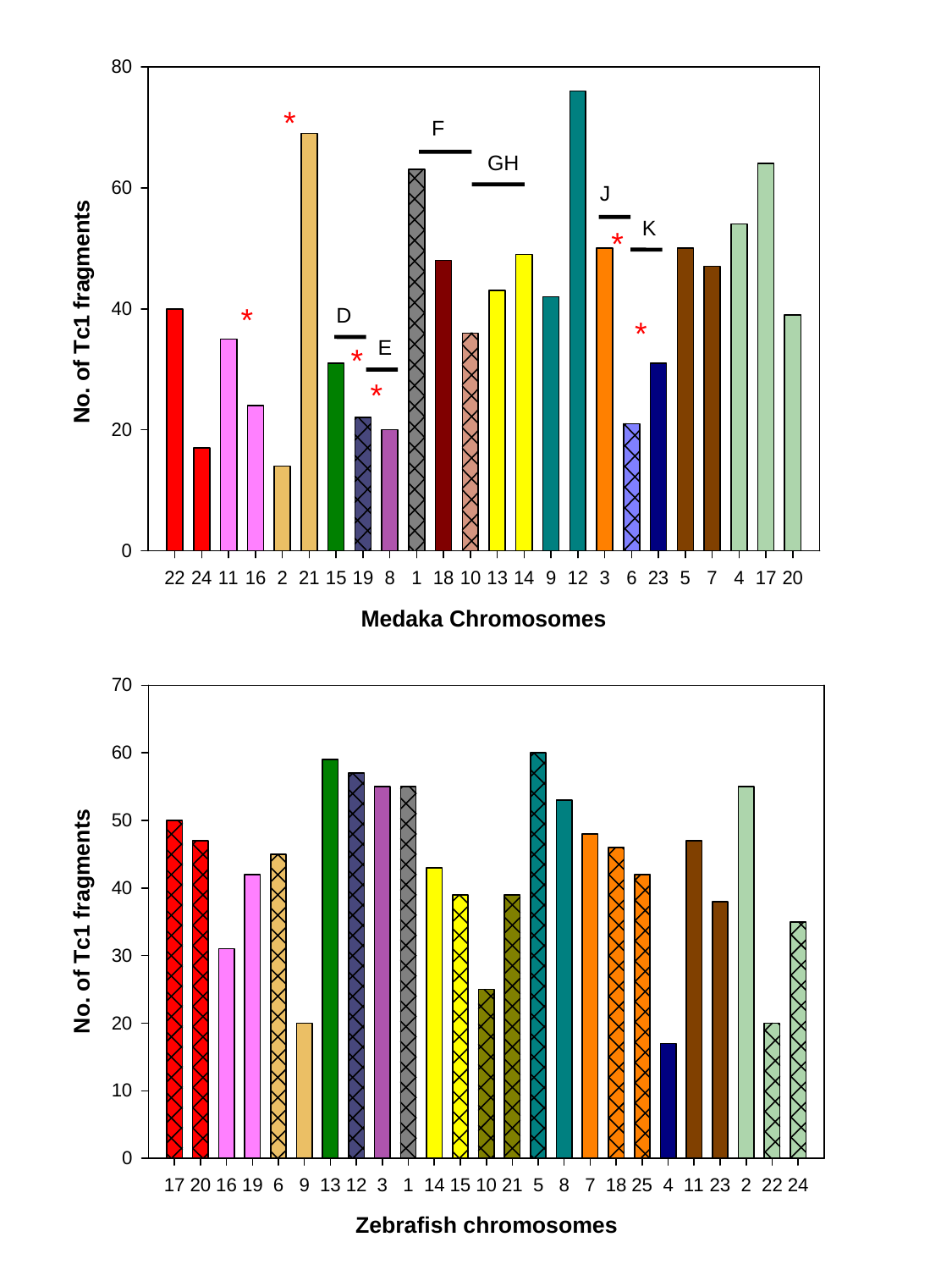

*
F
GH
J
K
*
*
D
*
E
*
*

Supplement: Additional File 18 — supplementary figure 4.ppt. Number of Tc1/mariner transposon hits to zebrafish and medaka linkage groups. [file 1471-2164-9-557-S18.ppt]

## Slide 1
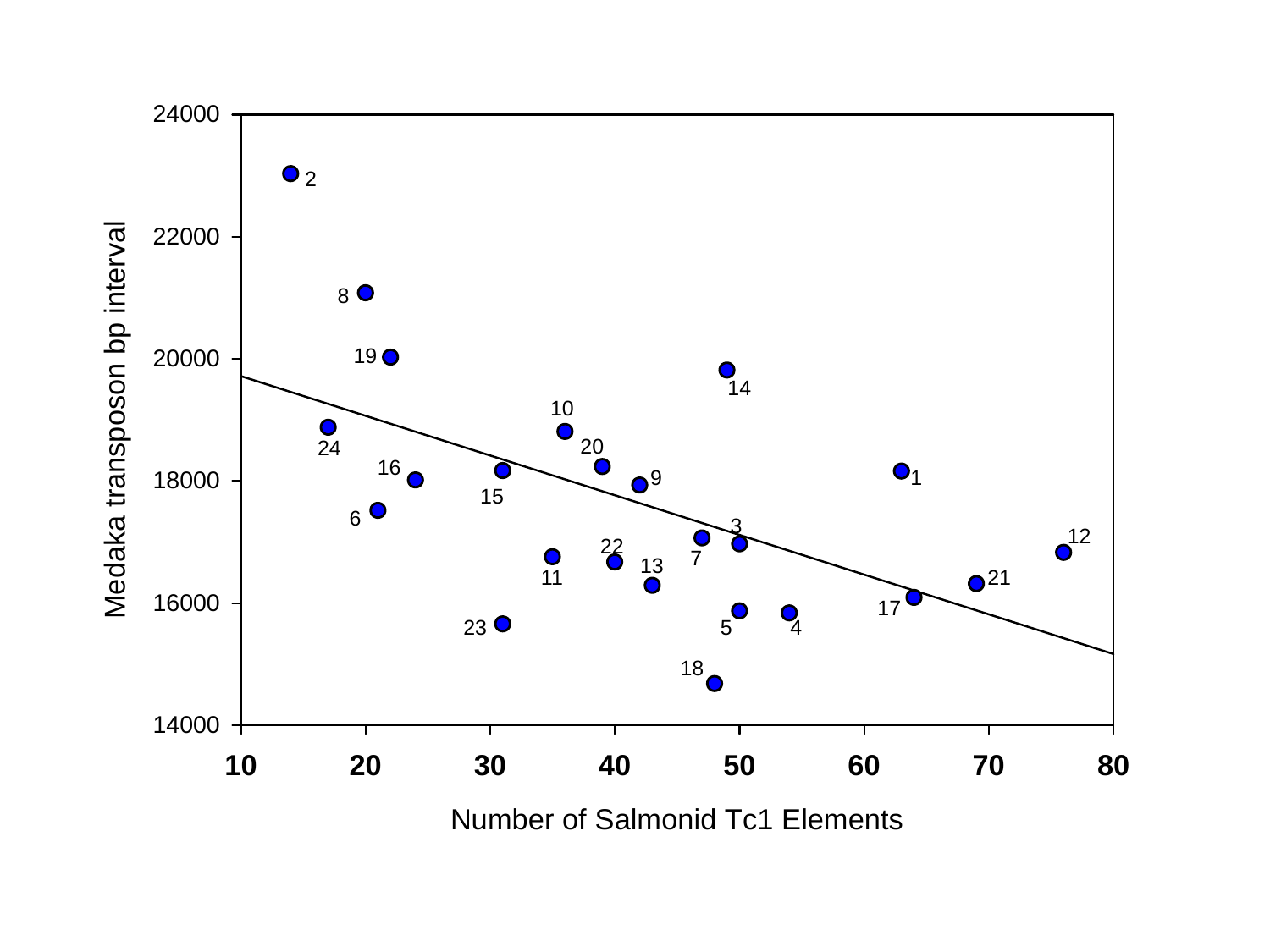

2
8
 19
14
10
20
24
16
9
1
15
 6
3
12
22
7
13
11
21
17
 23
5
4
18

Supplement: Additional File 19 — supplementary figure 5.ppt. Average bp interval among all DNA/LINE/SINE/LTR transposons detected among medaka linkage groups. [file 1471-2164-9-557-S19.ppt]
